# Comparative transcriptomic analysis reveals common molecular factors responsive to heat and drought stress in *Agrostis stolonifera*

**DOI:** 10.1038/s41598-018-33597-3

**Published:** 2018-10-12

**Authors:** Yi Xu, Bingru Huang

**Affiliations:** Department of Plant Biology, Rutgers University, New Brunswick, NJ 08901 USA

## Abstract

Heat and drought stress are primary abiotic stresses confining growth of cool-season grass species during summer. The objective of this study was to identify common molecular factors and metabolic pathways associated with heat and drought responses in creeping bentgrass (*Agrostis stolonifera*) by comparative analysis of transcriptomic profiles between plants exposed to heat and drought stress. Plants were exposed to heat stress (35/30 °C day/night temperature) or drought stress by withholding irrigation for 21 d in growth chambers. Transcriptomic profiling by RNA-seq in *A. stolonifera* (cv. ‘Penncross’) found 670 commonly up-regulated and 812 commonly down-regulated genes by heat and drought stress. Transcriptional up-regulations of differentially expressed genes (DEGs) due to heat and drought stress include genes that were highly enriched in oxylipin biosynthetic process and proline biosynthetic process. Transcriptional down-regulations of genes under heat and drought stress were highly enriched and involved in thiamine metabolic process and calcium sensing receptor. These commonly-regulated genes by heat and drought stress identified in *A. stolonifera* suggested that drought and heat responses shared such common molecular factors and pathways, which could be potential candidate genes for genetic modification of improving plant tolerance to the combined heat and drought stress.

## Introduction

Drought and heat stresses are two major abiotic stresses affecting plant growth. Plant physiological and biochemical responses to either stress alone or the combined stress have been reported, such as growth inhibition, and disturbed carbohydrate, amino acid, and hormone metabolism, as well as the induction of oxidative damages^1–8^. Drought and heat stress may affect plant growth by some common mechanisms, and indeed drought and heat are often associated with each other in natural environmental conditions during summer months^9^. However, molecular factors underlying the alteration of physiological performance, which are commonly regulated by drought and heat stresses, are not well documented.

RNA sequencing (RNA-seq) is one of the powerful next generation sequencing technologies that provides large amount of transcriptional information, which is especially useful to detect differential gene regulation patterns under different types of stresses. Transcriptomic analysis by RNA-seq of plant responses to drought stress has been performed in various plant species, and revealed that most of the up-regulated transcripts in response to drought stress were involved in carbohydrate metabolism, oxidative responses and hormone metabolism, while most of down-regulated transcripts are found to be related to photosynthesis^10–15^. Transcriptomic analysis for heat stress responses in various plant species have found significant enrichment of up-regulated genes in carbohydrate metabolism, protein metabolism, lipid metabolism, oxidative stress responses, and calcium signaling pathway, as well as many down-regulated genes in photosynthesis, cell cycle, cell wall biosynthesis, and transcription factor families^11,16–19^. However, only a few studies have examined common transcriptomic responses to heat and drought stress^11,20^. Such information would be valuable in order to find out the potential molecular mechanisms and pathways that commonly regulate plant tolerance to both heat and drought stress.

Creeping bentgrass (*Agrostis stolonifera*), is one of the major cool-season grass species, which is widely used as turf and forage grass in temperate regions. Previous studies have been conducted in creeping bentgrass under either drought or heat stress condition, in order to understand physiological, molecular, transcriptomic and proteomic responses related to drought or heat tolerance^21–25^. However, no information regarding to the common regulation patterns for creeping bentgrass under either drought or heat stress condition is available. The objective of this study was to identify common genes regulated by drought and heat stresses, and the associated metabolic pathways for drought and heat adaptation by comparative analysis of the transcriptomic changes in response to drought or heat stress for a cool-season grass species, creeping bentgrass (*A. stolonifera*), widely used as turf and forage grass in temperate regions.

## Results

### Physiological responses to drought and heat stress

**Figure 1:**
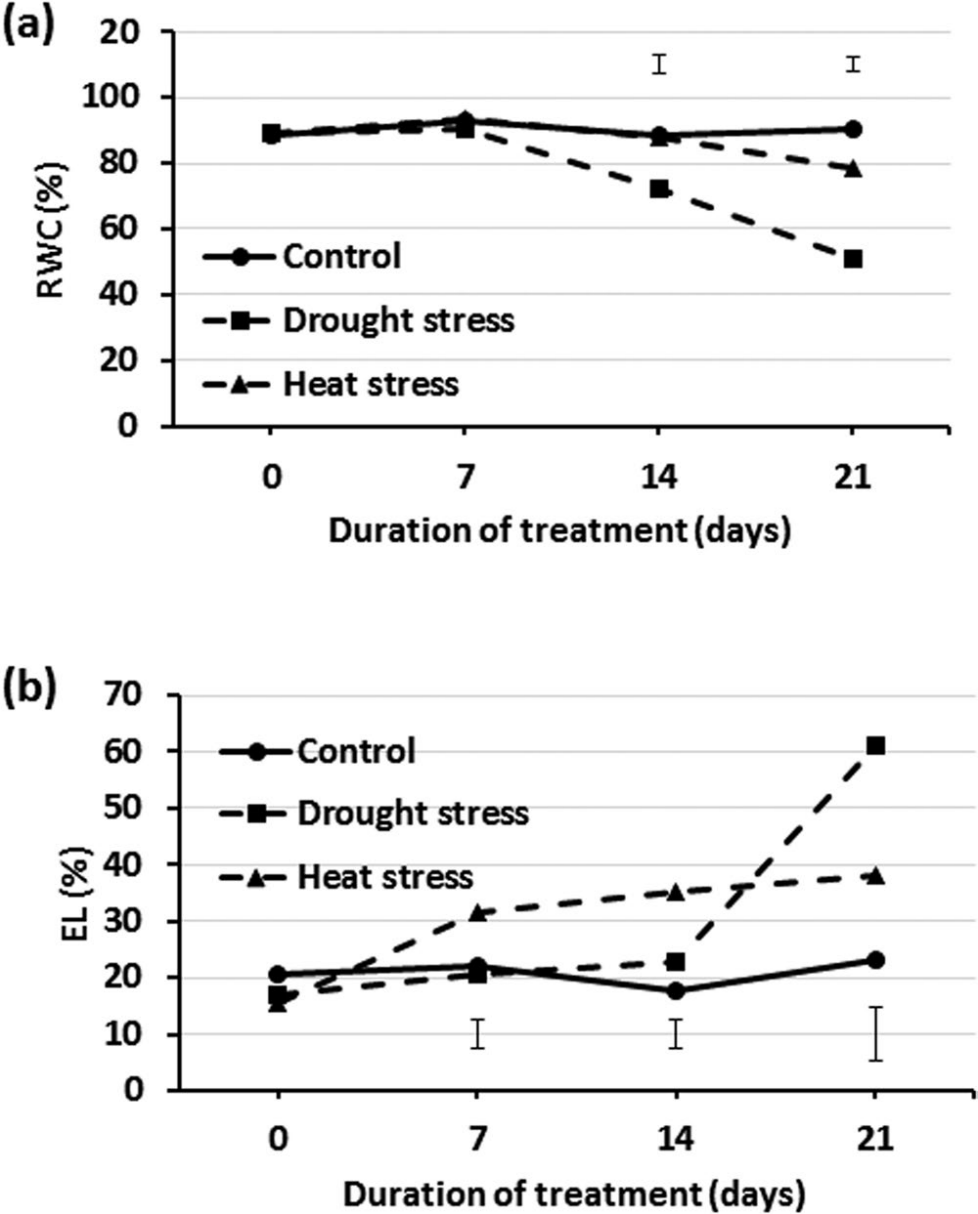
Leaf (**a**) relative water content (RWC) and (**b**) electrolyte leakage (EL) of *A. stolonifera* under non-stress control, drought and heat stress conditions. Data shown are the means of four biological replicates (n = 4). Bar represents Fisher’s least significant difference (LSD) for each sampling day.

Creeping bentgrass plants were subject to either heat (35/30 °C day/night temperature) or drought (withheld from irrigation) for 21 d, respectively. Leaf relative water content (RWC) did not differ significantly at 0 and 7 d between control and either drought or heat stress condition. Drought or heat treatment led to significant decrease of RWC at 14 and 21 d compared with the control (Fig. 1a). For electrolyte leakage (EL), there was no significant difference found at 0 d between the control, drought and heat stress conditions. Heat or drought stress resulted in significant increases of EL compared with the control at 7 and 14 d of heat stress and at 21 d of drought (Fig. 1b).

### Transcriptomic sequencing of *A. stolonifera*

**Figure 2:**
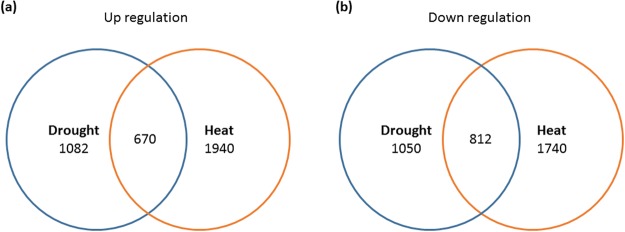
Number of (**a**) up- and (**b**) down-regulated genes in *A. stolonifera* under drought and heat stress, respectively, compared with control condition. The cutoff threshold was set as FDR > 0.001.

The RNA sequencing yielded about 25 million reads per library of *A. stolonifera* leaves exposed to either control, heat or drought stress conditions, providing over 18x coverage of the estimated transcriptome size (~417Mbp) of *A. stolonifera*^26,27^. Using the threshold of FDR <0.001 and |log2 of fold change (FC)| >1, a total of 2132 and 3680 differentially-expressed genes (DEGs) in response to drought or heat stress, respectively were found in *A. stolonifera*. Among them, 1082 and 1940 DEGs were significantly up-regulated under drought and heat stress, respectively, and 1050 and 1740 DEGs were significantly down-regulated under drought and heat stress, respectively (Fig. 2). Among them, 1482 genes were commonly regulated by drought and heat stress in *A. stolonifera*, with 670 up-regulated and 812 down-regulated genes included in both drought and heat stresses (Fig. 2, Table S1 in Supplementary Materials).

### GO term enrichment analysis

**Figure 3:**
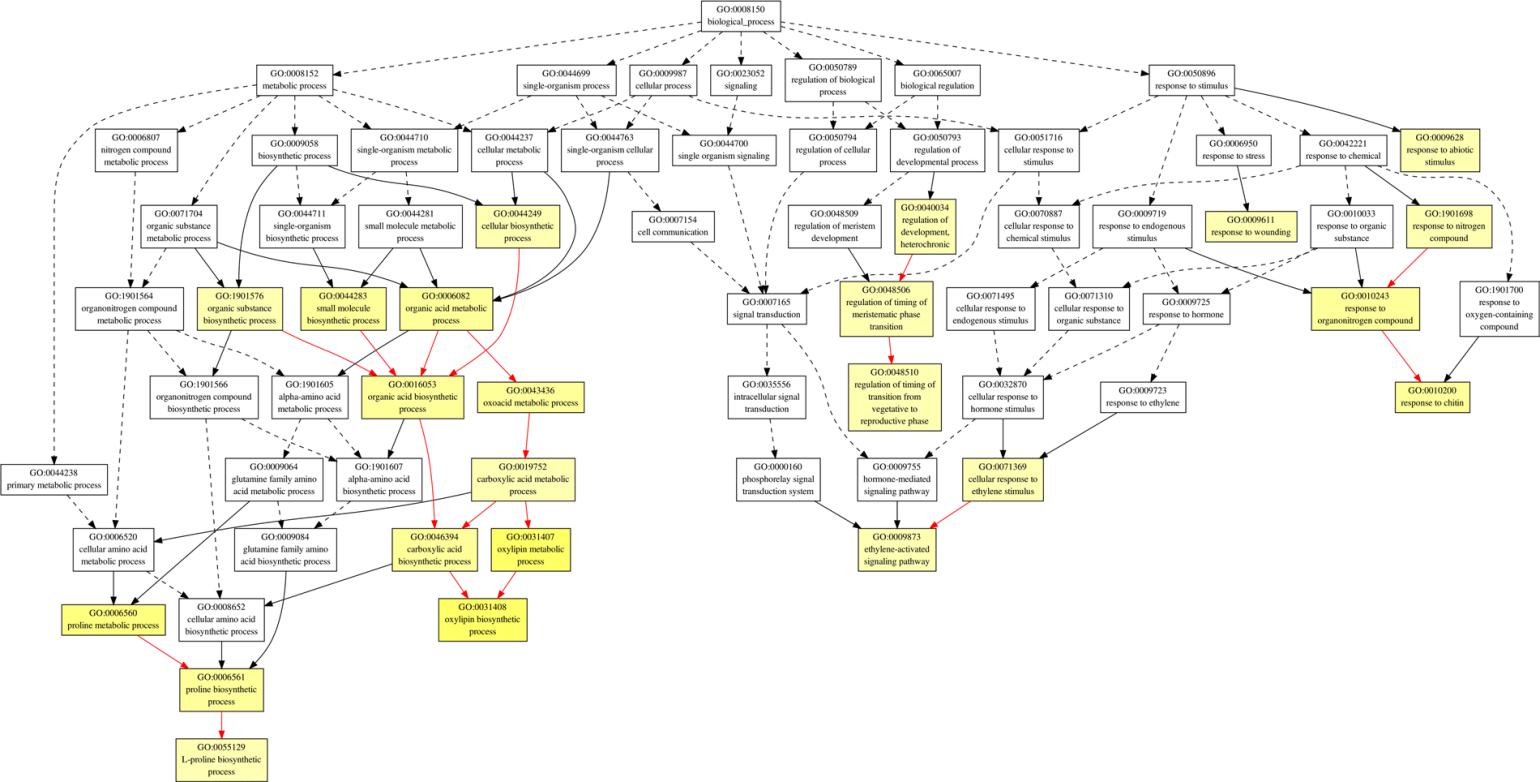
Biological Process (BP) of GO term enrichment for up-regulated common DEGs in *A. stolonifera*. Yellow color indicates significantly enriched GO terms. The density of color in each node was proportional to statistical significance of enrichment of the corresponding GO term. Red edges stand for relationship between enriched GO terms.

**Figure 4:**
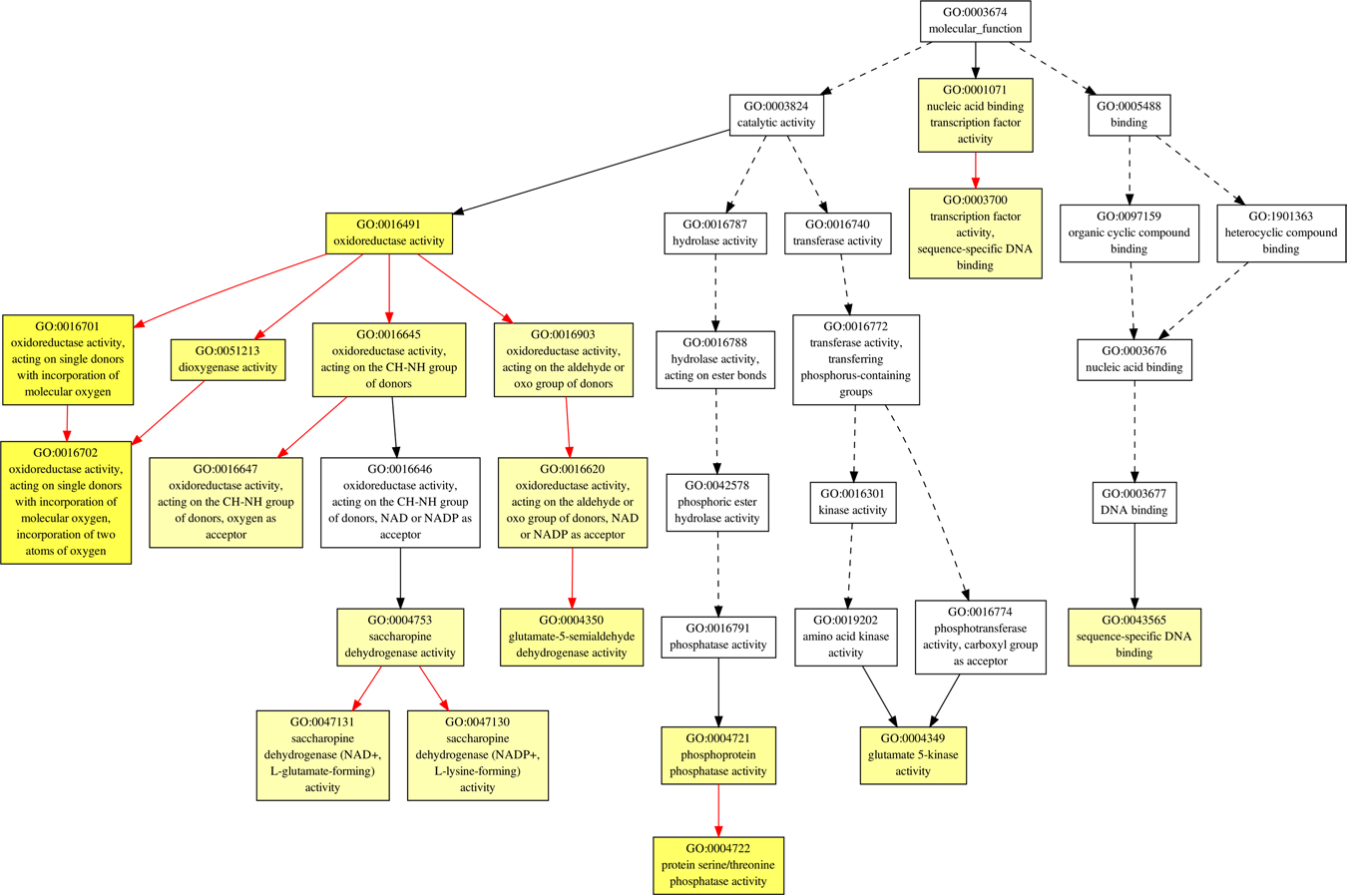
Molecular Function (MF) of GO term enrichment for up-regulated common DEGs in *A. stolonifera*. Yellow color indicates significantly enriched GO terms. The density of color in each node was proportional to statistical significance of enrichment of the corresponding GO term. Red edges stand for relationship between enriched GO terms.

**Figure 5:**
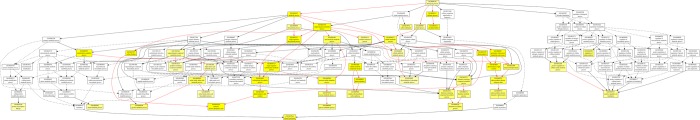
Biological Process (BP) of GO term enrichment for down-regulated common DEGs in *A. stolonifera*. Yellow color indicates significantly enriched GO terms. The density of color in each node was proportional to statistical significance of enrichment of the corresponding GO term. Red edges stand for relationship between enriched GO terms.

**Figure 6:**
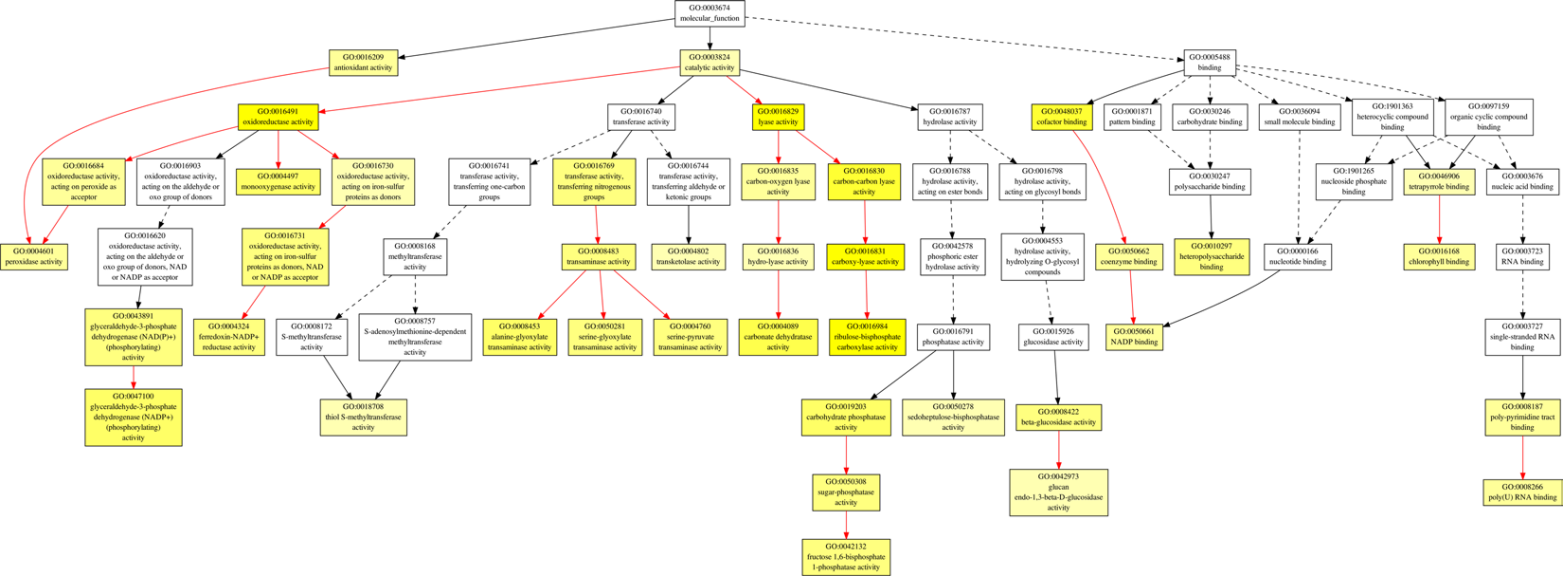
Molecular Function (MF) of GO term enrichment for down-regulated common DEGs in *A. stolonifera*. Yellow color indicates significantly enriched GO terms. The density of color in each node was proportional to statistical significance of enrichment of the corresponding GO term. Red edges stand for relationship between enriched GO terms.

**Figure 7:**
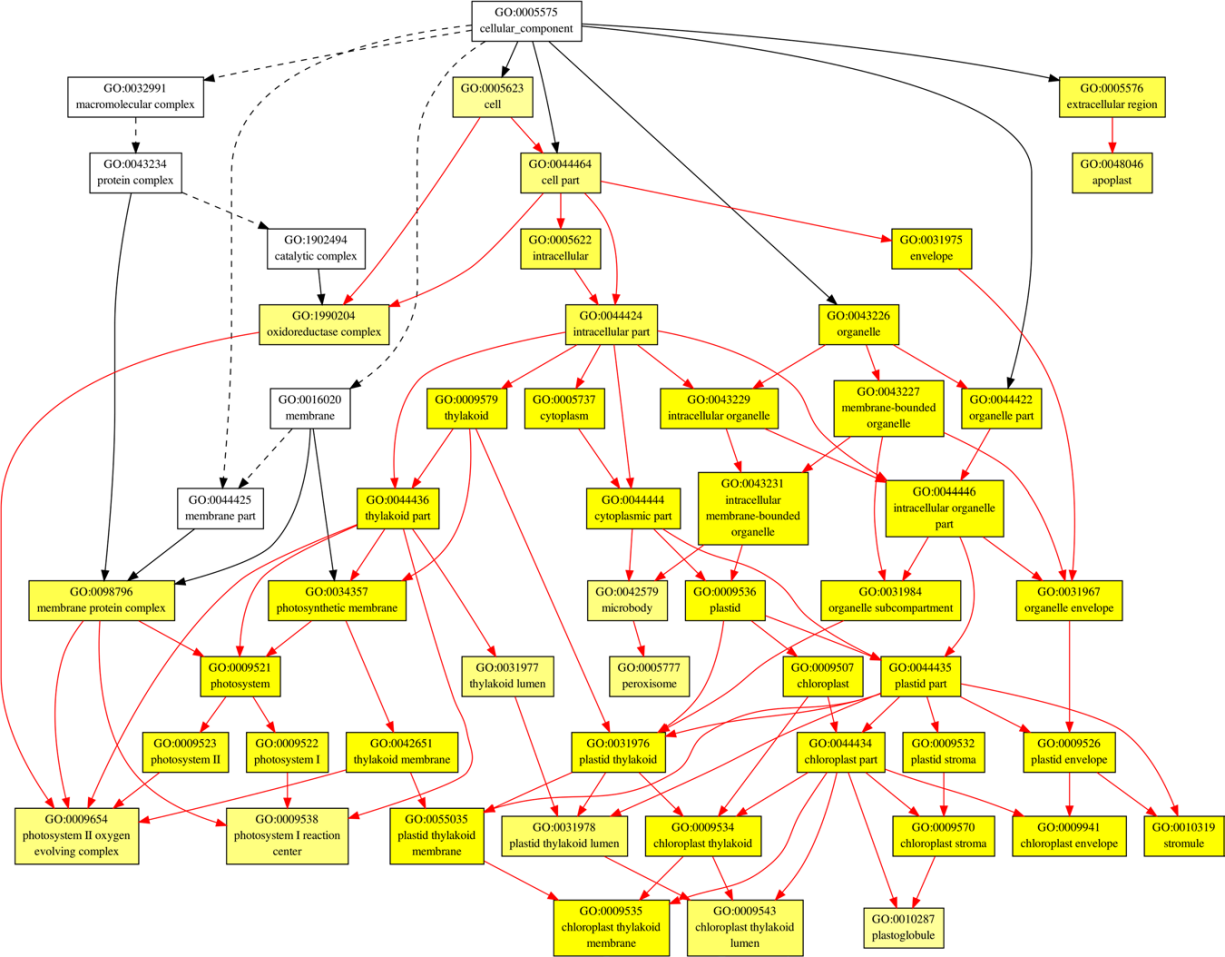
Cellular Component (CC) of GO term enrichment for down-regulated common DEGs in *A. stolonifera*. Yellow color indicates significantly enriched GO terms. The density of color in each node was proportional to statistical significance of enrichment of the corresponding GO term. Red edges stand for relationship between enriched GO terms.

In order to find out the biological processes commonly regulated by drought and heat stress, GO term enrichment analysis was performed for up- and down-regulated common genes (Figs 3–7, Tables 2–3 in Supplementary Materials). In the up-regulated common genes under drought and heat stress, several GO terms in Biological Process (BP) category were mostly enriched, including oxylipin biosynthetic process (GO:0031408), nucleic acid-templated transcription (GO:0097659), transcription, DNA-templated (GO:0006351), RNA biosynthetic process (GO:0032774), nucleobase-containing compound biosynthetic process (GO:0034654), carboxylic acid biosynthetic process (GO:0046394), ethylene-activated signaling pathway (GO:0009873), organic acid biosynthetic process (GO:0016053), hormone-mediated signaling pathway (GO:0009755), tyrosine catabolic process (GO:0006572), proline metabolic process (GO:0006560), RNA metabolic process (GO:0016070), cellular amino acid catabolic process (GO:0009063) and L-proline biosynthesis (GO:0055129) (Fig. 3, Table 1). Among the down-regulated common genes, several GO terms in BP category were enriched under drought and heat stresses, including plastid translation (GO:0032544), thiamine biosynthetic process (GO:0009228), thiamine-containing compound biosynthetic process (GO:0042724), thiamine metabolic process (GO:0006772), thiamine-containing compound metabolic process (GO:0042723), positive regulation of cellular amide metabolic process (GO:0034250), positive regulation of translation (GO:0045727) and serine family amino acid biosynthetic process (GO:0009070), from top to bottom (Figs 5 and 6, Table 2). All the enriched GO terms in cellular component (CC) category under drought and heat stresses were related to chloroplast, including plastid thylakoid (GO:0031976), chloroplast thylakoid (GO:0009534), plastid thylakoid membrane (GO:0055035), chloroplast thylakoid membrane (GO:0009535), chloroplast stroma (GO:0009570), photosystem (GO:0009521), plastid envelope (GO:0009941), stromule (GO:0010319), photosystem II (GO:0009523), photosystem I (GO:0009522), plastid thylakoid lumen (GO:0031978), chloroplast thylakoid lumen (GO:0009543), photosystem II oxygen evolving complex (GO:0009654), photosystem I reaction center (GO:0009538), plastoglobule (GO:0010287) (Fig. 7, Table 3). In regard of molecular function (MF) and CC for up-regulated common genes, and MF for down-regulated common genes, no enriched GO terms were found with the threshold of GO term level ≥6.

**Table 1:** Enriched GO terms in the up-regulated common genes between drought and heat stress condition, using the threshold of GO term level ≥6.

Ontology	GO ID	GO term	Level	P value
Biological_process	GO:0031408	Oxylipin biosynthetic process	8	5.37E-05
Biological_process	GO:0097659	Nucleic acid-templated transcription	9	5.37E-05
Biological_process	GO:0006351	Transcription, DNA-templated	11	5.37E-05
Biological_process	GO:0032774	RNA biosynthetic process	9	5.56E-05
Biological_process	GO:0034654	Nucleobase-containing compound biosynthetic process	9	0.00041
Biological_process	GO:0046394	Carboxylic acid biosynthetic process	7	0.00613
Biological_process	GO:0009873	Ethylene-activated signaling pathway	7	0.00699
Biological_process	GO:0016053	Organic acid biosynthetic process	6	0.00739
Biological_process	GO:0009755	Hormone-mediated signaling pathway	6	0.01119
Biological_process	GO:0006572	Tyrosine catabolic process	11	0.01753
Biological_process	GO:0006560	Proline metabolic process	6	0.03706
Biological_process	GO:0016070	RNA metabolic process	8	0.04209
Biological_process	GO:0009063	Cellular amino acid catabolic process	9	0.04287
Biological_process	GO:0055129	L-proline biosynthetic process	10	0.06368

**Table 2:** Enriched GO terms in the down-regulated common genes between drought and heat stress condition, using the threshold of GO term level ≥6.

Ontology	GO ID	GO term	Level	P value
Biological_process	GO:0032544	Plastid translation	9	2.76e-05
Biological_process	GO:0009228	Thiamine biosynthetic process	9	0.000839
Biological_process	GO:0042724	Thiamine-containing compound biosynthetic process	9	0.000839
Biological_process	GO:0006772	Thiamine metabolic process	7	0.001344
Biological_process	GO:0042723	Thiamine-containing compound metabolic process	7	0.001344
Biological_process	GO:0034250	Positive regulation of cellular amide metabolic process	7	0.003796
Biological_process	GO:0045727	Positive regulation of translation	12	0.003796
Biological_process	GO:0009070	Serine family amino acid biosynthetic process	9	0.095477
Cellular_component	GO:0031976	Plastid thylakoid	7	7.08e-46
Cellular_component	GO:0009534	Chloroplast thylakoid	8	7.08e-46
Cellular_component	GO:0055035	Plastid thylakoid membrane	8	2.28e-41
Cellular_component	GO:0009535	Chloroplast thylakoid membrane	10	2.28e-41
Cellular_component	GO:0009570	Chloroplast stroma	7	4.69e-23
Cellular_component	GO:0009521	Photosystem	8	1.76e-15
Cellular_component	GO:0009526	Plastid envelope	7	6.23e-15
Cellular_component	GO:0009941	Chloroplast envelope	8	7.32e-14
Cellular_component	GO:0010319	Stromule	8	2.42e-12
Cellular_component	GO:0009523	Photosystem II	8	6.59e-08
Cellular_component	GO:0009522	Photosystem I	8	1.80e-07
Cellular_component	GO:0031978	Plastid thylakoid lumen	9	8.15e-05
Cellular_component	GO:0009543	Chloroplast thylakoid lumen	10	8.15e-05
Cellular_component	GO:0009654	Photosystem II oxygen evolving complex	10	0.000555
Cellular_component	GO:0009538	Photosystem I reaction center	10	0.000562
Cellular_component	GO:0010287	Plastoglobule	8	0.003936

**Table 3:** The qRT-PCR validation of selected genes in the enriched GO terms.

Gene symbol	Log_2_FC in qPCR		Log_2_FC in RNA-seq	
	Drought	Heat	Drought	Heat
LOX1	1.86	3.53	3.19	4.75
PLP2	2.33	0.74	3.16	3.35
P5CS	3.73	1.95	3.72	2.31
NECD	3.83	1.25	5.74	4.84
KCS11	1.52	1.85	3.82	3.62
TTS2	−1.16	−1.79	−2.37	−1.79
PMPS	−0.48	−2.79	−2.08	−2.34
CSLB41B	−5.92	−8.35	−4.39	−4.29
CSR	−5.92	−8.35	−1.61	−2.13
NQO5	−2.63	−3.61	−2.72	−2.89
OEE1	−0.78	−0.56	−1.83	−1.11
CYP37	−1.03	−0.75	−3.02	−2.39
PNL1	−2.84	−4.45	−1.74	−2.81
PI4	−1.01	−2.63	−1.16	−1.90
PSBY	−1.21	−0.95	−1.70	−3.17
CUR1A	−1.66	−3.68	−4.56	−6.25
LOW1	−2.52	−1.13	−1.84	−1.05
PSBP3	−2.48	−2.22	−2.84	−2.59
RHO4	−3.52	−2.13	−2.19	−2.15
Pearson’s Correlation for Drought	0.83			
Pearson’s Correlation for Heat	0.76			

### Validation of RNA-seq with qRT-PCR

The differential expressions of several common genes in selected GO terms in the RNA-seq data were validated using qRT-PCR, based on mostly enriched categories in GO term enrichment analysis. For oxylipin biosynthetic process, linoleate 9S-lipoxygenase 1 (LOX1) and patatin-like protein 2 (PAT2) showed up-regulations under drought and heat stress. For proline biosynthetic process, delta-1-pyrroline-5-carboxylate synthase (P5CS) gene expression level was also up-regulated under drought and heat stress conditions. Similarly, 9-cis-epoxycarotenoid dioxygenase 1 (NCED1) in organic acid biosynthetic process, and 3-ketoacyl-CoA synthase 11 (KCS11) were also up-regulated under drought and heat stress conditions (Table 3).

qPCR also confirmed that thiamine thiazole synthase 2 (TTS2) in thiamine biosynthetic process, phosphomethylpyrimidine synthase (PMPS) in thiamine metabolic process, chloroplast stem-loop binding protein of 41 kDa b (CSLB41B) in positive regulation of translation, and calcium sensing receptor, chloroplastic (CSR) were down-regulated under drought and heat stresses. Several genes related to photosystem I and II and the location of chloroplast thylakoid were confirmed to be down-regulated under drought and heat stresses, including NAD(P)H-quinone oxidoreductase subunit 5 (NQO5), oxygen-evolving enhancer protein 1 (OEE1), peptidyl-prolyl cis-trans isomerase (CYP37), photosynthetic NDH subunit of luminal location 1 (PNL1), photosystem I reaction center subunit IV (PI4), photosystem II core complex protein (PSBY), protein CURVATURE THYLAKOID 1 A (CUR1A), protein LOW PSII ACCUMULATION 1 (LOW1), PsbP domain-containing protein 3 (PSBP3), and Rhodanese-like domain-containing protein 4 (RHO4) (Table 3).

Overall, when comparing gene expression patterns detected by RNA-seq and qRT-PCR, the Pearson’s correlation for transcriptional regulation (log2 fold change) under drought stress between RNA-seq and qRT-PCR is 0.83, and the Pearson’s correlation under heat stress between RNA-seq and qRT-PCR is 0.76 (Table 3).

## Discussion

As previously reported, large number of genes are responsive to either drought or heat stress (see Introduction). This study focused on the comparative analysis of transcriptome profiles of *A. stolonifera* exposed to drought and heat stress with the intention to identify genes commonly regulated by heat and drought stress or responsive to both stresses in the same patterns. Among the total of 2132 and 3680 drought- and heat-responsive genes, 1482 genes were commonly regulated by heat and drought, including 670 up-regulated and 812 down-regulated genes. Those commonly-regulated genes by heat and drought stress may play roles in plant adaptation to both stresses in terms of the biological functions and common metabolic pathways, as discussed in details below.

The most enriched GO term in commonly up-regulated transcripts in BP was oxylipin biosynthetic process (GO:0031408, Fig. 3, Table 1). Further analysis showed that these commonly up-regulated transcripts in *A. stolonifera* under drought and heat stresses are lipoxygenase 1 (LOX1) and patatin-like protein 2 (PAT2), which are both involved in oxylipin biosynthetic process. Oxylipins, such as jasmonic acid (JA), methyl jasmonate (MeJA), and traumatin, catalyzed from linoleic or linolenic acid, have been implicated in a variety of physiological processes, plant pathogen resistance and abiotic stress responses^28,29^. Lipoxygenases (LOXs, EC1.13.11.12) are a group of non-heme iron-containing dioxygenases, which serve in the initial step of degradations of free fatty acids and esterified lipids via LOX pathway; the LOX proteins add an oxygen to either end of a (Z,Z)-1,4-pentadiene system of polyunsaturated fatty acids to produce an unsaturated fatty acid hydroperoxide^30^. Under drought stress, LOX enzyme activity increased in olive tree (*Olea europaea*)^31^, but decreased in chive (*Allium schoenoprasum*)^32^. Enhanced LOX activities were found in *Phalaenopsis*^33^ and wheat (*Triticum aestivum*)^34^ under heat stress, which were considered to be correlated with ROS accumulation, lipid peroxidation, and increase of cytoplasmic lipid droplets. Patatin was originally defined as the major storage protein, which accounted for 40% of total soluble protein in potato (*Solanum tuberosum*) tubers^35^. Patatin was also found to exhibit lipid acyl hydrolase (LAH) activity^36^, and has been proven to be transcriptionally regulated by drought stress^37^. A few studies found that patatin-like transcript levels were stimulated upon drought stress^38–40^. Under heat stress, accumulation of patatin protein was reduced in potato^41^. However, a recent RNA-seq study of green algae *Chlamydomonas reinhardtii* found that transcription level of patatin lipid acylhydrolase was significantly up-regulated by heat stress^42^. In this study, the commonly-upregulated LOX and PAT2 by heat and drought suggested the important of those two genes for plant tolerance to both stresses, although the underlying mechanisms deserve further investigation.

The transcript level of delta-1-pyrroline-5-carboxylate synthase (P5CS) in proline biosynthetic process was also significantly up-regulated by drought and heat stress (Fig. 3, Table 1). Proline is well-known for its role as an osmolyte for osmotic adjustment, a nitrogen and carbon provider, a source of energy, a metal chelator and a signal molecule, and is also involved in stabilizing cell membranes and proteins, scavenging free radicals, balancing redox potential, as well as functioning as a protein-compatible hydrotrope, alleviating acidosis, and maintaining NADP+/NADPH ratios^43–46^. P5CS catalyzes the first two committing step of proline biosynthesis, reducing glutamate to glutamate-semialdehyde^47^. P5CS protein is encoded by two genes, P5CS1 and P5CS2, in which P5CS1 could be induced by drought and salt stresses, and P5CS2 is apparently a housekeeping gene for basic proline metabolism^48–51^. The available literature reporting the effects of heat stress on P5CS transcript levels or enzymatic activities varied with the severity or duration of heat stress. P5CS enzyme activity in sorghum (*Sorghum bicolor*) seedlings was found to be induced upon short-term heat stress (6 h at 40 °C)^52^, but inhibited under long-term heat stress, such as in wheat (5 d at 33 °C)^53^, and tomato (*Solanum lycopersicum*) (48 h at 35 °C)^54^. Similarly, transcript levels of P5CS were up-regulated by short-term heat stress in *Nitraria tangutorum* (6 h at 50 °C)^55^, but down-regulated by long- term heat stress in peach (*Prunus persica*) (5 d at 36.7 °C)^56^. The common up-regulation of proline synthesis by both long-term (21 d) heat and drought stress in this study suggested the importance of P5CS regulation of proline synthesis in *A. stolonifera* adaptation to prolonged heat and drought stress.

Among commonly down-regulated transcripts by heat and drought stress, thiamine thiazole synthase 2 (TTS2) and phosphomethylpyrimidine synthase (PMPS) are both involved in thiamine biosynthetic pathway (GO:0009228), thiamine-containing compound biosynthetic pathway (GO:0042724), and thiamin metabolic pathway (GO:0006772), which are highly enriched in GO term enrichment analysis (Fig. 5, Table 2). Thiamine (Vitamin B1) in plants plays a fundamental role as an enzymatic cofactor in glycolysis, pentose phosphate pathway, and tricarboxylic acid cycle and is involved in amino acid and nonmevalonate isoprenoid biosynthesis^57^. It was recently reported to regulate cellular tolerance to DNA damages and serves as activators for pathogen attacks in plants^58,59^, whereas its roles in abiotic stress tolerance are not well documented. The expression of thiamine thiazole synthase (THI1/THI4), and phosphomethylpyrimidine synthase (THIC) gene, was transiently induced by osmotic stress, but the transient induction could be alleviated, or even reversed, by increasing stress duration or severity^60–62^. The THI1 protein abundance and mRNA level were also reported to be increased under heat stress in several plant species^63–65^. The transient elevation of such protein abundances and transcript levels in response to stresses could be possibly due to its involvement in DNA protection or repair^64^. The down-regulation of TTS2 and PMPS involved in thiamin synthesis in *A. stolonifera* under drought and heat stress indicated the significance of thiamin in regulating plant responses to both heat and drought stress in perennial grass species.

Drought and heat stresses caused significant down-regulation of calcium sensing receptor (CSR), which is involved in cellular component of chloroplast and photosystem in GO term enrichment analysis (Fig. 6, Table 2). Calcium sensing receptor is a chloroplast protein localized in the thylakoid membrane, bearing a calcium-binding acidic N-terminal and a rhodanese domain C-terminal^66,67^. Previous studies have shown that calcium sensing receptor positively regulates stomatal closure and photosynthetic electron transport, which is important for drought avoidance^68–70^. However, previous literatures of plant calcium sensing upon heat stress mainly focused on calcium channels and calmodulins, with little information about CSR^71–74^. The common down-regulation of CSR in *A. stolonifera* by drought and heat stress suggested the suppression of calcium sensing involving CSR for perennial grass responses to heat and drought stress, although further studies needed to explore the underlying mechanisms for CSR as a negative regulator for drought and heat responses.

## Materials and Methods

### Plant materials and growth conditions

Tillers of *A. stolonifera* (‘Penncross’) were collected from stock plants and transferred to plastic containers (57 × 44 × 30 cm, 12 drainage holes) filled with fritted clay medium (Profile Products, Deerfield, IL). Each container was planted with 30 tillers. Plants were established from tillers for 35 d in a greenhouse with average temperature of 23/20 °C (day/night), 60% relative humidity (RH), and 750 µmol m-2 s-1 photosynthetically active radiation (PAR) from natural sunlight and supplemental lighting. Plants were irrigated daily, fertilized twice per week with half-strength Hoagland’s nutrient solution^75^, and trimmed to the canopy height of 2 cm once per week during establishment. Plants were not trimmed during the final week of establishment to allow for sufficient regrowth prior to stress imposition, after which time all plants were transferred to controlled-environment growth chambers (Environmental Growth Chamber, Chagrin Falls, Ohio). Plants were maintained in controlled-environment growth chambers controlled at 22/18 °C (day/night), 600 µmol m-2 s-1 PAR, 60% RH, and 14-h photoperiod for one week prior to stress imposition.

### Stress treatments and experimental design

For heat stress, air temperature was controlled at 35/30 °C (day/night) for 21 d. For drought stress, plants were withheld from irrigation for 21 d at 22/18 °C (day/night). For the non-stress control, plants were watered every other day and maintained at 22/18 °C (day/night). Each treatment was repeated in four containers containing multiple plants or had four biological replicates. The treatments were conducted in four growth chambers.

### Physiological measurements

Leaf relative water content (RWC) and electrolyte leakage (EL) were measured at 0, 7, 14 and 21 d of heat or drought stress to assess physiological responses. Approximately 0.8 g fresh leaf tissues were collected from four individual plants per container, and then pooled for RWC and EL measurements. For RWC, approximately 0.2 g of leaf blades were first weighed for fresh weight (FW), soaked in water for 12 h and again weighed for turgid weight (TW), dried in an oven at 80 °C for 3 d, and finally weighed for dry weight (DW). RWC was calculated using the formula (%) = ([FW − DW]/[TW − DW]) × 100^76^. For EL, approximately 0.2 g of fresh leaf tissue was rinsed with deionized water, placed in a test tube containing 30 mL deionized water, agitated on a conical shaker for 12 h, and initial conductance (Ci) measured using a conductivity meter (YSI Model 32, Yellow Springs, OH). Tubes containing leaf tissue were then autoclaved at 121 °C for 20 min and then agitated for 12 h. The maximal conductance (Cmax) of incubation solution was then measured and EL (%) was calculated as ((Ci/Cmax) × 100)^77^. Four biological replicates (n = 4) were performed for each parameter under either control or stress condition, respectively. Statistical differences between treatment means were separated by Student’s t-test at the P level of 0.05.

### RNA extraction, library preparation, and RNA sequencing

Total RNA was extracted from 200 mg of leaf samples collected at 21 d of stress when most significant physiological effects of heat or drought stress were present. The extraction was performed using TRIzol reagent (Life Technologies, Grand Island, NY), and treated with TURBO DNA-free kit (Life Technologies, Grand Island, NY). The quality and quantity of RNA was assessed in a NanoDrop 1000 spectrophotometer (Thermo Fisher Scientific, Waltham, MA). A total of 9 libraries (3 treatments × 3 biological replicates) were prepared for RNA-seq. Total RNA (2 μg) was used for construction of each library using the Illumina TruSeq RNA Library Prep Kit v2 (Illumina, San Diego, CA) according to the Low Sample (LS) protocol. LS protocol was amended to lower the Elute 2-Fragment-Prime 94 °C incubation time from 8 min to 1 min to generate larger RNA fragments. Indexes were chosen to allow for library multiplexing per run and libraries were pooled in an equimolar fashion. Pooled libraries were prepared for MiSeq run according to Illumina recommendations and loaded into a 600-cycle MiSeq Reagent Kit v3 cartridge (Illumina, San Diego, CA) at a concentration of 20 pM. Each run was set as pair-end (PE) 2 × 300 bp, fastq format only, and no adapter trimming.

### *De novo* assembly, read quantification, gene expression differentiation and functional analysis

Raw reads from MiSeq sequencing were downloaded and analyzed using samtools command flagstat^78^. Reads were then assembled using Trinity^79^, with quality trimming using Trimmomatic option. The parameters were set as follows: “Trinity–max_memory 64G, –CPU 8, –bflyCPU 2, –bflyHeapSpaceMax 64G, –trimmomatic ILLUMINACLIP::2:30:15:8:TRUE SLIDINGWINDOW:4:20 LEADING:20 TRAILING:20 MINLEN:60 HEADCROP:6 CROP: 275”. Transcripts obtained were clustered using CDHITEST^80^, with the following parameters: “cd-hit-est -c 0.9, -n 8”. The transcripts were then quantified using RSEM^81^, which was incorporated as the “align_and_estimate_abundance.pl” script in Trinity program, using default parameters. Differential expression analysis of transcripts were performed using edgeR^82^, which was also nested in the “run_DE_analysis.pl” script in Trinity, using default parameters. The ratios of transcript abundances under stress conditions to control condition for each species were filtered with threshold of false discovery rate (FDR) < 0.001, in order to get differentially expressed genes (DEGs). In addition, the coding regions of transcript assemblies were identified using TransDecoder, and then annotated using Trinotate^79^.

Gene ontology (GO) term classification was performed by CateGOrizer^83^, using “GO_slim2” method. The GO enrichment analysis for DEGs was performed using GOEAST^84^, by implementing Customized Result Analysis for up- and down-regulated DEGs, respectively.

The transcriptome shotgun assembly of *A. stolonifera* were deposited at GenBank Transcriptome Shotgun Assembly (TSA) database, under the accession of GFJH00000000. The version described in this paper is the first version, GFJH01000000.

### Validation of gene expression levels

Gene expression levels were also measured by quantitative reverse transcriptase-polymerase chain reaction (qRT-PCR). Total RNA was isolated from ground leaf powder using TRIzol reagent (Life Technologies, Grand Island, NY) and treated with DNase (TURBO DNA-free kit; Life Technologies, Grand Island, NY) in order to remove contaminating genomic DNA. Total RNA (2 μg) was reverse-transcribed using a high-capacity cDNA reverse transcription kit (Life Technologies, Grand Island, NY). The synthesized cDNA was amplified in a StepOnePlus Real-Time PCR system (Life Technologies, Grand Island, NY) using the following parameters: pre-heat cycle of 95 °C for 3 min, 40 cycles of 95 °C denaturation for 30 sec per cycle, and 60 °C annealing/extension for 30 sec per cycle. Power SYBR Green PCR Master Mix (Life Technologies, Grand Island, NY) was the intercalating dye used to detect gene expression level. Gene name, accession number, forward and reverse primer sequences are provided in Table 4. A melting curve analysis was performed for each primer set to confirm its specificity. Actin was used as the reference gene, since its expression was consistent throughout treatments. A ΔΔCt method was used to calculate the relative expression level between genes of interest and reference gene, respectively^85^. Four biological replicates (n = 4) were performed for each gene under either control or stress conditions, respectively. Statistical differences between treatment means were separated by Student’s t-test at the P level of 0.05.

**Table 4:** Primer sequences of genes used in qRT-PCR. Gene names and transcript IDs are also listed.

Gene	ID	Primer sequence	
**Up-regulation**			
*Linoleate 9S-lipoxygenase 1 (LOX1)*	TRINITY_DN120383_c6_g1	Forward	GAGCATCATTGGAGTGTCTG
		Reverse	CCTTCTTTGCCTTGTCATCT
*Patatin-like protein 2 (PLP2)*	TRINITY_DN103758_c0_g1	Forward	GCTTGTTTCGGTCCTACTAC
		Reverse	GCCAGGTTCCAGAAGAAAG
*Delta-1-pyrroline-5-carboxylate synthase (P5CS)*	TRINITY_DN114921_c0_g21	Forward	GGAGGACCCTATTTCCCATA
		Reverse	GCATCAGGACGAGATTCAAA
*9-cis-epoxycarotenoid dioxygenase 1 (NCED1)*	TRINITY_DN119819_c3_g2	Forward	CCTCTCTCCCTATCCCTAATC
		Reverse	CACTAGTTGGTTTCTGTCCATA
*3-ketoacyl-CoA synthase 11 (KCS11)*	TRINITY_DN100275_c1_g2	Forward	GGCCTAAATGGAATCGTCTAA
		Reverse	TTGCGGTCGACTGAATAAC
**Down-regulation**			
*Thiamine thiazole synthase 2 (TTS2)*	TRINITY_DN113085_c9_g20	Forward	GCCTATGAACAAGACGCTAAAC
		Reverse	AACGAGGTCCTTCTCCTTTG
*Phosphomethylpyrimidine synthase (PMPS)*	TRINITY_DN116433_c3_g10	Forward	GATTGGACTCCCGAAGATAAG
		Reverse	GTACAGCATCTCCTCTGTTATG
*Chloroplast stem-loop binding protein of 41* *kDa b (CSLB41B)*	TRINITY_DN109357_c3_g19	Forward	TCTCCAGCTTTCCCTTGT
		Reverse	CGAACCTGGAACAGTTCATC
*Calcium sensing receptor (CSR)*	TRINITY_DN99853_c0_g1	Forward	CCGACATTTGCGGTGTT
		Reverse	CTGAAGTTCCAACTCTTGTTCT
*NAD(P)H-quinone oxidoreductase subunit 5 (NQO5)*	TRINITY_DN94571_c0_g1	Forward	CAGCCCAAGCGTCTTAAT
		Reverse	ACTGAAACGATTGCCATTATTC
*Oxygen-evolving enhancer protein 1 (OEE1)*	TRINITY_DN98821_c7_g1	Forward	GCCGCGCATAGATAACA
		Reverse	CCATGTCTGTGCCACTT
*Peptidyl-prolyl cis-trans isomerase CYP37*	TRINITY_DN103314_c0_g1	Forward	GGACGGAGGGAGTACTATTTA
		Reverse	GCACTTGTGCCCTGTATAA
*Photosynthetic NDH subunit of luminal location 1 (PNL1)*	TRINITY_DN92658_c0_g1	Forward	CAGTCCAGCAGCAAGATAG
		Reverse	CCATTCAACCTGCCAGTAA
*Photosystem I reaction center subunit IV (PI4)*	TRINITY_DN96282_c3_g3	Forward	CGATCGAGAAACTATCGAACTC
		Reverse	CTTACGGACAGACACCTTTG
*Photosystem II core complex protein psbY (PSBY)*	TRINITY_DN108240_c2_g3	Forward	AGGAACAAGATTAGAGAGTATGC
		Reverse	TCCTCGTCGTTGTAGTGT
*Protein CURVATURE THYLAKOID 1* *A (CUR1A)*	TRINITY_DN86325_c0_g2	Forward	ACGCTGAACAACAAGTAGG
		Reverse	CTGGTACAGAGGACGAGTAA
*Protein LOW PSII ACCUMULATION 1 (LOW1)*	TRINITY_DN106211_c1_g1	Forward	AGAGAGAGAGAGAGAGAGAGA
		Reverse	GGCAAGAAGTGATGATGATAGA
*PsbP domain-containing protein 3 (PSBP3)*	TRINITY_DN95645_c0_g2	Forward	GCGTCCTCCTCTCTTCT
		Reverse	AACTTGTTGGCCTCATCC
*Rhodanese-like domain-containing protein 4 (RHO4)*	TRINITY_DN108472_c0_g2	Forward	AGAATGTCCTCTCCTCTAGATAC
		Reverse	TTTCTCCATATGCGAACTATCC
*ACTIN*	Internal reference	Forward	CCTTTTCCAGCCATCTTTCA
		Reverse	GAGGTCCTTCCTGATATCCA

## Conclusions

**Figure 8:**
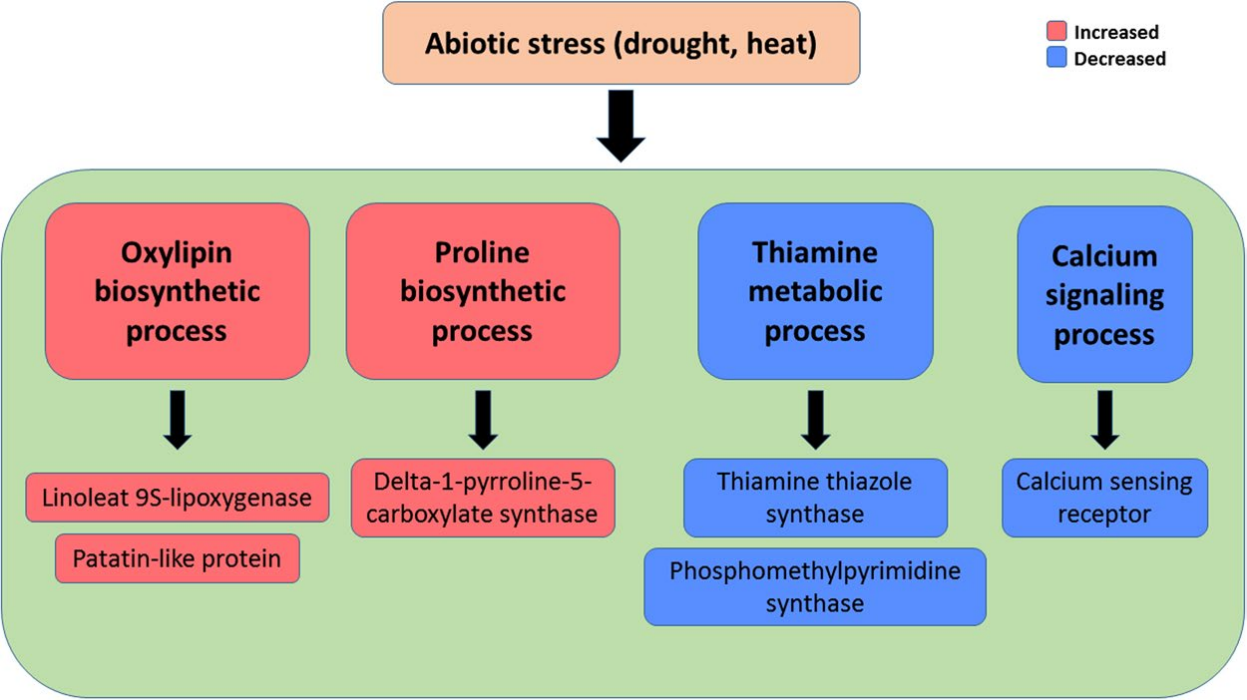
A comprehensive overview of transcriptional regulations under drought and heat stress in *A. stolonifera*.

In summary, comparative transcriptomic analysis of *A. stolonifera* between drought and heat stress found 670 up-regulated and 812 down-regulated DEGs. Transcriptional regulations of DEGs that are responsive to both heat and drought stresses include up-regulation of genes in oxylipin biosynthetic process, proline biosynthetic process; and down-regulations of genes in thiamine metabolic process and calcium sensing receptor, which is summarized in Fig. 8. These commonly regulated genes identified in *A. stolonifera* could be potential candidate genes for genetic modification of cultivated grass species for improving both heat and drought tolerance, although the direct relationship of those genes and the associated pathways contributing to plant tolerance to both heat and drought tolerance requires further investigation.

## Electronic supplementary material

supplemental table 1

supplemental table 2

supplemental table 3
