# Supplementary material for: Comparative transcriptomic analysis reveals common molecular factors responsive to heat and drought stress in Agrostis stolonifera
Source: Sci Rep. 2018 Oct 12;8:15181. doi: 10.1038/s41598-018-33597-3 (PMC6185948; doi:10.1038/s41598-018-33597-3)
Supplement: Supplementary file 1 — supplemental table 1 [file 41598_2018_33597_MOESM1_ESM.pdf]

Comparative transcriptomic analysis reveals common molecular factors responsive to heat and drought st

Yi Xu and Bingru Huang

Transcript ID	Description	Drought stress		Heat stress	
		LogFC	FDR	LogFC	FDR
TRINITY_DN106580_c0_g2	PXG5_ARATH^	9.618507	1.70E-13	5.964314	4.49E-06
TRINITY_DN91271_c3_g3	PXG5_ARATH^	9.391785	1.22E-13	5.999979	3.81E-05
TRINITY_DN99323_c0_g3	#N/A	8.839027	1.87E-23	6.96623	2E-08
TRINITY_DN103642_c3_g1	BH148_ORYSJ^	8.816736	8.98E-14	9.024869	2.45E-25
TRINITY_DN114378_c0_g1	PUB19_ARATH	8.711422	2.55E-22	7.583992	3.38E-14
TRINITY_DN23070_c0_g1	#N/A	8.21538	1.26E-14	11.0625	7.63E-78
TRINITY_DN101487_c5_g1	#N/A	8.163572	6.24E-09	10.77945	4.21E-72
TRINITY_DN7571_c0_g1	#N/A	8.115604	1.94E-17	9.093015	2.42E-25
TRINITY_DN88461_c0_g1	LECH_HORVU^	8.111145	2.23E-10	10.70975	1.49E-30
TRINITY_DN23070_c1_g1	#N/A	8.100998	6.67E-14	10.75813	1.88E-67
TRINITY_DN96306_c0_g2	OP162_ARATH	7.87572	5.04E-10	9.227917	7.38E-40
TRINITY_DN110784_c0_g2	LEA34_GOSHI^	7.874001	3.56E-10	10.01703	8.66E-42
TRINITY_DN98159_c7_g54	DHN3_HORVU	7.849131	1.99E-22	9.946002	1E-117
TRINITY_DN111576_c1_g1	#N/A	7.81565	7.38E-10	9.532235	4.84E-45
TRINITY_DN108395_c0_g1	P2C29_ORYSJ^	7.704963	6.21E-09	6.333231	1.02E-05
TRINITY_DN113158_c0_g2	SWT15_ORYSJ	7.629741	6.73E-10	8.412183	3.44E-19
TRINITY_DN98298_c0_g1	TI11E_ORYSJ^T	7.582243	4.58E-08	7.69206	5.89E-14
TRINITY_DN85625_c0_g2	BSMT1_ARATH	7.460466	2.57E-11	5.720735	9.1E-05
TRINITY_DN114562_c0_g2	MAOX_VITVI^I	7.424349	9.42E-07	10.46204	1.12E-56
TRINITY_DN109304_c0_g2	PMA1_SOLLC^	7.413381	5.89E-08	9.016689	5.52E-24
TRINITY_DN101487_c5_g12	DHN3_HORVU	7.379535	1.01E-19	9.539298	4.4E-117
TRINITY_DN86288_c0_g3	DHN1_HORVU	7.327539	1.21E-05	10.88274	3.72E-55
TRINITY_DN230056_c0_g1	#N/A	7.31805	1.85E-11	6.076408	5.02E-05
TRINITY_DN114834_c4_g9	NAMB2_TRITD	7.312825	8.11E-09	7.190565	2.71E-12
TRINITY_DN112237_c0_g7	EF109_ARATH	7.309626	3.59E-07	5.716011	9.39E-05
TRINITY_DN160106_c0_g1	#N/A	7.266053	5.19E-11	6.586201	8.09E-06
TRINITY_DN83548_c0_g2	#N/A	7.214064	1.74E-08	7.321857	3.42E-13
TRINITY_DN35178_c1_g2	#N/A	7.211334	4.74E-38	7.502545	4.68E-35
TRINITY_DN114165_c0_g1	PUB19_ARATH	7.18379	4.32E-19	5.910016	1.63E-17
TRINITY_DN180285_c0_g1	#N/A	7.157173	1.84E-09	6.501091	1.26E-07
TRINITY_DN106495_c0_g1	HSA32_ARATH	7.044136	1.23E-08	6.27056	1.03E-07
TRINITY_DN96770_c0_g1	EF109_ARATH	6.992888	1.56E-06	6.909959	4.06E-09
TRINITY_DN95299_c0_g2	TI11E_ORYSJ^T	6.980685	1.79E-05	6.984372	6.68E-11
TRINITY_DN89675_c0_g5	#N/A	6.921184	6.99E-05	5.754708	0.000132
TRINITY_DN106216_c1_g2	DHN2_HORVU	6.917697	2.61E-05	10.20538	7.35E-44
TRINITY_DN115526_c0_g2	AGT23_ARATH	6.914914	3.18E-05	9.358882	9.23E-33
TRINITY_DN90942_c0_g9	#N/A	6.903575	2.27E-06	5.90407	0.000114
TRINITY_DN95545_c0_g1	TI11E_ORYSJ^T	6.864807	3.65E-05	6.090835	0.000124
TRINITY_DN162162_c0_g1	#N/A	6.817191	6.62E-24	6.78931	3.25E-27
TRINITY_DN109753_c0_g1	ADHL6_ARATH	6.811664	1.40E-07	5.648364	5.51E-05
TRINITY_DN116739_c2_g6	#N/A	6.777564	1.60E-05	8.483098	1.54E-26

TRINITY_DN97039_c1_g2	#N/A	6.770979	5.20E-06	7.3879	5.2E-12
TRINITY_DN86707_c0_g1	NLTP4_ORYSJ^	6.7397	8.11E-06	5.416014	0.000498
TRINITY_DN112124_c7_g15	NAMB2_TRITD	6.733806	1.02E-06	7.012628	1.14E-12
TRINITY_DN92661_c1_g2	OP162_ARATH	6.71136	2.71E-06	7.413514	8.66E-15
TRINITY_DN89723_c0_g2	#N/A	6.68464	8.08E-08	7.635694	2.27E-16
TRINITY_DN110695_c5_g6	HS174_ORYSJ^	6.675297	2.49E-07	7.249588	1.13E-09
TRINITY_DN93067_c0_g1	#N/A	6.597142	1.27E-07	6.30203	4.51E-07
TRINITY_DN117627_c1_g8	#N/A	6.56233	1.07E-05	5.422517	0.000497
TRINITY_DN106495_c1_g4	HSA32_ARATH	6.507643	7.86E-08	6.567374	1.19E-08
TRINITY_DN142001_c0_g1	#N/A	6.487162	9.70E-07	5.416015	0.000498
TRINITY_DN88714_c0_g6	#N/A	6.458201	1.74E-17	5.647293	6.85E-23
TRINITY_DN99824_c1_g4	SHH2_ARATH^	6.4334	1.24E-05	5.844245	5.1E-05
TRINITY_DN112109_c0_g1	#N/A	6.426014	9.46E-05	5.575725	0.000131
TRINITY_DN73633_c0_g1	#N/A	6.420059	4.43E-05	9.594127	2.05E-25
TRINITY_DN88847_c0_g1	TET8_ARATH^	6.4112	4.21E-05	6.110833	5.57E-05
TRINITY_DN47245_c0_g2	#N/A	6.393463	1.98E-30	6.712363	2.58E-31
TRINITY_DN118124_c0_g1	#N/A	6.362532	0.000425	6.247268	1.07E-06
TRINITY_DN99843_c1_g3	HVA22_HORVU	6.359417	7.92E-07	6.70038	4.16E-09
TRINITY_DN106216_c1_g1	DHN1_MAIZE^	6.348119	0.000239	9.152047	3.96E-37
TRINITY_DN118998_c1_g1	PMA2_SOLLC^	6.332156	8.00E-06	7.487376	7.64E-15
TRINITY_DN85342_c0_g1	OP162_ARATH	6.31221	7.31E-05	8.14577	2.36E-20
TRINITY_DN93832_c0_g1	EF109_ARATH^	6.289187	0.000267	7.240173	9.68E-13
TRINITY_DN117722_c2_g27	#N/A	6.267124	1.10E-05	7.957022	4.5E-20
TRINITY_DN114834_c4_g8	NAMB2_TRITD	6.242082	8.06E-06	6.087343	2.77E-06
TRINITY_DN61432_c1_g1	DHN3_HORVU	6.239735	0.000396	7.416612	6.6E-15
TRINITY_DN92581_c0_g2	#N/A	6.236952	1.80E-05	6.71061	5.2E-07
TRINITY_DN104354_c0_g14	LEA3_ORYSJ^L	6.193033	0.000762	10.29257	9.56E-56
TRINITY_DN93252_c0_g2	#N/A	6.190826	0.000222	6.673205	4.65E-06
TRINITY_DN113369_c1_g3	FL3H_HORVU^	6.167721	0.000328	7.035581	4.73E-13
TRINITY_DN105884_c0_g2	MNR1_CAPAN	6.132933	1.22E-05	5.870149	0.000142
TRINITY_DN103215_c0_g2	CLPS_GLOVI^C	6.130647	7.41E-05	6.925506	4.72E-10
TRINITY_DN118123_c0_g4	MSL4_ARATH^	6.125289	4.10E-06	7.515426	6.48E-14
TRINITY_DN81892_c0_g4	HIP26_ARATH^	6.092168	3.18E-05	6.627877	2.4E-07
TRINITY_DN26217_c0_g2	#N/A	6.066604	1.08E-05	5.499467	0.000192
TRINITY_DN111531_c0_g1	E135_ARATH^I	6.060181	0.00017	8.663121	1.91E-26
TRINITY_DN91093_c0_g1	LECH_HORVU^	6.050482	8.06E-09	8.768396	9.82E-27
TRINITY_DN103927_c0_g6	#N/A	6.044717	1.47E-10	4.925441	2.64E-09
TRINITY_DN96955_c0_g4	#N/A	6.041295	0.000207	8.413683	2.23E-21
TRINITY_DN96055_c1_g2	#N/A	6.029276	1.25E-05	6.315603	5.37E-08
TRINITY_DN117722_c2_g3	#N/A	6.022543	0.000285	7.763958	2.58E-17
TRINITY_DN113417_c3_g19	#N/A	6.009189	0.000468	8.286289	2.24E-21
TRINITY_DN88864_c3_g1	GL84_ORYSJ^G	6.005623	2.70E-12	5.315516	2.34E-26
TRINITY_DN115665_c0_g2	C7091_ARATH	6.005051	7.33E-14	7.045919	1.92E-24
TRINITY_DN76434_c0_g2	#N/A	6.002678	0.000376	8.845939	3.6E-20
TRINITY_DN96104_c0_g1	TET8_ARATH^	5.994473	0.000122	6.433361	5.88E-08
TRINITY_DN92480_c0_g1	PM1_SOYBN^F	5.990143	1.77E-13	6.955505	3.45E-44
TRINITY_DN108373_c3_g5	MKKA_DICDI^I	5.94145	1.73E-10	5.400309	2.11E-09

TRINITY_DN109055_c0_g3	MKKA_DICDI^I	5.938991	2.86E-12	5.258004	6.49E-10
TRINITY_DN112863_c0_g3	#N/A	5.913167	1.78E-05	5.917514	1.09E-05
TRINITY_DN82902_c0_g2	LEA3_WHEAT^	5.912125	0.000535	9.783443	3.17E-39
TRINITY_DN98159_c7_g41	#N/A	5.895605	0.000803	8.543784	1.3E-22
TRINITY_DN102174_c0_g1	Y5707_ARATH	5.895599	0.000102	6.224162	3.11E-07
TRINITY_DN49599_c0_g2	LOX3_ORYSJ^L	5.893784	1.44E-13	4.580824	2.16E-06
TRINITY_DN99663_c1_g2	#N/A	5.84716	0.000101	6.789772	3.93E-08
TRINITY_DN112807_c0_g1	YJNA_SCHPO^	5.843537	0.000188	8.106806	5.9E-18
TRINITY_DN117627_c1_g19	#N/A	5.818134	0.000345	5.647043	0.000134
TRINITY_DN110198_c0_g2	NUD19_ARATH	5.811348	0.000446	7.013154	2.14E-11
TRINITY_DN85196_c0_g2	#N/A	5.799766	8.91E-05	6.756991	1.2E-08
TRINITY_DN88000_c0_g2	#N/A	5.789276	0.000712	7.178752	3.42E-13
TRINITY_DN96618_c0_g3	TSJT1_TOBAC^	5.777208	0.000521	6.559437	4.66E-07
TRINITY_DN159691_c0_g1	#N/A	5.7423	1.01E-19	3.454826	3.99E-06
TRINITY_DN105396_c0_g3	ZAT10_ARATH	5.73674	0.000419	6.377179	3.8E-07
TRINITY_DN96913_c0_g1	#N/A	5.735622	0.000158	8.002252	1E-19
TRINITY_DN104594_c0_g2	ATL41_ARATH	5.734172	0.000196	6.486242	3.59E-06
TRINITY_DN115056_c0_g5	C89A2_ARATH	5.721968	1.87E-11	4.107195	1.85E-05
TRINITY_DN116739_c2_g14	#N/A	5.708093	0.00088	7.468614	1.32E-13
TRINITY_DN105192_c1_g4	#N/A	5.701062	1.32E-10	4.306028	2.14E-05
TRINITY_DN114890_c11_g8	RFS2_ARATH^I	5.662704	0.000181	6.772752	2.32E-08
TRINITY_DN160967_c0_g1	#N/A	5.584177	2.02E-17	5.312079	8.18E-18
TRINITY_DN86242_c1_g1	GL84_ORYSJ^G	5.555005	7.05E-12	5.222795	5.91E-23
TRINITY_DN92661_c1_g3	OP162_ARATH	5.549084	2.08E-05	6.624661	2.73E-26
TRINITY_DN114077_c0_g2	ASO_CUCPM^/	5.476966	3.87E-20	4.294708	7.72E-22
TRINITY_DN96274_c2_g1	#N/A	5.361078	5.90E-11	5.297746	1.81E-09
TRINITY_DN112841_c0_g1	SWT15_ORYSJ	5.358518	7.05E-06	5.523128	6.85E-12
TRINITY_DN78117_c2_g3	LOX3_ORYSJ^L	5.3463	3.01E-09	4.323574	3.09E-05
TRINITY_DN106507_c0_g1	EGY3_ORYSJ^E	5.322557	4.71E-15	4.198375	2.14E-11
TRINITY_DN102105_c0_g1	CSPLP_ORYSJ^	5.277515	5.05E-13	3.788787	2.86E-06
TRINITY_DN101373_c2_g3	GRXS5_ORYSJ^	5.268641	1.96E-07	5.217937	4.85E-08
TRINITY_DN108659_c0_g1	PMA1_SOLLC^	5.227788	4.05E-07	7.13599	4.43E-21
TRINITY_DN30465_c0_g1	#N/A	5.207367	3.33E-13	4.375809	1.3E-07
TRINITY_DN92010_c0_g1	PM1_SOYBN^F	5.191541	4.77E-13	5.991887	2.76E-40
TRINITY_DN112941_c1_g2	E70B1_ARATH	5.158885	2.40E-05	5.448864	1.11E-13
TRINITY_DN117627_c1_g24	#N/A	5.155312	1.74E-07	5.511809	1.86E-20
TRINITY_DN113417_c3_g20	#N/A	5.137831	1.81E-06	6.975028	1.8E-26
TRINITY_DN101413_c0_g1	#N/A	5.113186	8.35E-08	4.741712	1.75E-07
TRINITY_DN77778_c2_g1	IBB_HORVU^IE	5.098587	2.41E-41	4.594866	1.07E-56
TRINITY_DN116945_c0_g1	MAOP4_ARATI	5.08419	0.000171	8.595613	6.02E-52
TRINITY_DN59097_c1_g1	IBB_HORVU^IE	5.048672	4.62E-22	4.50075	1.32E-22
TRINITY_DN113417_c3_g14	#N/A	5.030324	2.85E-10	7.034515	3.81E-44
TRINITY_DN101234_c0_g20	#N/A	5.000368	6.35E-08	4.893818	4.56E-09
TRINITY_DN35555_c0_g1	#N/A	4.981062	1.12E-18	6.05942	6.4E-34
TRINITY_DN97732_c6_g10	ZAT12_ARATH	4.969455	0.000118	4.051426	2.45E-05
TRINITY_DN112124_c7_g2	NAC10_ORYSI^	4.95989	3.70E-08	4.887423	6.14E-08
TRINITY_DN118125_c0_g5	EOGT_BOVIN^	4.940633	2.29E-06	3.995853	4.43E-05

TRINITY_DN100645_c3_g8	#N/A	4.890137	4.31E-09	4.36725	5.35E-06
TRINITY_DN93473_c1_g1	K502_ACTDE^I	4.887242	2.88E-30	3.457374	2.59E-26
TRINITY_DN102545_c3_g7	HOX24_ORYSI^	4.883518	6.29E-07	4.357271	5.23E-05
TRINITY_DN111195_c0_g1	NRX2_ORYSJ^I	4.882819	3.89E-14	5.82266	1.32E-29
TRINITY_DN114131_c0_g1	ASO_CUCPM^/	4.85067	6.13E-25	3.539021	5.03E-14
TRINITY_DN119819_c3_g2	NCED1_MAIZE	4.843782	3.67E-06	5.74434	4.47E-14
TRINITY_DN121061_c0_g1	IBB_HORVU^IE	4.840401	1.17E-06	3.782327	4.27E-06
TRINITY_DN118124_c0_g21	#N/A	4.825732	0.000116	4.962465	2.3E-10
TRINITY_DN117722_c2_g13	#N/A	4.796897	1.45E-06	8.721675	5.25E-65
TRINITY_DN111169_c0_g12	SR45A_ARATH	4.778695	4.59E-05	4.899038	1.85E-08
TRINITY_DN75576_c0_g1	#N/A	4.767524	1.19E-12	4.850716	3.1E-13
TRINITY_DN120383_c6_g1	LOX1_HORVU^	4.754847	2.23E-05	3.192754	0.000904
TRINITY_DN73540_c0_g1	#N/A	4.75292	8.96E-05	6.199298	8.08E-34
TRINITY_DN121490_c0_g1	#N/A	4.68241	2.50E-05	5.166884	3.05E-07
TRINITY_DN89675_c0_g3	#N/A	4.677481	3.13E-05	4.235596	0.000144
TRINITY_DN140681_c0_g1	#N/A	4.66576	4.38E-07	5.029574	3.95E-07
TRINITY_DN178709_c0_g1	#N/A	4.66427	5.39E-05	4.672391	1.81E-05
TRINITY_DN113730_c1_g4	#N/A	4.652702	1.88E-05	5.541579	2.49E-12
TRINITY_DN119066_c0_g4	#N/A	4.622285	4.54E-05	4.687458	1.24E-07
TRINITY_DN85625_c0_g4	AAM1I_MAIZE	4.610386	9.75E-10	3.175019	0.000667
TRINITY_DN90586_c0_g2	#N/A	4.586259	7.39E-10	3.440918	0.000254
TRINITY_DN102635_c0_g4	#N/A	4.58487	4.77E-05	4.684919	7.84E-07
TRINITY_DN107514_c2_g10	#N/A	4.57627	4.75E-09	5.840952	1.87E-17
TRINITY_DN219317_c0_g1	#N/A	4.567876	2.84E-28	2.726302	8.39E-13
TRINITY_DN110784_c1_g1	LEA34_GOSHI^	4.565288	0.00071	7.180637	6.97E-26
TRINITY_DN100645_c3_g6	#N/A	4.560631	5.75E-08	3.824591	6.42E-09
TRINITY_DN220887_c0_g1	#N/A	4.559381	2.76E-34	3.532125	1.2E-25
TRINITY_DN113679_c4_g1	CCD_CROSA^C	4.559062	8.39E-25	2.710651	6.22E-12
TRINITY_DN91615_c0_g1	OP162_ARATH	4.549462	9.96E-06	6.155498	2.22E-19
TRINITY_DN201942_c0_g1	#N/A	4.547001	0.000105	4.642954	2.01E-06
TRINITY_DN107406_c0_g6	DNAJ8_ARATH	4.530527	0.00023	4.008437	0.000374
TRINITY_DN101413_c0_g3	#N/A	4.51285	4.85E-10	4.710906	5.8E-17
TRINITY_DN95133_c3_g1	COMT1_MAIZE	4.503009	2.88E-05	4.819254	3.26E-06
TRINITY_DN112017_c2_g6	TI11B_ORYSJ^	4.500783	6.79E-15	4.068289	7.73E-12
TRINITY_DN93667_c3_g1	K502_ACTDE^I	4.497146	7.36E-26	2.808027	4.21E-12
TRINITY_DN102545_c3_g2	HOX24_ORYSI^	4.473669	2.68E-06	4.528806	2.7E-09
TRINITY_DN86428_c0_g1	#N/A	4.470268	4.16E-08	5.670084	6.54E-25
TRINITY_DN120383_c7_g23	LOX2_ORYSJ^L	4.447945	3.28E-05	3.498859	8.31E-06
TRINITY_DN105454_c0_g2	DHAR2_ARATH	4.44332	1.52E-13	4.122651	1.85E-09
TRINITY_DN117918_c0_g6	#N/A	4.436224	8.11E-05	5.569658	1.01E-13
TRINITY_DN109906_c2_g3	WRK40_ARATH	4.429727	5.13E-14	3.468136	3.01E-07
TRINITY_DN102910_c0_g1	TDA6_YEAST^T	4.410782	2.19E-05	4.731962	5.96E-08
TRINITY_DN105894_c8_g8	CXE17_ARATH	4.409786	3.78E-08	2.781002	6.31E-07
TRINITY_DN110176_c0_g1	HS17A_ORYSJ^	4.391187	5.73E-08	4.134726	7.14E-06
TRINITY_DN114890_c11_g12	RFS2_ARATH^I	4.389305	2.08E-15	4.721722	3.72E-55
TRINITY_DN74069_c1_g1	#N/A	4.385103	2.07E-13	4.828994	9.15E-29
TRINITY_DN61416_c0_g1	#N/A	4.373398	0.000376	7.206176	9.4E-29

TRINITY_DN99527_c0_g9	#N/A	4.361782	0.000146	4.007414	0.000169
TRINITY_DN89115_c1_g1	#N/A	4.34248	2.40E-08	3.758028	0.00012
TRINITY_DN120383_c6_g2	LOX1_ORYSJ^L	4.332066	1.03E-09	2.882114	3.84E-09
TRINITY_DN109118_c0_g4	P2C50_ORYSJ^	4.331023	1.46E-05	3.861904	6.52E-07
TRINITY_DN81153_c0_g1	PMGI_RICCO^I	4.329559	0.000165	6.00762	7.56E-18
TRINITY_DN109362_c2_g4	P2C08_ORYSJ^	4.32936	3.57E-05	5.091282	4.4E-10
TRINITY_DN67312_c0_g1	#N/A	4.293474	2.29E-05	5.931222	3.22E-17
TRINITY_DN114601_c12_g3	RFS2_ARATH^I	4.293316	1.63E-07	4.58055	1.53E-08
TRINITY_DN199607_c0_g1	#N/A	4.278297	1.53E-17	3.645388	2.2E-14
TRINITY_DN117722_c2_g30	#N/A	4.274711	1.17E-13	6.083168	3.89E-71
TRINITY_DN117561_c0_g2	NCED1_MAIZE	4.266933	7.43E-05	4.829636	2.11E-07
TRINITY_DN112423_c0_g2	AIB_ARATH^AI	4.251314	1.40E-08	3.194425	7.1E-05
TRINITY_DN109571_c0_g1	#N/A	4.23998	2.92E-11	6.580371	7.41E-56
TRINITY_DN96872_c0_g1	#N/A	4.233472	4.23E-06	3.506761	1.74E-06
TRINITY_DN104523_c2_g1	XTH12_ARATH	4.21388	3.60E-05	6.137341	9.69E-19
TRINITY_DN219548_c0_g1	#N/A	4.206973	1.16E-15	3.904041	3.45E-16
TRINITY_DN221452_c0_g1	#N/A	4.199071	5.01E-09	3.623282	2.79E-06
TRINITY_DN105401_c1_g1	HOX24_ORYSI^	4.189623	2.60E-18	4.531374	4.38E-22
TRINITY_DN109906_c2_g2	#N/A	4.187889	4.64E-09	3.450536	6.18E-07
TRINITY_DN101469_c0_g1	#N/A	4.179027	0.000701	4.904053	2.07E-07
TRINITY_DN75919_c0_g4	#N/A	4.173596	2.79E-09	4.673967	1E-15
TRINITY_DN106024_c0_g3	ERF1Y_ARATH^	4.172651	3.91E-19	6.221138	6.01E-85
TRINITY_DN102966_c0_g4	#N/A	4.168963	1.35E-10	5.144175	2.01E-15
TRINITY_DN105925_c0_g1	#N/A	4.164324	0.000523	5.535683	2.25E-18
TRINITY_DN83548_c1_g2	#N/A	4.153702	2.53E-05	4.173145	0.000108
TRINITY_DN101738_c0_g1	HSPR1_ARATH	4.151201	7.47E-16	2.916209	4.4E-06
TRINITY_DN99620_c1_g2	BAM1_ARATH^	4.151023	0.000251	6.005706	1.23E-18
TRINITY_DN110077_c0_g2	P2C50_ORYSJ^	4.140613	4.80E-06	3.343401	0.000222
TRINITY_DN58992_c0_g2	#N/A	4.138994	0.000585	5.313343	2.81E-11
TRINITY_DN86288_c0_g1	DHN1_HORVU	4.128008	0.000498	7.514935	4.7E-54
TRINITY_DN117630_c2_g10	PAO2_ARATH^	4.127711	9.70E-08	3.955495	2.04E-05
TRINITY_DN114601_c12_g8	RFS2_ARATH^I	4.127189	3.04E-06	4.605383	2.09E-11
TRINITY_DN106867_c3_g2	CXE15_ARATH	4.121621	6.19E-08	2.25115	0.000206
TRINITY_DN101814_c0_g1	PDR16_YEAST^	4.119723	6.51E-05	3.138431	0.000855
TRINITY_DN97200_c0_g1	LDOX_MAIZE^	4.114326	0.000455	4.399344	1.12E-05
TRINITY_DN198489_c0_g1	#N/A	4.112663	1.34E-32	3.131959	5.74E-22
TRINITY_DN112124_c7_g12	NAMB2_TRITD	4.106783	2.71E-07	4.116107	3.7E-09
TRINITY_DN87218_c0_g5	#N/A	4.079253	7.75E-05	3.513234	0.000684
TRINITY_DN88739_c0_g1	NLTP4_ORYSJ^	4.071716	1.11E-07	3.25966	8.45E-05
TRINITY_DN70764_c1_g1	#N/A	4.063897	5.86E-10	3.999836	3.88E-09
TRINITY_DN108707_c0_g2	NIPA3_ARATH^	4.060417	0.000188	4.159708	1.92E-05
TRINITY_DN120373_c0_g1	#N/A	4.052585	2.08E-08	4.400687	2.52E-10
TRINITY_DN64630_c0_g1	#N/A	4.04686	0.000523	5.470499	6.07E-31
TRINITY_DN113450_c0_g2	P2C29_ORYSJ^	4.045809	2.07E-06	3.064235	0.000224
TRINITY_DN120509_c1_g1	#N/A	4.039124	1.06E-23	2.557187	5.48E-16
TRINITY_DN98818_c0_g1	#N/A	4.034547	0.000502	5.007613	1.26E-10
TRINITY_DN113679_c9_g1	#N/A	4.028162	1.41E-06	4.136016	1.66E-06

TRINITY_DN102792_c2_g2	#N/A	4.00151	1.69E-07	3.118655	1.46E-05
TRINITY_DN93895_c0_g1	BAM1_ARATH^	3.997054	0.000616	6.992615	6.84E-28
TRINITY_DN109215_c0_g4	#N/A	3.992255	6.08E-06	3.484988	0.000455
TRINITY_DN101234_c0_g16	NAMB2_TRITD	3.991844	1.86E-09	3.87504	1.69E-13
TRINITY_DN114601_c12_g6	RFS2_ARATH^I	3.991046	6.01E-06	4.628241	2.7E-10
TRINITY_DN101390_c0_g6	#N/A	3.974901	2.38E-08	3.121675	9.62E-05
TRINITY_DN76472_c3_g1	#N/A	3.9495	4.79E-16	2.593431	2.35E-09
TRINITY_DN110043_c1_g2	XTH18_ARATH	3.945093	0.00025	5.662492	1.55E-15
TRINITY_DN91400_c2_g1	#N/A	3.943647	2.12E-11	3.598211	3.58E-19
TRINITY_DN88604_c2_g3	CCD_CROSA^C	3.941013	2.06E-22	1.724675	7.88E-06
TRINITY_DN104594_c0_g1	ATL41_ARATH^	3.931072	2.29E-05	4.2286	1.76E-09
TRINITY_DN110787_c1_g2	ENL2_ARATH^	3.926389	2.89E-08	5.502842	9.26E-23
TRINITY_DN117641_c1_g6	XIP1_WHEAT^	3.919092	1.82E-10	3.128815	5.47E-07
TRINITY_DN102031_c1_g2	#N/A	3.905293	2.91E-08	4.064063	8.17E-11
TRINITY_DN113495_c9_g13	NAC48_ORYSJ^	3.898261	0.00095	4.052663	4.08E-05
TRINITY_DN88031_c0_g1	#N/A	3.897418	0.000194	5.723382	3.25E-29
TRINITY_DN101649_c0_g1	P5CR_PEA^P5(	3.880936	3.08E-06	4.400842	1.6E-08
TRINITY_DN49074_c1_g2	#N/A	3.871497	2.81E-30	1.902575	8.16E-17
TRINITY_DN112930_c0_g4	#N/A	3.869351	7.34E-11	4.061123	9.06E-17
TRINITY_DN106801_c0_g5	ERF1Y_ARATH^	3.852747	3.89E-14	6.210177	1.77E-79
TRINITY_DN116784_c3_g2	MLO1_ARATH^	3.849173	1.74E-05	4.233371	2.73E-09
TRINITY_DN91645_c0_g10	#N/A	3.843989	1.17E-05	3.071104	5.12E-05
TRINITY_DN105559_c2_g7	#N/A	3.842299	5.48E-07	2.928357	0.00033
TRINITY_DN104396_c0_g1	#N/A	3.828359	1.17E-05	3.135716	0.000465
TRINITY_DN112017_c2_g9	TI11B_ORYSJ^	3.821434	1.41E-08	3.547445	1.98E-12
TRINITY_DN97538_c4_g2	#N/A	3.801021	1.26E-25	1.984013	1.1E-12
TRINITY_DN37180_c0_g2	#N/A	3.798675	2.53E-18	3.41591	4.6E-17
TRINITY_DN37180_c2_g1	#N/A	3.792588	4.74E-28	3.047384	2.24E-20
TRINITY_DN25354_c0_g1	#N/A	3.791349	1.96E-09	3.582387	4.56E-06
TRINITY_DN74223_c3_g1	#N/A	3.791123	1.35E-15	2.143677	5.67E-05
TRINITY_DN114601_c12_g7	RFS2_ARATH^I	3.790747	1.30E-19	4.317689	2.16E-68
TRINITY_DN110504_c0_g6	#N/A	3.782035	0.000964	5.765326	2.31E-15
TRINITY_DN49333_c0_g1	#N/A	3.779796	2.36E-31	2.718668	5.35E-16
TRINITY_DN115343_c0_g1	BAC2_ARATH^	3.775115	3.03E-07	4.531417	1.62E-14
TRINITY_DN92176_c0_g2	#N/A	3.774229	3.77E-07	5.748978	7.5E-25
TRINITY_DN110753_c0_g2	MKKA_DICDI^I	3.772758	0.000408	3.906981	7.2E-06
TRINITY_DN116292_c1_g1	P2C32_ORYSJ^	3.771877	5.08E-09	2.485644	0.000127
TRINITY_DN46578_c0_g3	#N/A	3.769574	4.59E-09	7.011568	2.46E-90
TRINITY_DN108624_c3_g1	HARB1_BOVIN	3.765517	7.38E-08	3.576761	5.47E-10
TRINITY_DN109315_c1_g1	C7091_ARATH	3.756877	5.64E-08	4.772703	4.7E-19
TRINITY_DN178657_c0_g1	#N/A	3.74683	1.23E-24	2.811079	1.09E-12
TRINITY_DN77713_c0_g3	#N/A	3.744043	4.86E-05	3.588995	1.38E-05
TRINITY_DN113735_c2_g1	#N/A	3.739583	2.71E-10	3.296559	4.6E-08
TRINITY_DN103280_c2_g2	#N/A	3.734158	6.85E-07	3.369998	8.61E-05
TRINITY_DN110450_c1_g1	P2C59_ORYSJ^	3.727466	0.000527	5.087936	2.01E-13
TRINITY_DN112930_c0_g6	#N/A	3.722742	2.27E-08	4.096749	2.03E-16
TRINITY_DN116767_c0_g1	BGH3B_BACO1	3.720218	1.49E-08	4.178638	1.01E-23

TRINITY_DN100615_c0_g3	ZAT10_ARATH	3.712883	0.000723	5.039256	2.31E-11
TRINITY_DN117561_c0_g1	NCED1_MAIZE	3.712805	3.35E-10	3.79098	1.64E-09
TRINITY_DN75919_c0_g2	#N/A	3.712772	2.68E-09	3.931454	1.64E-14
TRINITY_DN112270_c2_g4	#N/A	3.712385	2.62E-05	2.54796	0.00053
TRINITY_DN114890_c11_g11	RFS2_ARATH^I	3.710966	3.28E-19	4.300625	3.01E-68
TRINITY_DN118124_c0_g5	#N/A	3.710837	3.93E-06	4.021214	5.81E-14
TRINITY_DN114834_c4_g2	NAMB1_HORV	3.695913	0.000115	3.891142	1.59E-05
TRINITY_DN76472_c2_g1	#N/A	3.679412	5.23E-20	3.560597	6.69E-19
TRINITY_DN96674_c1_g3	PAO_MAIZE^P	3.679172	1.56E-25	2.519637	3.09E-18
TRINITY_DN199481_c0_g1	#N/A	3.666673	5.73E-13	3.65051	7.44E-15
TRINITY_DN198538_c0_g1	#N/A	3.665907	2.36E-09	3.577817	2.68E-11
TRINITY_DN19679_c0_g1	#N/A	3.66547	1.01E-19	1.681643	5.51E-05
TRINITY_DN113906_c0_g4	#N/A	3.653335	1.43E-07	3.008933	4.91E-09
TRINITY_DN140283_c1_g1	#N/A	3.648857	8.90E-21	3.102912	5.96E-18
TRINITY_DN78726_c0_g3	#N/A	3.637174	5.36E-07	3.296797	0.000173
TRINITY_DN71499_c0_g1	#N/A	3.633204	4.65E-08	4.174693	2.98E-13
TRINITY_DN119180_c2_g2	PAO_MAIZE^P	3.632282	1.59E-27	2.411509	1.41E-16
TRINITY_DN116683_c3_g6	R13L1_ARATH	3.623949	6.68E-15	2.107801	2.24E-06
TRINITY_DN82650_c1_g1	#N/A	3.622899	3.60E-09	3.599809	3.21E-07
TRINITY_DN100275_c1_g2	KCS11_ARATH	3.618289	4.01E-07	3.823455	1.99E-08
TRINITY_DN113838_c0_g1	7SB1_SOYBN^	3.616629	9.57E-06	5.113329	3.81E-21
TRINITY_DN1854_c0_g2	#N/A	3.588523	9.26E-10	3.244957	3.02E-06
TRINITY_DN95858_c0_g1	ALAXL_PYRHO	3.585743	1.18E-05	2.854151	0.000354
TRINITY_DN37180_c0_g4	#N/A	3.579214	3.59E-21	1.646247	0.000167
TRINITY_DN94429_c1_g1	#N/A	3.563069	0.000363	3.260181	0.000169
TRINITY_DN121157_c0_g1	#N/A	3.556217	2.09E-08	2.841398	0.000319
TRINITY_DN108864_c7_g9	ENDO4_ARATH	3.555697	0.000118	3.578213	0.000155
TRINITY_DN109353_c2_g3	P2C50_ORYSJ^	3.554095	1.37E-06	3.292783	1.68E-05
TRINITY_DN105964_c1_g1	NNJA1_ORYSJ^	3.544076	1.17E-05	3.960846	1.98E-08
TRINITY_DN120383_c6_g22	LOX1_ORYSJ^L	3.537267	2.16E-15	2.083713	2.15E-08
TRINITY_DN108658_c1_g1	P2C68_ORYSJ^	3.519054	0.000147	4.410047	2.22E-11
TRINITY_DN97082_c0_g1	FAD3C_RICCO^	3.508442	7.35E-05	3.876956	2.23E-06
TRINITY_DN87311_c0_g1	#N/A	3.504316	1.41E-17	2.468423	1.27E-12
TRINITY_DN89093_c0_g1	HSPR2_ARATH	3.503396	1.53E-08	2.423325	0.000176
TRINITY_DN92586_c0_g1	#N/A	3.495736	0.000103	3.312053	4.93E-06
TRINITY_DN28706_c0_g1	#N/A	3.494655	0.000544	4.063619	1.04E-05
TRINITY_DN92036_c8_g1	COMT1_MAIZE	3.479059	7.50E-31	2.984935	9.22E-50
TRINITY_DN86395_c1_g3	#N/A	3.473643	8.63E-17	2.603022	3.93E-09
TRINITY_DN105788_c1_g2	TI10C_ORYSI^T	3.46766	4.17E-09	3.559612	9.91E-12
TRINITY_DN112996_c0_g4	7SB1_SOYBN^	3.467409	8.74E-06	4.532902	8.54E-16
TRINITY_DN109571_c0_g2	#N/A	3.462081	1.08E-11	6.249859	1.68E-65
TRINITY_DN116698_c0_g1	DBAT_TAXCU^	3.456763	4.02E-05	2.941264	0.000287
TRINITY_DN105819_c0_g2	HSPR1_ARATH	3.45029	2.93E-13	2.544835	2.81E-06
TRINITY_DN59483_c0_g1	#N/A	3.435332	1.98E-26	2.534477	4.12E-15
TRINITY_DN117473_c1_g4	C94C1_ARATH	3.434332	6.32E-05	3.811081	4.37E-06
TRINITY_DN120711_c0_g1	#N/A	3.433907	3.40E-13	3.07073	1.75E-10
TRINITY_DN80885_c1_g1	#N/A	3.422466	8.00E-14	2.34404	1.16E-05

TRINITY_DN116388_c0_g1	COBL7_ARATH	3.421638	4.90E-06	2.640277	3.83E-07
TRINITY_DN108159_c1_g5	LOX1_HORVU^	3.410931	6.08E-12	2.887601	5.98E-08
TRINITY_DN120810_c1_g1	#N/A	3.395103	3.03E-21	1.673568	2.73E-07
TRINITY_DN207244_c0_g1	#N/A	3.393796	0.00098	3.578265	6.46E-05
TRINITY_DN117627_c1_g23	#N/A	3.39247	1.40E-05	2.9771	1.92E-05
TRINITY_DN221987_c0_g1	#N/A	3.39173	1.39E-14	3.157514	2.01E-13
TRINITY_DN180806_c0_g1	#N/A	3.390725	1.55E-11	3.124835	2.69E-10
TRINITY_DN101234_c0_g19	#N/A	3.39026	0.000105	3.715251	7.97E-07
TRINITY_DN112270_c2_g2	OGG1_ARATH^	3.372445	2.15E-05	3.373067	4.28E-08
TRINITY_DN119903_c0_g1	#N/A	3.363773	1.29E-15	1.773778	7.32E-05
TRINITY_DN141674_c0_g1	#N/A	3.362182	9.54E-08	2.589347	0.000202
TRINITY_DN109353_c2_g4	P2C50_ORYSJ^	3.356532	1.08E-08	2.308007	0.000261
TRINITY_DN107607_c0_g1	LOX1_HORVU^	3.356009	1.67E-11	2.845745	4.66E-07
TRINITY_DN103758_c0_g1	PLP2_ARATH^I	3.349021	3.91E-07	3.159313	7.41E-08
TRINITY_DN95133_c3_g2	COMT1_MAIZE	3.337614	3.28E-30	2.874316	1.45E-41
TRINITY_DN92692_c0_g2	#N/A	3.332696	3.62E-05	5.682127	2.12E-20
TRINITY_DN109843_c0_g6	NRX2_ORYSJ^I	3.325323	1.37E-10	4.392397	4.17E-24
TRINITY_DN108159_c1_g7	LOX1_HORVU^	3.313119	1.06E-06	2.880385	8E-07
TRINITY_DN113428_c0_g5	Y5707_ARATH^	3.299009	7.62E-07	3.441372	4.56E-08
TRINITY_DN91071_c0_g1	#N/A	3.296725	2.64E-06	3.596309	5.61E-12
TRINITY_DN72654_c0_g2	HIP26_ARATH^	3.289061	0.000668	3.863132	6.07E-06
TRINITY_DN111172_c1_g2	LEA14_GOSHI^	3.278234	2.76E-07	6.606709	1.25E-46
TRINITY_DN218288_c0_g1	#N/A	3.275433	1.67E-10	2.674829	1.53E-08
TRINITY_DN114890_c11_g10	RFS2_ARATH^I	3.263848	0.000266	4.25942	2.27E-12
TRINITY_DN102423_c1_g1	CRK10_ARATH	3.242117	4.77E-05	3.51861	3.95E-08
TRINITY_DN98809_c0_g1	KIC_ARATH^KI	3.241786	7.18E-07	1.532643	0.000584
TRINITY_DN109353_c1_g8	P2C50_ORYSJ^	3.215115	2.03E-08	2.58249	1.03E-07
TRINITY_DN106162_c0_g1	ANXD4_ARATH	3.194712	1.47E-08	2.542193	1.9E-06
TRINITY_DN84052_c0_g1	LEA14_GOSHI^	3.182292	1.81E-07	6.175317	3.14E-53
TRINITY_DN118526_c0_g2	GAE1_ARATH^	3.175899	6.96E-06	2.756019	4E-05
TRINITY_DN105373_c6_g5	ARG2_VIGRR^	3.169918	3.10E-06	4.183322	5.22E-16
TRINITY_DN37269_c0_g1	#N/A	3.168436	6.91E-09	3.046491	1.16E-06
TRINITY_DN117597_c0_g3	B3GTJ_ARATH^	3.165895	0.000508	3.395795	1.39E-05
TRINITY_DN114458_c6_g12	NAC2_ARATH^	3.164822	1.72E-12	3.583119	6.3E-23
TRINITY_DN116924_c1_g1	#N/A	3.150671	2.57E-14	2.226165	1.22E-07
TRINITY_DN115034_c2_g6	NAC48_ORYSJ^	3.132283	3.55E-11	3.423403	1.82E-15
TRINITY_DN119804_c2_g7	#N/A	3.130583	1.17E-05	3.315108	6.57E-15
TRINITY_DN116888_c0_g4	MSL4_ARATH^	3.128931	2.20E-07	4.740251	2.38E-32
TRINITY_DN111243_c0_g1	PTR18_ARATH	3.128414	3.65E-11	4.819477	2.92E-36
TRINITY_DN5036_c0_g1	#N/A	3.127186	7.94E-11	3.651587	4.45E-17
TRINITY_DN101625_c0_g1	PLP2_ARATH^I	3.100377	4.87E-07	3.177092	1.7E-11
TRINITY_DN140479_c0_g1	#N/A	3.098805	3.24E-19	2.170928	1.23E-10
TRINITY_DN108624_c2_g3	HARB1_BOVIN	3.093674	1.09E-06	3.053133	1.49E-11
TRINITY_DN20164_c0_g2	#N/A	3.092118	2.91E-08	6.500323	1.23E-69
TRINITY_DN70070_c0_g3	#N/A	3.091138	5.68E-08	3.04128	2.65E-07
TRINITY_DN160950_c2_g1	#N/A	3.08078	1.32E-21	2.260393	8.91E-13
TRINITY_DN105788_c3_g8	TI10C_ORYSJ^	3.079747	7.16E-06	3.10277	4.07E-05

TRINITY_DN114138_c0_g1	CLPB1_ARATH	3.073812	1.23E-05	3.705685	4.73E-13
TRINITY_DN217459_c0_g1	#N/A	3.073173	1.53E-18	2.53785	6.82E-15
TRINITY_DN104976_c3_g4	YCF23_PYRYE^	3.072995	2.29E-09	2.601724	3.09E-07
TRINITY_DN19613_c0_g1	#N/A	3.067775	7.75E-08	2.206561	0.000188
TRINITY_DN107906_c0_g1	P2C30_ORYSJ^	3.063759	8.56E-16	3.131559	7.91E-19
TRINITY_DN118674_c6_g1	AAP3_ARATH^	3.0604	1.07E-07	3.733643	1.44E-21
TRINITY_DN106643_c0_g1	E13B_WHEAT^	3.057304	9.18E-10	3.79151	4.04E-18
TRINITY_DN102501_c1_g6	ADF3_ORYSJ^A	3.041917	0.000115	4.352206	5.62E-16
TRINITY_DN118912_c3_g3	HARB1_HUMA	3.041901	4.70E-06	1.823511	0.000636
TRINITY_DN120383_c7_g9	LOX2_ORYSJ^L	3.040461	2.41E-11	1.839532	3.46E-06
TRINITY_DN113720_c0_g4	RLK1_ARATH^I	3.025269	0.000425	2.782199	9.59E-05
TRINITY_DN117424_c0_g2	TOLB_BDEBA^	3.016418	0.000243	4.092762	1.93E-12
TRINITY_DN105366_c1_g3	BURP3_ORYSJ	3.014198	1.16E-06	3.566295	8.75E-11
TRINITY_DN99620_c0_g1	BAM1_ARATH	3.009457	0.000313	5.583926	2E-28
TRINITY_DN117722_c2_g1	#N/A	3.005361	2.37E-09	6.43394	1.62E-48
TRINITY_DN114890_c11_g7	RFS2_ARATH^I	2.99253	2.33E-05	2.922499	9.69E-09
TRINITY_DN7376_c0_g1	#N/A	2.989523	2.90E-10	2.194292	0.000158
TRINITY_DN218554_c0_g1	#N/A	2.985856	2.49E-14	2.411598	2.83E-12
TRINITY_DN116665_c0_g4	BGH3B_BACO1	2.983787	7.57E-06	3.527539	5.29E-23
TRINITY_DN114637_c1_g4	ALF4_ARATH^A	2.979315	1.74E-17	2.912052	1.51E-25
TRINITY_DN22634_c0_g2	#N/A	2.975457	1.44E-05	2.398644	0.000469
TRINITY_DN106871_c0_g2	P2C30_ORYSJ^	2.968173	3.16E-11	2.944107	2.39E-13
TRINITY_DN111069_c1_g3	Y273_METTH^	2.965884	7.81E-09	4.315024	3.02E-30
TRINITY_DN106136_c1_g4	#N/A	2.964807	1.69E-06	2.215512	5.83E-05
TRINITY_DN19679_c1_g1	#N/A	2.960668	3.92E-11	2.82265	6.69E-10
TRINITY_DN113940_c1_g3	#N/A	2.957422	2.18E-08	3.557007	4.31E-15
TRINITY_DN101269_c2_g12	FD_ARATH^FD	2.942243	2.27E-05	3.364289	1.54E-05
TRINITY_DN93732_c1_g1	SL30A_ARATH	2.941951	2.39E-07	2.859755	8.3E-08
TRINITY_DN113960_c2_g2	ERF53_ARATH	2.94052	0.00066	3.672569	2.29E-07
TRINITY_DN83411_c3_g1	#N/A	2.937658	1.22E-15	2.564372	3.39E-14
TRINITY_DN70195_c0_g1	#N/A	2.936109	1.69E-07	2.948302	6.34E-11
TRINITY_DN110132_c0_g8	SPG20_BOVIN	2.925214	3.80E-05	2.979394	5.8E-08
TRINITY_DN92941_c1_g3	#N/A	2.924073	1.84E-07	3.271857	2.6E-15
TRINITY_DN25477_c0_g1	#N/A	2.922998	4.56E-11	2.995197	5.52E-14
TRINITY_DN72610_c0_g1	#N/A	2.921674	4.62E-12	3.19753	1.98E-15
TRINITY_DN97288_c2_g1	XIP1_WHEAT^	2.894378	6.45E-07	2.032784	0.000229
TRINITY_DN109013_c0_g1	AASS_ARATH^	2.892333	2.87E-14	3.071986	3.32E-17
TRINITY_DN104383_c8_g4	ERF80_ARATH	2.892218	4.05E-06	2.449776	6.95E-08
TRINITY_DN108864_c6_g7	ENDO2_ARATH	2.889491	5.45E-05	2.959042	2.8E-06
TRINITY_DN104863_c0_g6	GSTU1_ORYSI	2.887485	0.000434	3.584822	1.07E-08
TRINITY_DN105788_c3_g6	TI10C_ORYSJ^	2.884851	8.27E-11	3.248861	2.05E-09
TRINITY_DN114834_c5_g10	NAC48_ORYSJ	2.883484	4.41E-11	3.320481	4.06E-17
TRINITY_DN114458_c5_g2	NAC48_ORYSJ	2.880199	5.63E-06	3.107866	1.57E-09
TRINITY_DN106781_c1_g1	ENDO4_ARATH	2.878856	5.37E-05	2.900434	1.73E-06
TRINITY_DN36076_c0_g1	#N/A	2.871553	1.02E-07	3.007877	6.9E-07
TRINITY_DN115991_c0_g2	CLPB1_ORYSJ^	2.862123	0.000298	2.997093	8.59E-07
TRINITY_DN5036_c0_g2	#N/A	2.8581	4.13E-14	3.112372	7.47E-18

TRINITY_DN109946_c0_g5	MLO1_ARATH^	2.857234	0.000139	3.351997	2.06E-07
TRINITY_DN70395_c1_g1	#N/A	2.855643	9.21E-07	2.816405	1.78E-08
TRINITY_DN204193_c0_g1	#N/A	2.854024	4.46E-11	2.050774	6.68E-06
TRINITY_DN101997_c1_g2	DNAJ_CARHZ^	2.819159	7.48E-06	2.372273	0.000122
TRINITY_DN218131_c0_g1	#N/A	2.808807	4.06E-08	2.172162	1.55E-05
TRINITY_DN100283_c0_g4	BAM1_ARATH^	2.806947	0.000235	5.729316	6.05E-40
TRINITY_DN109946_c0_g1	MLO1_ARATH^	2.803212	1.42E-05	3.582026	5.71E-18
TRINITY_DN113998_c1_g2	TPP7_ORYSJ^T	2.799494	0.000265	2.846677	1.52E-05
TRINITY_DN118757_c0_g7	PTR53_ARATH	2.795099	1.03E-07	3.683374	2.64E-23
TRINITY_DN100140_c0_g3	BH035_ARATH	2.791815	0.000697	5.57605	1.12E-17
TRINITY_DN73803_c0_g1	#N/A	2.789789	5.92E-17	2.318574	3.29E-15
TRINITY_DN108658_c2_g2	P2C68_ORYSJ^	2.787685	1.90E-07	3.943829	1.74E-19
TRINITY_DN108658_c1_g2	P2C68_ORYSJ^	2.786391	0.000304	4.246398	3.53E-15
TRINITY_DN92976_c0_g6	P5CS_ORYSJ^P	2.779295	2.53E-05	4.208546	4.23E-19
TRINITY_DN103891_c0_g4	NRAM4_ORYS.	2.767797	3.28E-05	2.74669	1.7E-07
TRINITY_DN115525_c0_g5	CADH5_ORYSJ^	2.767737	2.33E-06	3.409278	2.56E-13
TRINITY_DN99277_c3_g1	#N/A	2.766866	7.10E-10	2.21021	6.83E-09
TRINITY_DN112876_c0_g2	AB1F_ARATH^	2.757671	6.31E-07	4.108656	1.9E-20
TRINITY_DN109215_c0_g7	WRK40_ARATH	2.752236	1.75E-09	1.823417	0.000726
TRINITY_DN116800_c1_g2	#N/A	2.750661	1.06E-06	3.270299	1.1E-17
TRINITY_DN118519_c0_g1	CTR1_ARATH^	2.747491	0.000103	3.700728	1.88E-12
TRINITY_DN115525_c0_g8	CADH5_ORYSJ^	2.736716	5.62E-08	3.274717	1.24E-18
TRINITY_DN105792_c3_g4	Y1725_ARATH^	2.73429	1.66E-05	2.54157	1.23E-05
TRINITY_DN178641_c0_g1	#N/A	2.729887	1.44E-18	1.828992	7.15E-10
TRINITY_DN109691_c1_g1	AASS_ARATH^	2.727032	4.44E-08	2.779334	2.97E-10
TRINITY_DN113417_c2_g3	#N/A	2.721171	9.67E-06	6.091013	1.24E-48
TRINITY_DN104651_c1_g4	DHAR2_ARATH	2.710656	1.71E-10	2.303033	1.35E-08
TRINITY_DN112159_c2_g2	SGR_ORYSJ^SG	2.709265	8.17E-08	3.114776	4.09E-15
TRINITY_DN119270_c1_g2	EGY3_ORYSJ^E	2.690035	3.22E-06	1.859268	0.000517
TRINITY_DN33225_c0_g1	#N/A	2.690033	2.60E-12	2.709028	3.06E-16
TRINITY_DN113409_c0_g1	#N/A	2.689329	6.13E-05	2.999068	3.09E-09
TRINITY_DN117986_c0_g1	CTR1_ARATH^	2.68402	0.000745	3.772907	2.45E-14
TRINITY_DN116784_c2_g2	ZFP1_WHEAT^	2.679133	1.27E-05	1.804087	8.63E-05
TRINITY_DN105192_c1_g2	LPP3_ARATH^I	2.677658	3.01E-12	2.069492	4.9E-05
TRINITY_DN109328_c0_g3	LRK41_ARATH^	2.66271	0.000442	2.391351	0.000166
TRINITY_DN72610_c0_g2	#N/A	2.662436	2.57E-05	2.954744	2.73E-07
TRINITY_DN124087_c0_g1	#N/A	2.658685	3.84E-09	1.790309	1.37E-05
TRINITY_DN109433_c5_g2	E13B_WHEAT^	2.648905	6.42E-09	3.197981	5.92E-13
TRINITY_DN106873_c0_g1	PTR53_ARATH	2.640954	4.18E-07	3.529652	3.27E-21
TRINITY_DN37526_c0_g1	CSPL7_ORYSJ^	2.63182	0.000197	2.761044	2.51E-08
TRINITY_DN65040_c0_g2	#N/A	2.627393	1.03E-06	3.079172	5.28E-10
TRINITY_DN109511_c1_g1	PTR18_ARATH	2.625868	1.17E-07	4.382183	1.47E-26
TRINITY_DN7389_c0_g1	#N/A	2.62336	7.28E-09	2.048595	1.71E-06
TRINITY_DN117597_c1_g1	B3GTH_ARATH	2.613395	0.000731	2.383216	0.000252
TRINITY_DN114458_c6_g35	NAMB2_TRITD	2.613269	6.81E-05	2.880635	3.25E-07
TRINITY_DN178757_c0_g1	#N/A	2.606382	6.47E-09	1.773522	3.21E-05
TRINITY_DN117630_c2_g3	PAO2_ARATH^	2.600487	2.03E-08	4.178341	1.4E-24

TRINITY_DN86816_c0_g1	TI11D_ORYSI^	2.58091	2.06E-06	3.173054	1.33E-08
TRINITY_DN111786_c0_g6	KCS11_ARATH	2.56911	7.47E-05	3.130383	3.29E-10
TRINITY_DN120607_c2_g1	#N/A	2.558793	7.44E-21	2.199475	1.03E-17
TRINITY_DN121062_c0_g1	#N/A	2.552535	8.29E-05	2.746064	1.23E-06
TRINITY_DN106194_c0_g1	AVT1_YEAST^A	2.550884	0.000384	5.514045	3.69E-38
TRINITY_DN104776_c0_g1	SDT1_YEAST^S	2.548152	1.42E-05	2.232755	5.02E-05
TRINITY_DN103663_c0_g1	NNJA1_ORYSJ^	2.544839	1.44E-06	2.530876	2.78E-07
TRINITY_DN107169_c0_g1	ANXD4_ARATH	2.544056	1.30E-05	2.050954	6.54E-05
TRINITY_DN113630_c2_g1	SUS4_ORYSJ^S	2.540806	0.000605	4.430511	4.63E-26
TRINITY_DN108487_c0_g2	AVT1_YEAST^A	2.534388	0.000693	5.051249	4.43E-38
TRINITY_DN115326_c0_g1	GPDH3_ORYSJ	2.526106	0.000529	3.321062	2.72E-08
TRINITY_DN221120_c0_g1	#N/A	2.520996	1.41E-06	2.376777	1.13E-05
TRINITY_DN118650_c0_g1	HEN1_ARATH^	2.520795	0.000504	3.994126	1.31E-16
TRINITY_DN98997_c0_g1	E13F_HORVU^	2.518428	0.000529	2.296233	0.000452
TRINITY_DN159556_c1_g1	#N/A	2.51842	5.00E-15	2.571013	4.15E-25
TRINITY_DN112159_c1_g3	SGR_ORYSJ^SG	2.514008	1.13E-07	2.804738	1.46E-12
TRINITY_DN83411_c2_g1	#N/A	2.513297	2.88E-06	2.667582	5.38E-07
TRINITY_DN112614_c2_g4	DRL45_ARATH	2.508525	4.44E-08	2.113009	1.01E-06
TRINITY_DN110344_c0_g3	CLPB1_ORYSJ^	2.506214	0.000335	5.768612	1.84E-57
TRINITY_DN109226_c1_g1	NAC56_ARATH	2.50181	0.000401	2.15103	0.000285
TRINITY_DN118123_c0_g2	MSL6_ARATH^	2.495279	1.17E-06	4.059167	4.87E-24
TRINITY_DN99277_c2_g1	#N/A	2.494456	1.87E-16	2.133107	5.54E-10
TRINITY_DN98967_c3_g2	#N/A	2.492416	3.78E-09	2.833312	1.22E-13
TRINITY_DN97261_c2_g2	#N/A	2.489497	7.95E-07	2.31691	1.55E-06
TRINITY_DN112683_c1_g3	SSL10_ARATH^	2.489281	1.57E-08	1.772144	5.06E-06
TRINITY_DN116784_c1_g1	ZFP1_WHEAT^	2.483203	3.87E-08	1.835753	3.04E-07
TRINITY_DN64973_c0_g2	#N/A	2.476807	3.01E-05	2.46503	9.57E-06
TRINITY_DN76472_c4_g1	#N/A	2.47594	4.95E-13	2.103787	2.68E-10
TRINITY_DN120046_c2_g3	SCRL2_ORYSJ^	2.475069	0.000158	2.141112	0.000984
TRINITY_DN109873_c1_g1	AAP3_ARATH^	2.468283	3.40E-06	2.706103	2.12E-09
TRINITY_DN105233_c0_g2	#N/A	2.460163	1.16E-07	1.803862	0.000641
TRINITY_DN96274_c1_g4	#N/A	2.444133	6.50E-06	2.260574	0.00014
TRINITY_DN115029_c0_g1	GAE1_ARATH^	2.442773	0.000317	2.43717	4.08E-07
TRINITY_DN112708_c1_g3	GPDH3_ORYSJ	2.407503	0.000868	3.502207	1.67E-10
TRINITY_DN217460_c0_g1	#N/A	2.404618	9.02E-06	2.068598	3.13E-05
TRINITY_DN91530_c0_g2	CML15_ORYSJ	2.39698	0.000198	1.844799	0.000231
TRINITY_DN100278_c0_g1	C3H41_ORYSJ^	2.389582	0.000631	2.516705	7.94E-05
TRINITY_DN102786_c0_g1	PPA17_ARATH	2.384824	1.12E-06	1.864921	2.1E-06
TRINITY_DN108097_c0_g9	YCF23_PYRYE^	2.384563	3.23E-06	2.003627	2.85E-05
TRINITY_DN101269_c2_g5	FD_ARATH^FD	2.383073	0.00017	2.798503	1.82E-06
TRINITY_DN114423_c1_g1	E13B_WHEAT^	2.379673	7.71E-06	3.358815	3.39E-15
TRINITY_DN92844_c2_g1	#N/A	2.355858	1.93E-06	4.346091	2.67E-64
TRINITY_DN73120_c0_g2	AGT23_ARATH	2.349604	5.85E-06	4.286045	4.16E-32
TRINITY_DN110136_c0_g3	#N/A	2.34934	2.36E-09	2.188929	2.63E-10
TRINITY_DN140264_c0_g1	#N/A	2.349078	3.61E-13	2.20069	1.83E-17
TRINITY_DN115847_c0_g3	DBNBT_TAXCA	2.345456	0.000942	2.134599	0.000719
TRINITY_DN118863_c0_g1	#N/A	2.335193	0.000168	2.842915	1.1E-06

TRINITY_DN101635_c1_g1	PPA17_ARATH	2.33487	8.44E-11	1.611282	1.68E-06
TRINITY_DN110963_c0_g1	TCPE2_AVESA^	2.325759	0.000212	1.999854	0.000184
TRINITY_DN113408_c1_g1	SUS4_ORYSJ^S	2.320098	0.000814	4.144319	6.58E-39
TRINITY_DN86957_c0_g1	TI11D_ORYSI^T	2.316537	0.000129	2.973402	2.14E-07
TRINITY_DN141730_c0_g1	#N/A	2.310926	3.11E-10	2.094194	7.49E-07
TRINITY_DN114921_c0_g21	P5CS_ORYSJ^P	2.310184	9.23E-06	3.716951	8.66E-25
TRINITY_DN91530_c0_g4	CML15_ORYSJ^	2.310149	0.000827	2.036109	8.2E-06
TRINITY_DN109368_c2_g1	ATL31_ARATH^	2.291061	0.000418	2.702461	1.42E-09
TRINITY_DN112565_c0_g1	AB1F_ARATH^.	2.280152	0.000212	3.500357	3.96E-15
TRINITY_DN100762_c1_g2	#N/A	2.277553	2.78E-05	3.852729	2.09E-25
TRINITY_DN113998_c1_g3	TPP6_ORYSJ^T	2.276992	0.000486	3.306161	2.04E-11
TRINITY_DN101909_c1_g13	ERF80_ARATH^	2.264712	0.000109	2.170473	6.22E-05
TRINITY_DN39832_c0_g1	#N/A	2.242304	0.000804	2.505653	4.74E-05
TRINITY_DN110410_c3_g1	VSR1_ARATH^V	2.226332	1.19E-06	2.296347	5.76E-09
TRINITY_DN115103_c0_g1	#N/A	2.223401	4.95E-08	2.249654	1.11E-10
TRINITY_DN116324_c3_g2	PAO2_ARATH^	2.208736	1.40E-10	3.611669	1.76E-21
TRINITY_DN141622_c0_g1	#N/A	2.20169	0.000262	2.460879	8.62E-06
TRINITY_DN140459_c0_g1	#N/A	2.197952	9.76E-06	1.966595	7.8E-06
TRINITY_DN109643_c6_g2	BH035_ARATH	2.188789	2.64E-05	4.640258	3.49E-18
TRINITY_DN62482_c1_g2	NLTP_VIGUN^I	2.185336	9.89E-07	1.506888	0.00047
TRINITY_DN107820_c0_g6	P2C50_ORYSJ^	2.183287	4.95E-06	1.973281	4.64E-05
TRINITY_DN113953_c0_g5	C3H2_ORYSJ^C	2.173574	0.000742	3.312855	3.6E-11
TRINITY_DN117175_c0_g2	POLX_TOBAC^	2.171956	2.56E-05	4.340027	4.46E-26
TRINITY_DN97587_c2_g4	#N/A	2.168818	1.21E-13	1.582159	3.41E-10
TRINITY_DN82146_c1_g1	#N/A	2.165139	0.000127	2.090763	2.89E-05
TRINITY_DN115423_c3_g5	P5CS_ORYSJ^P	2.164298	0.000196	2.9493	6.07E-17
TRINITY_DN107983_c1_g1	ENL1_ARATH^	2.163863	0.000155	4.854737	3.05E-35
TRINITY_DN141402_c0_g1	#N/A	2.160914	2.26E-12	2.050677	2.89E-13
TRINITY_DN97034_c1_g3	#N/A	2.157792	1.22E-11	1.548155	4.61E-09
TRINITY_DN36607_c0_g2	C3H41_ORYSJ^	2.154282	3.42E-06	2.028032	4.13E-06
TRINITY_DN141588_c0_g1	#N/A	2.144283	2.70E-11	1.980154	6.19E-13
TRINITY_DN105401_c1_g3	HOX22_ORYSJ^	2.143756	9.06E-05	2.980378	2.03E-17
TRINITY_DN113061_c0_g4	YB95_ARATH^Y	2.142511	0.000403	2.215381	3.8E-07
TRINITY_DN112721_c0_g4	LOX3_ARATH^	2.135458	3.51E-05	1.60825	0.00062
TRINITY_DN109484_c0_g1	SL30A_ARATH^	2.127221	1.64E-05	2.120739	1.28E-05
TRINITY_DN106136_c1_g11	#N/A	2.119453	3.28E-07	2.248726	3.95E-08
TRINITY_DN110517_c1_g2	E134_ARATH^I	2.115512	0.000889	3.673706	5.84E-21
TRINITY_DN109821_c5_g1	#N/A	2.10753	3.05E-12	2.014158	1.43E-14
TRINITY_DN35178_c1_g1	#N/A	2.092702	5.65E-14	1.608637	2.5E-11
TRINITY_DN109845_c1_g4	SPG20_BOVIN^	2.090498	0.000667	2.26859	5.29E-05
TRINITY_DN106577_c0_g2	MCU2_ARATH^	2.088432	0.000218	2.361341	1.88E-08
TRINITY_DN99215_c0_g2	RBM42_XENTR	2.088156	0.000319	2.042953	5.03E-05
TRINITY_DN105234_c1_g20	RNLE_SOLLC^R	2.086596	1.25E-06	2.243168	1.01E-13
TRINITY_DN99647_c0_g3	UVB31_ARATH	2.086025	2.86E-06	3.877865	1.11E-42
TRINITY_DN105233_c4_g6	RNS1_ARATH^	2.082227	0.000178	1.989061	0.000145
TRINITY_DN112721_c0_g3	LOX5_ORYSJ^L	2.08023	0.000294	1.579634	0.000379
TRINITY_DN104116_c0_g1	WRK26_ARATH	2.078641	7.93E-07	1.759705	4.41E-09

TRINITY_DN118259_c1_g3	POLX_TOBAC^	2.077576	8.17E-05	1.999855	3.7E-08
TRINITY_DN44389_c0_g1	C3H41_ORYSJ^	2.074651	7.15E-06	1.940946	1.01E-05
TRINITY_DN95051_c0_g1	AOC1_ARATH^	2.073241	0.000561	2.170333	1.16E-06
TRINITY_DN103766_c1_g1	GSTU1_ORYSI^	2.067839	6.05E-06	2.824175	5.64E-21
TRINITY_DN104743_c0_g3	TET8_ARATH^T	2.060854	9.45E-07	1.987865	5.99E-09
TRINITY_DN98967_c4_g1	#N/A	2.060844	0.000159	2.486367	1.46E-05
TRINITY_DN105601_c1_g1	SDT1_YEAST^S	2.054337	0.000142	1.879226	4.84E-05
TRINITY_DN106330_c1_g2	#N/A	2.040894	1.78E-08	1.46514	1.07E-05
TRINITY_DN94631_c1_g2	P5CS_ORYSJ^P	2.040201	0.000325	2.96361	5.85E-12
TRINITY_DN198248_c0_g1	#N/A	2.038651	4.44E-08	1.579714	5.65E-05
TRINITY_DN104370_c0_g2	IAAT_MAIZE^I/	2.031643	0.000987	2.54835	5.74E-06
TRINITY_DN108565_c2_g1	UVB31_ARATH	2.025571	3.18E-05	3.817753	9.81E-40
TRINITY_DN221490_c0_g1	#N/A	2.023386	2.85E-05	1.497122	3.97E-05
TRINITY_DN23245_c0_g1	#N/A	2.007029	0.000167	1.882858	0.000212
TRINITY_DN128678_c0_g1	#N/A	2.00504	6.08E-05	2.441922	3.56E-09
TRINITY_DN102877_c1_g1	#N/A	1.988198	0.000301	1.992624	0.000216
TRINITY_DN110766_c0_g1	#N/A	1.974445	0.000231	2.405305	6.48E-07
TRINITY_DN97510_c3_g2	#N/A	1.972442	4.37E-06	2.070863	3.79E-09
TRINITY_DN106800_c2_g3	PTR18_ARATH	1.958314	1.08E-05	2.606688	9.64E-13
TRINITY_DN102167_c0_g1	SCP18_ARATH-	1.948121	0.000911	2.502707	4.41E-07
TRINITY_DN108450_c1_g10	NFYA1_ARATH	1.947713	3.85E-06	1.771332	6.14E-07
TRINITY_DN67141_c0_g1	#N/A	1.947656	9.94E-07	1.880858	1.01E-08
TRINITY_DN108864_c7_g1	ENDO2_ARATH	1.945893	5.24E-05	1.866222	5.81E-05
TRINITY_DN109790_c1_g1	CIGR1_ORYSJ^	1.944867	0.000246	2.293811	2.05E-07
TRINITY_DN105234_c1_g25	RNS1_ARATH^	1.940788	7.80E-05	2.313099	7.78E-11
TRINITY_DN27742_c0_g1	#N/A	1.938774	3.10E-06	2.562827	3.96E-13
TRINITY_DN109312_c1_g1	PPO_MALDO^	1.934909	8.91E-05	1.939257	0.000211
TRINITY_DN109657_c1_g2	#N/A	1.934752	0.000988	6.057393	1.4E-46
TRINITY_DN92761_c1_g1	#N/A	1.926544	7.33E-07	3.943101	8.48E-47
TRINITY_DN78097_c0_g1	LT02_HORVU^	1.918442	1.11E-05	2.335952	2.31E-14
TRINITY_DN98035_c1_g1	#N/A	1.918087	0.000783	2.129155	5.02E-05
TRINITY_DN116556_c4_g10	LOX23_HORVU	1.916708	0.000116	2.003857	1.12E-06
TRINITY_DN117754_c2_g2	ANXD1_ARATH	1.907781	0.000211	1.656719	0.000179
TRINITY_DN117625_c0_g1	POLX_TOBAC^	1.900961	0.000259	1.870117	7.1E-05
TRINITY_DN107908_c0_g1	EDR2_ARATH^	1.896694	0.000992	2.337734	4.3E-09
TRINITY_DN114494_c0_g5	#N/A	1.891177	8.26E-06	1.973791	2.81E-07
TRINITY_DN105233_c4_g1	RNS1_ARATH^	1.876142	1.29E-05	2.394531	7.1E-10
TRINITY_DN109558_c5_g2	AL7A1_MALDC	1.874482	8.89E-06	2.010534	1.04E-08
TRINITY_DN105234_c1_g33	RNLE_SOLLC^F	1.863481	0.000295	2.749094	1.07E-13
TRINITY_DN101795_c0_g9	DI191_ORYSJ^	1.851602	0.000171	1.906371	0.000314
TRINITY_DN47245_c0_g1	#N/A	1.841215	3.29E-09	1.431366	7.16E-08
TRINITY_DN104775_c2_g1	PER3_ARATH^	1.831574	0.000451	2.077894	1.26E-05
TRINITY_DN113257_c0_g1	CIPKT_ORYSJ^(	1.827784	0.000442	2.053534	7.68E-06
TRINITY_DN120037_c4_g2	#N/A	1.824166	0.000288	2.873611	3.62E-13
TRINITY_DN106699_c0_g1	NAC67_ORYSJ^	1.823977	0.000373	3.513173	1.55E-28
TRINITY_DN117634_c3_g1	DMR6_ARATH-	1.822011	2.13E-05	2.869888	8.55E-12
TRINITY_DN109364_c0_g1	SPG20_HUMAI	1.818162	4.95E-07	2.955933	4.4E-17

TRINITY_DN114998_c2_g16	OCT7_ARATH^	1.814708	7.63E-07	3.008552	3.39E-19
TRINITY_DN109233_c1_g6	DMR6_ARATH	1.810869	0.000834	2.500816	1.28E-12
TRINITY_DN113763_c0_g2	GUX1_ARATH^	1.810805	0.000243	2.414325	1.82E-11
TRINITY_DN104651_c1_g1	DHAR2_ARATH	1.805084	0.000242	1.685931	0.000169
TRINITY_DN117754_c2_g3	ANXD1_ARATH	1.800421	0.000525	1.893177	9.39E-07
TRINITY_DN117330_c1_g1	ROC5_ORYSJ^I	1.800071	0.000157	1.465449	0.000825
TRINITY_DN104617_c3_g2	#N/A	1.794662	2.64E-07	2.575914	1.12E-15
TRINITY_DN76023_c0_g2	#N/A	1.793496	5.30E-06	2.038329	1.24E-11
TRINITY_DN108689_c3_g5	AL7A1_MALDC	1.790673	0.000125	1.872754	1.5E-05
TRINITY_DN105401_c1_g2	HOX22_ORYSJ	1.774873	0.000167	2.520018	6.95E-13
TRINITY_DN108689_c3_g6	AL7B4_ARATH	1.773977	2.73E-05	2.228079	3.95E-13
TRINITY_DN119409_c0_g1	NPC1_HUMAN	1.773185	0.000989	2.338005	3.45E-11
TRINITY_DN106180_c0_g1	NNJA2_WHEAT	1.768812	5.81E-05	1.874209	1.31E-09
TRINITY_DN114921_c0_g11	P5CS_ORYSJ^P	1.768282	1.11E-05	1.93901	6.4E-10
TRINITY_DN103497_c0_g1	UREF_ORYSJ^U	1.765858	0.000116	2.996018	6.69E-19
TRINITY_DN112043_c0_g1	CIPKT_ORYSJ^	1.761946	0.000908	2.048934	1.84E-06
TRINITY_DN105373_c6_g6	LEA5_CITSI^LE	1.757051	0.000238	1.983478	1.23E-07
TRINITY_DN200046_c0_g1	#N/A	1.755371	0.000169	1.950743	1.39E-06
TRINITY_DN110664_c2_g3	PPCK1_ARATH	1.74411	3.77E-05	1.434006	2.73E-05
TRINITY_DN103874_c1_g1	TET8_ARATH^T	1.744028	5.67E-06	1.502465	1.91E-06
TRINITY_DN179810_c0_g1	#N/A	1.732128	7.93E-05	1.588652	0.000244
TRINITY_DN111449_c1_g4	ODPA2_ORYSJ	1.7314	0.000914	3.021745	3.64E-15
TRINITY_DN106393_c1_g5	SPSY_ARATH^S	1.731364	0.00025	1.86669	7.67E-08
TRINITY_DN92488_c0_g1	#N/A	1.728301	0.000206	1.4515	3.32E-05
TRINITY_DN104648_c0_g1	WRK26_ARATH	1.717471	9.00E-05	1.722707	6.82E-07
TRINITY_DN105401_c1_g4	HOX6_ORYSJ^I	1.71434	0.000675	1.912687	4.87E-07
TRINITY_DN109643_c5_g4	#N/A	1.707838	3.76E-05	1.868882	4.62E-09
TRINITY_DN36607_c0_g3	C3H41_ORYSJ	1.706742	2.93E-05	1.828294	1.55E-07
TRINITY_DN114447_c0_g1	SCP18_ARATH	1.706193	0.000272	2.224374	1.34E-08
TRINITY_DN114834_c5_g1	NAC48_ORYSJ	1.700977	2.09E-08	2.468837	2.09E-20
TRINITY_DN108106_c1_g1	TMN11_ARATH	1.691271	0.00043	1.467525	0.000726
TRINITY_DN117754_c1_g1	ANXD1_ARATH	1.688886	0.000152	1.792477	1.79E-08
TRINITY_DN114172_c0_g1	CSCLD_ARATH	1.679404	5.60E-05	2.058429	1.37E-09
TRINITY_DN111846_c0_g2	AL121_ARATH	1.678581	2.04E-05	1.621793	3.37E-07
TRINITY_DN119470_c2_g7	NOG1_ARATH	1.671031	0.000107	1.51707	5.88E-05
TRINITY_DN112611_c1_g4	TCMO_CATRO	1.667432	0.000803	3.115329	1.06E-17
TRINITY_DN99970_c0_g1	HPPD_HORVU	1.655279	0.000188	2.853533	7.89E-21
TRINITY_DN116470_c2_g1	SBT14_ARATH	1.653079	0.000164	2.15171	1.96E-07
TRINITY_DN106250_c2_g1	BADH_HORVU	1.649628	9.69E-05	2.561109	6.97E-18
TRINITY_DN104535_c4_g3	PPCK1_ARATH	1.638696	0.00015	1.560754	2.87E-07
TRINITY_DN111033_c1_g38	RA213_ARATH	1.618975	0.000631	1.836156	2.14E-05
TRINITY_DN72852_c0_g1	LT02_HORVU^	1.615745	0.000716	2.142435	5.89E-12
TRINITY_DN88492_c1_g1	#N/A	1.607171	8.96E-06	1.357063	0.000343
TRINITY_DN116194_c2_g1	PLST3_ARATH	1.571425	0.000107	2.363955	3.09E-12
TRINITY_DN105940_c1_g2	CO410_WHEAT	1.569794	0.000145	3.253596	2.44E-24
TRINITY_DN110327_c1_g1	NFYA1_ARATH	1.564803	0.000147	1.577656	2.72E-06
TRINITY_DN44389_c0_g2	C3H41_ORYSJ	1.561921	0.000246	1.632222	1.02E-05

TRINITY_DN106894_c1_g2	SPSY_ARATH^S	1.561017	9.80E-05	1.389911	1.03E-05
TRINITY_DN112556_c0_g1	PTR33_ARATH	1.528172	4.04E-05	3.543542	1.11E-29
TRINITY_DN105454_c0_g11	DHAR2_ARATH	1.510479	2.42E-05	1.423315	1.48E-06
TRINITY_DN93912_c1_g3	UGT2_GARJA^	1.482485	0.00083	2.288232	6.42E-14
TRINITY_DN108747_c1_g1	CIPKV_ORYSJ^	1.467829	0.000197	2.459141	5.26E-17
TRINITY_DN106180_c1_g1	NNJA2_WHEAT	1.434317	0.000671	1.776823	8.85E-09
TRINITY_DN217901_c0_g1	#N/A	1.424911	0.000336	1.253743	0.000506
TRINITY_DN103702_c1_g1	PER1_SORBI^P	1.398619	0.000617	2.007745	5.02E-10
TRINITY_DN117939_c4_g6	LOX22_HORVU	1.387475	0.000395	1.120867	0.000942
TRINITY_DN108197_c1_g1	PPH_ARATH^P	1.377213	0.000527	1.350273	0.000134
TRINITY_DN111389_c0_g2	HFC1B_ORYSJ^	1.357372	0.000109	1.994539	1.02E-14
TRINITY_DN106054_c2_g9	CO410_WHEAT	1.342484	0.00012	2.894785	2.77E-24
TRINITY_DN105806_c2_g3	#N/A	1.311007	8.76E-05	1.511932	2.34E-07
TRINITY_DN96795_c0_g1	H1_SOLPN^H1	1.264709	0.000257	1.507774	1.84E-06
TRINITY_DN90207_c0_g1	CYSP1_MAIZE^	1.25404	0.00062	1.164376	9.32E-05
TRINITY_DN115182_c1_g2	YTHD1_MOUS	1.221951	6.67E-05	1.310981	1.36E-07
TRINITY_DN91743_c1_g3	SODC2_ORYSJ^	1.221469	0.000346	1.358469	1.91E-07
TRINITY_DN114870_c0_g1	YTHD1_MOUS	1.178361	9.40E-05	1.423761	1.13E-06
TRINITY_DN95767_c2_g1	PSAE_HORVU^	-1.04761	0.000543	-1.8503	1.51E-11
TRINITY_DN111713_c5_g12	HOX16_ORYSJ^	-1.13024	0.000413	-1.33479	1.9E-08
TRINITY_DN96282_c3_g3	PSAE_HORVU^	-1.15725	0.000153	-1.90142	5.88E-13
TRINITY_DN103454_c2_g6	DSP4_ARATH^	-1.16097	0.00043	-1.79274	1.4E-10
TRINITY_DN99966_c0_g2	PSAD_HORVU^	-1.16206	0.000517	-1.17695	1.73E-05
TRINITY_DN96311_c0_g1	#N/A	-1.20233	0.000429	-1.62885	1.3E-09
TRINITY_DN110981_c0_g1	EFGC_ARATH^	-1.21113	9.69E-05	-0.94515	9.25E-05
TRINITY_DN100905_c1_g1	ZRP4_MAIZE^Z	-1.24211	0.000711	-2.3459	6.65E-18
TRINITY_DN120021_c4_g4	CCS1_ORYSJ^C	-1.24444	0.000837	-1.61033	7.86E-07
TRINITY_DN104504_c0_g1	HEM11_HORV	-1.26364	0.000684	-1.22272	0.000252
TRINITY_DN86676_c0_g1	FQR1_ARATH^	-1.2747	0.000576	-1.83619	1.04E-07
TRINITY_DN103391_c1_g5	DSP4_CASSA^I	-1.28174	0.000363	-1.6254	9.55E-09
TRINITY_DN106714_c7_g12	GRP_HORVU^	-1.28707	0.000854	-0.97562	1.15E-07
TRINITY_DN101527_c7_g6	GLO5_ORYSJ^	-1.29359	0.000493	-2.01651	3.6E-16
TRINITY_DN104392_c0_g1	#N/A	-1.30938	0.000713	-1.06487	0.000433
TRINITY_DN64898_c0_g1	RR16_AGRST^I	-1.31519	5.59E-05	-1.74839	5.74E-09
TRINITY_DN98954_c4_g4	PSBP_WHEAT^	-1.32833	0.000437	-2.19086	7.51E-12
TRINITY_DN100880_c1_g1	COL9_ARATH^	-1.32863	0.000207	-1.1719	1.14E-05
TRINITY_DN95522_c7_g3	Y1426_METJA^	-1.33126	0.000354	-1.47644	8.65E-07
TRINITY_DN105798_c0_g1	CHLI_ORYSJ^C	-1.33284	0.000687	-1.06364	0.000204
TRINITY_DN108226_c1_g1	#N/A	-1.33726	0.000155	-0.88545	0.000763
TRINITY_DN89584_c0_g1	HAP28_RAT^H	-1.33769	0.000633	-1.48047	1.13E-06
TRINITY_DN98622_c3_g1	UEV1C_ARATH	-1.33789	0.000666	-1.3813	5.14E-06
TRINITY_DN119193_c1_g2	ALGC_PSESM^	-1.33796	0.000294	-1.23409	4.08E-05
TRINITY_DN116491_c1_g1	IF2C_ARATH^I	-1.34006	2.38E-05	-1.06262	2.7E-05
TRINITY_DN115469_c6_g2	FENR1_ORYSJ^	-1.3408	5.63E-05	-1.16171	1.29E-05
TRINITY_DN94745_c3_g1	ATPX_SPIOL^A	-1.34331	4.90E-06	-1.19291	3.23E-08
TRINITY_DN85148_c3_g1	PSAK_HORVU^	-1.34555	1.23E-05	-2.29027	3.8E-16
TRINITY_DN104536_c0_g1	FAD6C_BRANA	-1.347	4.82E-05	-1.42252	2.58E-06

TRINITY_DN87826_c5_g1	PSAK_HORVU^	-1.34705	0.000371	-2.57589	1.05E-15
TRINITY_DN99693_c0_g1	EBFC2_ARATH	-1.3492	0.000393	-1.14598	0.000664
TRINITY_DN104668_c0_g1	FAD6C_BRANA	-1.35439	0.00018	-1.24473	4.27E-06
TRINITY_DN108769_c2_g2	PNSB1_ARATH	-1.35588	0.000636	-2.0114	1.96E-08
TRINITY_DN111995_c9_g3	GLYM1_ARATH	-1.35749	1.58E-05	-1.5303	2.35E-16
TRINITY_DN117458_c1_g1	SUT33_ARATH	-1.36413	0.00025	-1.56856	7.68E-07
TRINITY_DN105798_c1_g1	CHLI_ORYSJ^CI	-1.38443	6.47E-05	-0.97972	0.000487
TRINITY_DN117305_c5_g24	TKTC_MAIZE^T	-1.38774	7.41E-05	-1.45333	1.42E-06
TRINITY_DN113271_c1_g5	PSAD_HORVU^	-1.38995	0.000188	-1.45843	1.16E-07
TRINITY_DN111585_c10_g95	THI42_SORBI^	-1.39081	0.000451	-1.16126	0.000406
TRINITY_DN110247_c1_g4	PNSB1_ARATH	-1.40065	0.000251	-2.40416	2.06E-11
TRINITY_DN97276_c5_g1	Y1426_METJA^	-1.42219	0.000222	-1.54685	1.13E-07
TRINITY_DN113823_c0_g1	ZRP4_MAIZE^Z	-1.4222	6.32E-05	-2.29425	9.64E-17
TRINITY_DN109321_c7_g3	ATPX_SPIOL^A	-1.42308	0.000689	-1.01202	0.000432
TRINITY_DN100252_c1_g3	THF1_ORYSJ^T	-1.43073	0.00023	-1.39187	7.13E-05
TRINITY_DN99529_c2_g1	IPYR_ORYSJ^IP	-1.45203	9.57E-06	-2.82452	4E-21
TRINITY_DN115469_c6_g71	FENR1_ORYSJ^	-1.46998	0.000126	-1.58895	3.08E-06
TRINITY_DN96983_c0_g2	PNSL3_ARATH	-1.47478	0.000346	-3.03489	4.22E-13
TRINITY_DN87706_c3_g1	PSAN_HORVU^	-1.47585	0.000347	-2.73771	1.14E-13
TRINITY_DN97323_c0_g1	CYT1_ORYSJ^C	-1.47756	4.43E-05	-1.08118	1.47E-05
TRINITY_DN108357_c0_g2	FBX21_PONAB	-1.48729	0.000947	-1.58679	0.000732
TRINITY_DN99584_c2_g5	IAA17_ORYSJ^	-1.48733	0.000252	-2.40413	2.62E-13
TRINITY_DN101956_c5_g15	GLO5_ORYSJ^C	-1.48797	0.000495	-2.29724	4.12E-08
TRINITY_DN98400_c0_g2	IAA17_ORYSJ^	-1.4916	4.94E-06	-2.29931	3.96E-16
TRINITY_DN103167_c3_g8	14332_ORYSJ^	-1.49316	0.00042	-1.91506	4.59E-10
TRINITY_DN105333_c0_g5	IFRH_LUPAL^IF	-1.50324	0.000924	-2.52196	2.37E-09
TRINITY_DN103452_c4_g3	STR14_ARATH	-1.50686	3.48E-07	-2.52516	8.92E-25
TRINITY_DN95380_c1_g1	#N/A	-1.51182	0.000202	-2.65038	1.25E-12
TRINITY_DN107258_c4_g7	MDHG_ORYSJ^	-1.51236	4.21E-05	-1.7761	1.23E-08
TRINITY_DN103339_c0_g2	GPPL2_ARATH	-1.51247	0.000919	-2.13355	2E-07
TRINITY_DN99508_c1_g2	IPYR_ORYSJ^IP	-1.51892	1.15E-05	-2.36467	2.24E-16
TRINITY_DN94704_c0_g1	#N/A	-1.52653	0.000359	-1.46506	8.32E-05
TRINITY_DN100252_c1_g2	THF1_ORYSJ^T	-1.52683	2.72E-05	-1.68545	4.27E-09
TRINITY_DN95321_c2_g3	CUT1A_ARATH	-1.52856	1.65E-05	-1.31384	8.63E-05
TRINITY_DN112384_c6_g19	GLYM_SOLTU^	-1.52892	4.23E-08	-1.30519	3.42E-09
TRINITY_DN103542_c0_g1	YUGF_BACSU^	-1.53107	0.000229	-1.76953	8.97E-07
TRINITY_DN109428_c8_g6	CB121_HORVU	-1.53325	0.000553	-1.31462	0.000762
TRINITY_DN93184_c0_g1	ACP1_HORVU^	-1.53847	2.40E-05	-2.21549	1.33E-11
TRINITY_DN97303_c2_g1	STR14_ARATH	-1.53959	1.09E-05	-2.3986	5.2E-20
TRINITY_DN73946_c0_g1	SALT_ORYSJ^S	-1.54165	0.000368	-2.1476	8.28E-09
TRINITY_DN93728_c0_g2	ACP1_HORVU^	-1.54748	8.05E-05	-1.42346	2.07E-05
TRINITY_DN102022_c1_g1	THF1_ORYSJ^T	-1.54949	1.34E-06	-1.62803	2.95E-10
TRINITY_DN104472_c1_g1	PGMP_ARATH	-1.55117	3.91E-05	-1.7089	1.6E-07
TRINITY_DN97784_c0_g1	FK164_ARATH	-1.55305	0.000132	-1.70208	1.87E-06
TRINITY_DN98405_c1_g1	TPT_ORYSJ^TP	-1.56168	4.22E-06	-2.0492	1.32E-12
TRINITY_DN118743_c0_g2	G6PIP_ARATH^	-1.56225	0.000192	-1.19782	0.000553
TRINITY_DN115083_c0_g1	DPE2_ORYSJ^D	-1.57714	1.82E-07	-2.28902	7.95E-15

TRINITY_DN103542_c1_g2	YUGF_BACSU^	-1.58158	0.000487	-1.67958	1.42E-06
TRINITY_DN108926_c1_g3	COL2_ARATH^	-1.59136	0.000178	-1.3397	0.000385
TRINITY_DN107124_c2_g1	ATPG_MAIZE^	-1.59631	5.13E-05	-1.69594	1.61E-05
TRINITY_DN115469_c6_g53	FENR1_ORYSJ^	-1.60032	7.34E-11	-1.65153	1.87E-17
TRINITY_DN115469_c6_g65	FENR1_ORYSJ^	-1.60117	0.000234	-1.38483	6.96E-06
TRINITY_DN99853_c0_g1	CAS_MAIZE^C	-1.61256	0.000247	-2.13234	8.95E-08
TRINITY_DN103999_c8_g2	GLNA2_ORYSJ	-1.61355	1.21E-09	-2.35296	1.45E-14
TRINITY_DN111585_c10_g9	THI42_SORBI^	-1.61374	0.000399	-1.72971	2.16E-05
TRINITY_DN86231_c7_g1	CB121_HORVU	-1.61533	0.000218	-1.47128	6.03E-06
TRINITY_DN114254_c1_g2	PNSB2_ARATH	-1.61788	0.000706	-3.02553	1.26E-12
TRINITY_DN96540_c0_g4	CUT1A_ARATH	-1.61812	0.000666	-2.17131	2.74E-07
TRINITY_DN116122_c1_g3	CHUP1_ARATH	-1.61815	3.05E-08	-1.27966	2.79E-05
TRINITY_DN102867_c2_g1	PER50_ARATH	-1.62121	0.000398	-4.16257	2.92E-14
TRINITY_DN108385_c1_g3	PSAA_AGRST^	-1.62265	6.45E-06	-1.7183	2.77E-09
TRINITY_DN24264_c0_g1	BSPA_POPDE^	-1.62639	0.000116	-3.86108	6.07E-31
TRINITY_DN106153_c0_g3	DEGP8_ARATH	-1.62733	0.000814	-1.4047	0.000657
TRINITY_DN76179_c0_g2	#N/A	-1.62815	0.000245	-1.62672	1.18E-05
TRINITY_DN96074_c0_g1	CYT1_ORYSJ^C	-1.62838	2.87E-06	-1.02261	7.07E-05
TRINITY_DN65027_c1_g1	PSBR_HORVU^	-1.62957	0.000237	-2.13578	5.38E-07
TRINITY_DN110877_c7_g22	HPR1_ARATH^	-1.63042	3.32E-08	-1.63245	4.82E-12
TRINITY_DN91370_c3_g2	CP121_ARATH	-1.63202	0.000197	-1.66949	1.14E-05
TRINITY_DN110778_c2_g6	E138_ARATH^	-1.63521	3.00E-05	-1.21899	3.78E-05
TRINITY_DN87265_c4_g5	CP121_ARATH	-1.63601	2.08E-05	-2.34253	4.99E-14
TRINITY_DN101993_c1_g2	PNSB5_ARATH	-1.63855	1.36E-06	-1.22028	5.28E-05
TRINITY_DN103470_c9_g13	GLO1_ORYSJ^(	-1.63991	2.91E-05	-1.57601	1.02E-05
TRINITY_DN102377_c2_g5	ATP5H_ARATH	-1.64127	0.000808	-1.46561	0.00094
TRINITY_DN103999_c8_g1	GLNA2_ORYSJ	-1.64191	9.39E-06	-3.00718	2.14E-21
TRINITY_DN113085_c9_g47	THI42_SORBI^	-1.64447	0.000205	-1.73915	1.27E-05
TRINITY_DN101238_c1_g3	GPPL2_ARATH	-1.64711	1.17E-05	-2.13862	1.04E-11
TRINITY_DN119642_c1_g15	GLTB_ORYSJ^G	-1.66479	5.26E-06	-1.4672	2.77E-06
TRINITY_DN70609_c3_g3	PSBR_HORVU^	-1.66593	0.000296	-2.15836	2.55E-07
TRINITY_DN107506_c3_g2	CP41A_ARATH	-1.66766	1.19E-06	-3.06919	5.12E-19
TRINITY_DN107124_c4_g1	ATPG_MAIZE^	-1.66912	1.29E-08	-1.77789	1.33E-09
TRINITY_DN104659_c1_g6	BBD2_ORYSJ^B	-1.6709	7.97E-05	-2.20933	1.12E-08
TRINITY_DN104764_c1_g2	UP12_ORYSJ^U	-1.67102	1.96E-05	-2.64298	1.9E-14
TRINITY_DN107534_c0_g1	Y1480_ARATH	-1.67106	0.000178	-3.34946	2.42E-13
TRINITY_DN99867_c0_g1	PPL1_ARATH^	-1.6722	0.000965	-1.88473	1.32E-05
TRINITY_DN87463_c0_g1	PSAA_AGRST^	-1.68242	1.17E-05	-1.67794	1.14E-08
TRINITY_DN108426_c6_g1	SGAT_ARATH^	-1.68484	0.000762	-1.84804	1.9E-05
TRINITY_DN102034_c0_g1	TRXM_WHEAT	-1.69816	4.04E-05	-2.29253	3.24E-21
TRINITY_DN108240_c2_g3	PSBY_SPIOL^P	-1.69835	0.00019	-3.17388	5.51E-20
TRINITY_DN116305_c0_g1	DPE2_ORYSJ^D	-1.69994	7.86E-07	-1.90288	4.93E-11
TRINITY_DN117516_c6_g16	TKTC_MAIZE^T	-1.70415	0.000736	-1.69303	0.000456
TRINITY_DN115048_c1_g1	CP33_ARATH^	-1.7084	4.89E-05	-1.2624	0.000415
TRINITY_DN116867_c0_g1	DTX46_ARATH	-1.70885	3.67E-05	-1.36347	0.0003
TRINITY_DN119193_c1_g4	ALGC_PSEAE^	-1.71178	2.86E-07	-1.93642	3.33E-11
TRINITY_DN106237_c1_g1	G6PD1_ARATH	-1.71369	0.000275	-2.63896	2.38E-10

TRINITY_DN33873_c0_g2	BSPA_POPDE^	-1.71548	1.96E-06	-5.36302	1.39E-45
TRINITY_DN104836_c5_g3	GLNA2_HORVU	-1.7162	2.87E-05	-2.431	1.83E-14
TRINITY_DN100485_c0_g4	GLK1_ORYSJ^G	-1.71691	0.00018	-1.55708	0.000148
TRINITY_DN95522_c7_g2	#N/A	-1.71787	6.76E-05	-1.88507	3.57E-09
TRINITY_DN101431_c0_g1	PSAH_HORVU^	-1.72358	6.73E-07	-1.78389	3.43E-09
TRINITY_DN96540_c0_g3	CUT1A_ARATH	-1.72456	0.000173	-1.53808	0.000209
TRINITY_DN107506_c2_g4	CP41A_ARATH	-1.72496	5.05E-07	-3.25663	2.36E-22
TRINITY_DN108661_c4_g10	HPR1_ARATH^	-1.72781	1.64E-07	-2.1469	2.51E-14
TRINITY_DN104972_c0_g1	PGMP_ARATH	-1.73325	1.09E-06	-1.64121	4.37E-07
TRINITY_DN113085_c9_g40	THI42_MAIZE^	-1.73499	9.30E-05	-1.46074	0.000109
TRINITY_DN107986_c0_g1	#N/A	-1.73519	1.36E-05	-3.90335	2.23E-21
TRINITY_DN92658_c0_g1	PNSL1_ARATH	-1.7366	1.52E-07	-2.81285	5.51E-20
TRINITY_DN109819_c8_g16	FENR1_ORYSJ^	-1.73721	6.15E-06	-1.37222	0.000229
TRINITY_DN115425_c1_g16	RPE_ORYSJ^RP	-1.74274	1.43E-08	-1.54018	9.15E-11
TRINITY_DN91370_c3_g1	CP121_ARATH	-1.74545	7.44E-06	-2.60158	8.54E-14
TRINITY_DN111585_c10_g84	THI42_SORBI^	-1.74616	0.000237	-1.56628	1.03E-06
TRINITY_DN110475_c1_g6	S17P_WHEAT^	-1.75235	0.000895	-2.47583	3.74E-07
TRINITY_DN108661_c4_g28	HPR1_ARATH^	-1.75259	7.97E-07	-2.54034	5.62E-15
TRINITY_DN115981_c9_g191	TIP12_ORYSJ^	-1.7526	8.78E-05	-2.19366	4.56E-09
TRINITY_DN89925_c1_g1	#N/A	-1.75338	4.66E-07	-1.46257	4.62E-07
TRINITY_DN113096_c1_g13	YCF39_CYAPA^	-1.75573	0.000288	-2.60659	2.04E-08
TRINITY_DN95321_c2_g5	CUT1A_ARATH	-1.75672	8.01E-05	-1.6257	5.89E-07
TRINITY_DN113332_c1_g1	ATPZ_SYNP6^A	-1.75815	1.80E-07	-1.45712	1.37E-08
TRINITY_DN109142_c0_g4	GCST_SOLTU^	-1.7608	9.70E-06	-2.61414	3.86E-11
TRINITY_DN110909_c5_g19	PGKH_WHEAT	-1.76139	5.95E-06	-2.52305	3.44E-13
TRINITY_DN119606_c7_g7	GLTB_ORYSJ^G	-1.76438	0.000108	-1.44868	0.000188
TRINITY_DN116116_c0_g1	PHT1A_ORYSJ^	-1.76755	8.54E-07	-2.20744	5.35E-12
TRINITY_DN105688_c3_g1	#N/A	-1.76907	0.000156	-3.76578	7.32E-14
TRINITY_DN113021_c2_g4	MPK7_ORYSJ^	-1.76922	1.26E-05	-1.21049	0.000319
TRINITY_DN97276_c5_g4	#N/A	-1.7706	1.20E-06	-1.6443	2.36E-07
TRINITY_DN104606_c11_g14	GLO1_ORYSJ^	-1.77587	1.16E-06	-1.85048	3.47E-08
TRINITY_DN99244_c0_g2	HOL1_ARATH^	-1.77813	0.000491	-2.57952	1.1E-06
TRINITY_DN73228_c0_g3	#N/A	-1.7803	0.000225	-1.84657	6.13E-06
TRINITY_DN116376_c0_g1	PHT1A_ORYSJ^	-1.78334	3.99E-06	-2.09939	1.63E-08
TRINITY_DN114760_c1_g1	Y3471_ARATH	-1.78345	1.85E-05	-4.07219	7.55E-30
TRINITY_DN108023_c3_g3	LEA1_HORVU^	-1.79279	0.000152	-3.53163	1.56E-13
TRINITY_DN104606_c11_g2	GLO1_ORYSJ^	-1.79324	1.75E-08	-1.68414	5.86E-06
TRINITY_DN113332_c2_g4	ATPZ_SYNP6^A	-1.79908	5.83E-09	-1.67281	5.24E-11
TRINITY_DN113085_c9_g85	THI42_SORBI^	-1.80089	4.11E-05	-1.19831	0.00085
TRINITY_DN87023_c2_g2	#N/A	-1.80159	2.40E-07	-1.33981	1.83E-12
TRINITY_DN109121_c0_g2	AHL14_ARATH	-1.80433	4.58E-05	-1.94243	1.8E-06
TRINITY_DN97202_c0_g1	ARSB_DICDI^A	-1.81121	1.11E-05	-2.14294	5.19E-09
TRINITY_DN105810_c0_g3	VDE_ARATH^V	-1.81263	0.000117	-3.39845	2.64E-13
TRINITY_DN117305_c5_g8	TKTC_MAIZE^T	-1.813	6.43E-07	-1.37892	1.08E-05
TRINITY_DN106975_c1_g2	AB11G_ARATH	-1.81506	0.000102	-1.9512	8.25E-05
TRINITY_DN92704_c0_g4	PNSL1_ARATH	-1.8151	0.000249	-2.62392	1.81E-09
TRINITY_DN106688_c5_g2	MDHG_ORYSJ^	-1.81676	6.76E-08	-1.86036	8.73E-08

TRINITY_DN113419_c0_g2	PTR2_ARATH^	-1.82063	0.000478	-2.26791	2.14E-06
TRINITY_DN93795_c0_g1	FQR1_ARATH^	-1.82153	4.98E-05	-2.06637	4.3E-11
TRINITY_DN101794_c0_g2	ADF7_ORYSJ^A	-1.82448	2.15E-05	-1.39571	3.41E-05
TRINITY_DN98604_c0_g1	TPT_ORYSJ^TP	-1.82697	4.10E-08	-1.977	2.38E-16
TRINITY_DN109044_c2_g11	TPIS_ORYSJ^TI	-1.82713	0.000138	-1.54852	0.000213
TRINITY_DN98821_c7_g1	PSBO_WHEAT^	-1.82825	8.63E-10	-1.10934	0.000193
TRINITY_DN88286_c0_g1	#N/A	-1.83019	1.41E-05	-1.82601	1.12E-10
TRINITY_DN114224_c3_g7	CYSKP_SOLTU^	-1.83202	3.67E-05	-1.5721	7.11E-05
TRINITY_DN118362_c1_g5	OHK4_ORYSJ^(	-1.83619	0.000325	-1.39385	0.0009
TRINITY_DN98506_c0_g1	FK172_ARATH^	-1.83932	0.000909	-1.77395	0.000524
TRINITY_DN105468_c1_g1	TYPA_SYNY3^T	-1.83995	9.67E-11	-1.01269	5.37E-06
TRINITY_DN106211_c1_g1	LPA1_ARATH^I	-1.8417	2.99E-07	-1.04723	0.000469
TRINITY_DN119197_c0_g1	#N/A	-1.84304	0.000359	-2.58099	1.45E-07
TRINITY_DN86438_c4_g1	#N/A	-1.84344	1.42E-06	-1.1713	1.49E-07
TRINITY_DN109181_c2_g2	TIC62_PEA^TIC	-1.84709	2.79E-05	-3.09045	5.09E-12
TRINITY_DN104836_c5_g4	GLNA2_HORVU	-1.84919	1.38E-10	-2.39971	1.28E-29
TRINITY_DN107825_c0_g1	STR4_ARATH^S	-1.85066	7.50E-06	-2.09402	1.33E-08
TRINITY_DN111995_c9_g2	GLYM1_FLAPR	-1.85082	0.000121	-2.32351	1.58E-07
TRINITY_DN105715_c1_g8	ORR4_ORYSJ^(	-1.85098	5.38E-06	-1.63081	2.45E-05
TRINITY_DN118190_c0_g4	GPDL1_ARATH	-1.86064	0.000273	-2.51737	2.53E-07
TRINITY_DN108290_c0_g3	CCR1_ARATH^	-1.8611	3.48E-05	-1.61917	7.73E-05
TRINITY_DN104761_c8_g8	GGT1_ARATH^	-1.86142	3.96E-14	-1.80594	1.79E-22
TRINITY_DN86438_c4_g2	#N/A	-1.86176	4.09E-05	-1.75809	5.51E-07
TRINITY_DN90871_c0_g1	USPAL_ARATH	-1.8629	0.000315	-2.16325	5.02E-05
TRINITY_DN111792_c1_g5	RPE_SOLTU^RI	-1.86299	0.000128	-1.70355	8.3E-06
TRINITY_DN115753_c1_g2	PGP1B_ARATH	-1.86582	4.15E-06	-2.12149	1.24E-10
TRINITY_DN114843_c0_g3	F26_ARATH^F2	-1.8674	9.58E-05	-2.33366	1.8E-07
TRINITY_DN108426_c6_g5	SGAT_ARATH^	-1.87055	5.29E-05	-1.48403	0.000229
TRINITY_DN111056_c5_g19	PGKH_WHEAT^	-1.87397	5.03E-05	-2.43892	2.24E-15
TRINITY_DN109819_c8_g20	FENR1_ORYSJ^	-1.87829	3.33E-11	-2.08799	1.15E-23
TRINITY_DN86144_c5_g3	MT3B_ORYSJ^	-1.88013	4.06E-08	-1.14203	7.68E-06
TRINITY_DN102983_c0_g1	MPV17_DANR	-1.88237	0.000506	-1.91305	8.96E-05
TRINITY_DN104883_c1_g3	PRXQ_WHEAT^	-1.88242	5.60E-06	-1.33939	0.000131
TRINITY_DN113793_c0_g1	#N/A	-1.88308	0.000218	-1.68058	0.000281
TRINITY_DN112384_c6_g13	GLYM1_FLAPR	-1.88351	0.00017	-1.57722	0.000833
TRINITY_DN92943_c3_g1	PSBY_SPIOL^P:	-1.88444	1.44E-06	-3.27342	2.95E-20
TRINITY_DN96128_c0_g1	NFYC4_ARATH	-1.89315	0.000488	-2.20157	9.46E-07
TRINITY_DN107258_c4_g5	MDHG_ORYSJ^	-1.89478	9.32E-09	-1.70526	6.09E-09
TRINITY_DN111937_c8_g10	KPPR_WHEAT^	-1.89714	0.000216	-1.80948	5.9E-05
TRINITY_DN108503_c5_g10	F16P1_WHEAT	-1.89832	4.14E-05	-1.84903	1.08E-05
TRINITY_DN104490_c0_g1	CEEH1_CAEEL^	-1.90361	3.80E-05	-3.59426	2.02E-14
TRINITY_DN108610_c12_g21	SGAT_ARATH^	-1.9045	1.69E-06	-1.81869	2.66E-08
TRINITY_DN114576_c1_g1	SBT35_ARATH^	-1.90699	1.48E-05	-2.46658	1.93E-12
TRINITY_DN108815_c4_g4	CONS_ARATH^	-1.90923	0.000118	-1.80451	2.18E-05
TRINITY_DN95615_c0_g2	#N/A	-1.9103	6.10E-07	-2.4966	2.48E-09
TRINITY_DN107248_c0_g2	ZIP7_ORYSJ^ZI	-1.91116	7.20E-06	-2.6757	5.28E-10
TRINITY_DN105135_c11_g1	GGT1_ARATH^	-1.91385	4.66E-13	-1.77222	2.4E-11

TRINITY_DN104761_c8_g4	GGT1_ARATH^	-1.91703	1.28E-09	-1.69118	1.24E-06
TRINITY_DN113685_c0_g2	PLGG1_ARATH	-1.91742	3.96E-05	-2.00626	6.64E-06
TRINITY_DN100047_c0_g2	Y2766_ARATH	-1.91756	0.000555	-2.35863	0.000384
TRINITY_DN95373_c0_g2	ARSB_DICDI^A	-1.91968	0.000126	-2.311	3.8E-07
TRINITY_DN115556_c1_g12	MPK8_ORYSJ^	-1.92293	0.00095	-1.79409	0.000226
TRINITY_DN91695_c0_g2	#N/A	-1.92356	1.31E-05	-3.93118	3.04E-16
TRINITY_DN95281_c1_g6	IMCE_ORYSJ^I	-1.92646	8.81E-06	-2.18205	3.67E-09
TRINITY_DN95701_c1_g1	SSL10_ARATH^	-1.92935	3.62E-05	-2.78959	4.85E-10
TRINITY_DN114100_c2_g4	SBT35_ARATH	-1.93406	4.27E-05	-2.44499	1.57E-09
TRINITY_DN112985_c0_g3	PP281_ARATH	-1.93465	0.000858	-1.91118	0.000295
TRINITY_DN119689_c2_g1	#N/A	-1.93482	0.000238	-3.01579	2.44E-08
TRINITY_DN104887_c1_g12	UP12_ORYSJ^U	-1.93583	1.39E-06	-2.74384	1.76E-15
TRINITY_DN99469_c7_g1	PSBO_WHEAT^	-1.93961	1.02E-11	-1.31416	2.29E-06
TRINITY_DN114843_c0_g5	F26_ARATH^F2	-1.95151	0.000197	-2.25859	3.22E-06
TRINITY_DN99811_c0_g1	ARSB_DICDI^A	-1.95244	9.64E-06	-2.45273	5.04E-10
TRINITY_DN108610_c12_g11	SGAT_ARATH^	-1.95427	1.88E-06	-1.62544	2.49E-05
TRINITY_DN117516_c6_g27	TKTC_MAIZE^T	-1.95812	3.18E-09	-1.61536	4.65E-10
TRINITY_DN108121_c0_g9	PPH_ARATH^P	-1.96035	2.53E-05	-1.77505	9.74E-06
TRINITY_DN110877_c7_g21	HPR1_ARATH^	-1.96325	3.66E-07	-2.3032	3.47E-11
TRINITY_DN100903_c0_g1	TL29_SOLLC^T	-1.96438	4.37E-07	-2.22761	1.18E-10
TRINITY_DN105158_c1_g5	FER_WHEAT^F	-1.96489	1.38E-09	-3.38394	1.92E-34
TRINITY_DN106757_c1_g5	RUBR_SYNY3^	-1.96618	1.19E-06	-2.91761	9.16E-15
TRINITY_DN104069_c1_g2	PDX2_ARATH^	-1.97078	1.56E-05	-1.6092	0.000211
TRINITY_DN96544_c0_g1	LHCA5_ARATH	-1.97097	7.59E-05	-1.7455	1.35E-05
TRINITY_DN110596_c0_g1	TAR1_ARATH^	-1.97154	0.000607	-3.29766	1.91E-10
TRINITY_DN103470_c9_g2	GLO1_ORYSJ^G	-1.97309	5.57E-14	-2.04282	4.9E-10
TRINITY_DN108426_c6_g11	SGAT_ARATH^	-1.97558	6.72E-06	-2.01117	1.22E-07
TRINITY_DN106579_c4_g1	PTR22_ARATH	-1.97994	0.000371	-3.1713	3.74E-08
TRINITY_DN107453_c0_g2	ASPG1_ARATH	-1.9809	0.000999	-2.4278	5.74E-06
TRINITY_DN89661_c3_g2	PSAN_HORVU^	-1.98206	5.64E-08	-2.97476	8.65E-23
TRINITY_DN98126_c1_g2	LEA1_HORVU^	-1.98374	0.000158	-3.1988	8.74E-13
TRINITY_DN108661_c4_g48	#N/A	-1.98595	0.000104	-2.07257	7.48E-06
TRINITY_DN111585_c10_g46	THI42_MAIZE^	-1.98645	3.96E-07	-1.31616	9.42E-05
TRINITY_DN106723_c2_g6	RGA4_SOLBU^	-1.98674	2.38E-05	-4.2038	6.78E-16
TRINITY_DN117536_c0_g1	Y5738_ARATH	-1.98707	0.000578	-2.53465	1.38E-05
TRINITY_DN111648_c2_g5	Y5129_ARATH	-1.98754	0.000346	-3.67051	2.62E-09
TRINITY_DN108545_c1_g2	CYSKP_SOLTU^	-1.99934	1.26E-06	-1.76756	5.92E-09
TRINITY_DN107205_c2_g1	TYPA_SYNY3^T	-1.99994	4.93E-14	-1.15253	4.86E-08
TRINITY_DN85990_c1_g1	CFI3_ARATH^C	-2.0028	4.42E-05	-2.15869	3.55E-09
TRINITY_DN85374_c6_g1	PSBR_SOLLC^P	-2.0029	0.00051	-1.67298	2.83E-05
TRINITY_DN91822_c1_g2	IMCE_ORYSJ^I	-2.00318	5.00E-07	-2.24322	2.22E-10
TRINITY_DN113714_c6_g1	F26_ARATH^F2	-2.00815	9.19E-09	-2.14512	1.26E-10
TRINITY_DN113085_c9_g126	THI42_SORBI^	-2.00854	1.58E-05	-1.22552	0.000368
TRINITY_DN103740_c1_g1	ALFC_ORYSJ^A	-2.01055	0.000621	-3.06172	1.11E-06
TRINITY_DN111792_c1_g12	RPE_ORYSJ^RP	-2.01327	1.38E-09	-1.51683	1.26E-09
TRINITY_DN111904_c0_g1	SPPA1_ARATH	-2.01576	0.00014	-2.33444	1.27E-06
TRINITY_DN113085_c9_g100	THI42_MAIZE^	-2.01587	0.000836	-1.92155	2.14E-06

TRINITY_DN97784_c1_g1	FK164_ARATH	-2.01885	0.000606	-1.83281	0.000126
TRINITY_DN118441_c2_g92	TIP12_ORYSJ^	-2.02121	4.17E-05	-2.11177	1.41E-06
TRINITY_DN108007_c0_g29	GPPL1_ARATH	-2.02518	0.000143	-2.80443	7.93E-08
TRINITY_DN108881_c1_g1	GCST_FLAPR^	-2.02539	7.62E-09	-2.60655	2.92E-18
TRINITY_DN101431_c1_g1	PSAH_HORVU	-2.03009	2.18E-08	-1.90572	3.25E-14
TRINITY_DN105983_c0_g1	Y3720_ARATH	-2.0334	0.000141	-4.58616	4.35E-14
TRINITY_DN101040_c0_g4	CRK20_ARATH	-2.03948	2.75E-05	-3.10483	1.13E-10
TRINITY_DN100136_c0_g8	HLIP_ARATH^H	-2.04104	1.03E-05	-2.12397	1.51E-07
TRINITY_DN102022_c1_g2	THF1_ORYSJ^T	-2.04466	4.92E-05	-1.51415	0.000466
TRINITY_DN91627_c1_g1	#N/A	-2.04956	1.16E-07	-1.72356	2.92E-08
TRINITY_DN109075_c3_g22	F16P1_WHEAT	-2.05227	0.000333	-3.45478	4.24E-09
TRINITY_DN103710_c2_g6	PSB28_ORYSJ^	-2.05236	0.000118	-1.87997	0.000183
TRINITY_DN108081_c1_g10	LPA3_ARATH^	-2.05365	4.37E-06	-3.75723	2.14E-15
TRINITY_DN97737_c0_g1	HHL1_ARATH^	-2.05479	2.04E-07	-1.37654	2.91E-06
TRINITY_DN92259_c1_g3	#N/A	-2.06238	1.96E-05	-3.61472	1.57E-16
TRINITY_DN106542_c2_g1	PTA16_ARATH	-2.067	1.84E-05	-4.64994	2.13E-21
TRINITY_DN102502_c2_g1	AAPC_CENCI^	-2.06958	2.19E-06	-1.63728	3.69E-07
TRINITY_DN109221_c1_g4	AKRCA_ARATH	-2.07283	2.88E-06	-2.75649	4.31E-11
TRINITY_DN143771_c0_g1	ACP1_HORVU	-2.07463	1.19E-06	-2.32351	2.97E-06
TRINITY_DN103409_c0_g1	ABHD6_MOUS	-2.07502	0.000516	-3.32919	1.71E-07
TRINITY_DN116433_c3_g10	THIC_ARATH^T	-2.0813	1.12E-12	-2.34282	4.41E-33
TRINITY_DN111929_c8_g23	KPPR_WHEAT	-2.08208	2.29E-12	-2.95353	8.84E-29
TRINITY_DN93728_c0_g1	ACP1_HORVU	-2.08497	2.04E-07	-2.32021	7.71E-11
TRINITY_DN95779_c0_g1	GUN4C_ARATH	-2.08726	7.67E-05	-1.74898	0.000706
TRINITY_DN109321_c6_g7	PNSB4_ARATH	-2.09278	4.96E-08	-2.49006	1.24E-11
TRINITY_DN105376_c0_g1	APR1_ORYSJ^	-2.09297	5.91E-07	-1.97232	2.8E-05
TRINITY_DN108426_c6_g17	SGAT_ARATH^	-2.09624	5.38E-11	-1.96922	3.95E-12
TRINITY_DN100475_c0_g1	TL29_SOLLC^T	-2.09858	1.57E-06	-2.09102	2.69E-07
TRINITY_DN110274_c0_g2	#N/A	-2.0992	0.000277	-2.19831	7.88E-06
TRINITY_DN102215_c3_g3	UCRIA_WHEAT	-2.10402	2.43E-08	-1.39759	7.67E-06
TRINITY_DN25219_c0_g1	PPD3_ARATH^	-2.10466	5.60E-05	-2.07639	8.21E-06
TRINITY_DN113203_c3_g1	Y3475_ARATH	-2.10723	5.81E-08	-4.60873	1.17E-32
TRINITY_DN108023_c3_g2	LEA3_ORYSJ^L	-2.11014	0.000327	-4.40631	6.42E-13
TRINITY_DN102558_c0_g1	Y3913_ARATH	-2.11248	5.38E-06	-2.78109	1.2E-09
TRINITY_DN101264_c0_g9	#N/A	-2.11365	2.88E-05	-3.04258	5.1E-10
TRINITY_DN112915_c2_g1	NIK2_ARATH^	-2.12052	3.28E-05	-4.04718	5.92E-13
TRINITY_DN3282_c0_g1	RBS3_WHEAT	-2.12382	2.52E-06	-6.28534	4.43E-21
TRINITY_DN107032_c1_g3	CYP38_ARATH	-2.12389	0.000106	-2.20834	5.74E-06
TRINITY_DN107512_c5_g1	ZIP7_ORYSJ^ZI	-2.12433	1.03E-06	-3.45569	2.21E-14
TRINITY_DN107032_c0_g1	CYP38_ARATH	-2.12686	1.13E-05	-2.24274	5.64E-09
TRINITY_DN93698_c1_g2	HHL1_ARATH^	-2.12786	2.67E-06	-1.28087	0.000833
TRINITY_DN110180_c4_g1	PPD6_ARATH^	-2.12808	9.11E-06	-2.03945	3.47E-05
TRINITY_DN114679_c0_g1	RCC2_MOUSE	-2.12831	2.59E-05	-1.66192	0.000157
TRINITY_DN91215_c0_g1	#N/A	-2.13745	7.28E-09	-1.77082	2.37E-10
TRINITY_DN114794_c0_g5	ERS2_ORYSJ^E	-2.13945	1.36E-05	-1.71324	1.94E-05
TRINITY_DN114442_c4_g2	DAR2_ARATH^	-2.13973	0.000469	-3.00665	4.43E-09
TRINITY_DN93330_c0_g3	#N/A	-2.141	5.42E-06	-2.00708	1.16E-06

TRINITY_DN102191_c0_g2	AAPC_CENCI^/	-2.14128	2.83E-09	-1.5156	6.16E-07
TRINITY_DN98677_c1_g1	#N/A	-2.14308	0.000546	-3.33241	1.81E-07
TRINITY_DN103470_c9_g9	GLO1_ORYSJ^(	-2.14782	7.87E-09	-1.94559	2.57E-05
TRINITY_DN111929_c8_g5	KPPR_WHEAT^	-2.14841	2.21E-09	-3.10781	1.68E-23
TRINITY_DN108404_c0_g1	RPI2_ARATH^F	-2.14902	0.000671	-1.96494	0.00016
TRINITY_DN101264_c0_g3	#N/A	-2.15142	0.000122	-3.46317	2.42E-13
TRINITY_DN95391_c2_g1	CB23_ORYSJ^C	-2.15303	0.000534	-2.31617	0.000157
TRINITY_DN117449_c1_g1	ALN_ORYSJ^AL	-2.1556	0.000231	-2.66173	6.62E-07
TRINITY_DN5444_c0_g1	GLO5_ORYSJ^(	-2.15727	0.000711	-3.22959	5.21E-07
TRINITY_DN113096_c2_g21	YCF39_CYAPA^	-2.1578	1.28E-05	-3.88377	6.79E-13
TRINITY_DN100401_c1_g2	Y2766_ARATH-	-2.16132	1.33E-06	-1.84056	7.01E-08
TRINITY_DN115760_c0_g2	CRK25_ARATH	-2.16154	0.000912	-2.49836	5.88E-05
TRINITY_DN108610_c12_g13	SGAT_ARATH^	-2.16176	0.000145	-2.05331	2.06E-05
TRINITY_DN112517_c7_g10	G3PB_ARATH^	-2.16637	5.00E-06	-2.14646	7.04E-07
TRINITY_DN31523_c0_g1	RBS3_WHEAT^	-2.17068	0.000729	-4.06892	8.84E-11
TRINITY_DN102613_c2_g1	GLYK_ARATH^	-2.17141	6.93E-08	-1.99467	6.93E-09
TRINITY_DN98817_c0_g2	P2C05_ORYSJ^	-2.17243	2.41E-07	-1.15105	0.000768
TRINITY_DN114409_c1_g1	GPX4_SPIOL^G	-2.17769	1.66E-07	-2.01678	6.49E-08
TRINITY_DN103995_c0_g1	#N/A	-2.17962	1.71E-05	-1.88821	3.81E-05
TRINITY_DN20396_c0_g2	PSBM_AGRST^	-2.18104	0.000298	-2.4959	1.39E-05
TRINITY_DN108503_c5_g16	F16P1_WHEAT	-2.18299	2.22E-09	-2.27389	8.53E-12
TRINITY_DN108503_c5_g18	F16P1_WHEAT	-2.18601	4.78E-07	-2.06211	1.55E-07
TRINITY_DN108545_c1_g4	CYSKP_SOLTU^	-2.18614	7.78E-08	-1.72208	3.49E-07
TRINITY_DN108472_c0_g2	STR4_ARATH^!	-2.18827	8.82E-10	-2.14886	1.17E-12
TRINITY_DN96128_c1_g2	NFYC4_ARATH	-2.19223	2.62E-05	-2.72944	7.02E-11
TRINITY_DN111783_c0_g1	ASPG1_ARATH	-2.19453	9.57E-06	-2.47952	4.8E-08
TRINITY_DN112517_c7_g61	G3PB_PEA^G3	-2.1952	1.03E-06	-2.01673	3.46E-07
TRINITY_DN115720_c2_g1	RPI3_ARATH^F	-2.19954	2.40E-10	-2.05077	5.95E-13
TRINITY_DN88718_c1_g3	TRY_ARATH^TI	-2.20032	5.37E-05	-3.97684	3.25E-12
TRINITY_DN100900_c1_g1	CTNS_ARATH^	-2.20455	1.29E-05	-1.92385	2.16E-05
TRINITY_DN107258_c4_g8	MDHG_ORYSJ^	-2.20529	1.40E-05	-1.67766	5.91E-05
TRINITY_DN101142_c0_g1	MPV17_DANR	-2.20695	7.58E-05	-2.76174	1.41E-06
TRINITY_DN101956_c5_g8	GLO5_ORYSJ^(	-2.21513	0.00082	-4.46011	1.34E-07
TRINITY_DN91622_c0_g1	OMT3_SORBI^	-2.21542	0.000134	-3.06475	2.3E-08
TRINITY_DN108732_c3_g12	DTC_ARATH^D	-2.21573	2.08E-05	-3.92258	1.04E-12
TRINITY_DN88013_c0_g1	#N/A	-2.21676	5.39E-05	-5.45602	4.55E-12
TRINITY_DN108426_c6_g15	SGAT_ARATH^	-2.21807	0.000295	-1.88951	0.000406
TRINITY_DN116433_c4_g14	THIC_ARATH^T	-2.22116	2.83E-14	-2.3632	1E-20
TRINITY_DN98821_c7_g3	PSBO_WHEAT^	-2.22137	0.00012	-2.10336	0.000318
TRINITY_DN109245_c1_g5	GDL83_ARATH	-2.22277	0.000853	-3.57355	8.28E-07
TRINITY_DN113233_c0_g1	PP186_ARATH	-2.22505	5.61E-05	-3.26837	9.77E-09
TRINITY_DN103854_c0_g1	GLGL1_ARATH	-2.22769	5.85E-11	-1.18284	4.38E-06
TRINITY_DN115891_c0_g1	VIP1_DROME^	-2.23178	4.59E-06	-2.03233	1E-05
TRINITY_DN104887_c1_g15	UP12_ORYSJ^U	-2.23215	6.08E-06	-3.03052	2.41E-11
TRINITY_DN102191_c0_g1	AAPC_CENCI^/	-2.23869	5.14E-07	-1.7099	2.6E-06
TRINITY_DN108610_c12_g18	SGAT_ARATH^	-2.24568	2.29E-09	-2.13935	1.99E-19
TRINITY_DN108661_c4_g27	DHGY_CUCSA^	-2.24704	6.83E-06	-2.78476	2.49E-08

TRINITY_DN103300_c1_g4	#N/A	-2.24789	0.000417	-2.87162	4.71E-06
TRINITY_DN107884_c1_g10	ALF1_PEA^ALF	-2.24808	2.24E-06	-2.12246	5.97E-05
TRINITY_DN106014_c0_g1	ASE1_ARATH^	-2.24999	1.40E-05	-2.19427	6.85E-06
TRINITY_DN97946_c4_g1	TI110_PEA^TI1	-2.25231	1.91E-07	-5.22119	1.49E-37
TRINITY_DN108809_c3_g1	PUM7_ARATH	-2.26371	3.91E-06	-2.67126	3.4E-11
TRINITY_DN102082_c3_g1	UCRIA_WHEAT	-2.26443	3.96E-14	-1.55209	8.17E-10
TRINITY_DN111937_c8_g19	KPPR_WHEAT	-2.26969	1.51E-07	-2.56767	2.82E-11
TRINITY_DN113085_c9_g27	THI42_VITVI^T	-2.27044	1.07E-05	-1.7576	1.17E-05
TRINITY_DN95391_c2_g3	CB23_ORYSJ^C	-2.27143	9.98E-07	-2.00475	1.05E-05
TRINITY_DN95373_c0_g1	ARSB_DICDI^A	-2.27161	3.23E-06	-2.59167	7.09E-09
TRINITY_DN114630_c1_g1	PHSH_WHEAT	-2.27248	1.43E-12	-2.32722	2.42E-21
TRINITY_DN102215_c3_g2	UCRIA_WHEAT	-2.28358	3.73E-16	-1.42222	1.27E-06
TRINITY_DN112517_c7_g85	G3PA_SINAL^G	-2.28398	5.30E-06	-2.36083	2.71E-07
TRINITY_DN105117_c0_g1	PAP7_ARATH^	-2.29129	2.17E-05	-2.79036	1.3E-08
TRINITY_DN89720_c3_g1	GCSH_ORYSJ^	-2.29963	1.01E-12	-3.15944	6.16E-30
TRINITY_DN116423_c3_g1	PNSB4_ARATH	-2.30307	9.03E-12	-2.52692	2.04E-16
TRINITY_DN110274_c1_g3	#N/A	-2.30616	6.38E-05	-2.62788	1.23E-06
TRINITY_DN95697_c0_g3	#N/A	-2.30738	8.45E-05	-1.69242	0.0006
TRINITY_DN116555_c0_g4	Y2734_MYCBC	-2.30931	1.52E-05	-2.02605	1.17E-05
TRINITY_DN117628_c13_g163	RBS3_WHEAT^	-2.31167	1.80E-09	-3.99239	1.6E-20
TRINITY_DN108610_c12_g19	SGAT_ARATH^	-2.31419	4.90E-09	-1.95803	2.98E-07
TRINITY_DN91018_c0_g1	#N/A	-2.31504	1.25E-10	-1.94248	6.59E-13
TRINITY_DN106272_c1_g5	SPX5_ORYSJ^S	-2.3199	8.13E-06	-3.17418	2.45E-09
TRINITY_DN109075_c3_g23	F16P1_WHEAT	-2.32377	5.50E-10	-2.22212	1.14E-16
TRINITY_DN88983_c0_g5	RBS3_WHEAT^	-2.32543	0.000324	-3.9393	6.45E-09
TRINITY_DN117427_c0_g4	Y5738_ARATH	-2.32999	1.59E-07	-3.77941	3.08E-17
TRINITY_DN108426_c6_g20	SGAT_ARATH^	-2.33123	3.00E-09	-1.71396	4.94E-09
TRINITY_DN108921_c0_g1	PIN6_ORYSJ^P	-2.33133	2.64E-10	-2.9475	3.7E-12
TRINITY_DN111937_c8_g31	KPPR_WHEAT	-2.33425	1.31E-16	-3.16289	1.08E-22
TRINITY_DN114672_c0_g16	ATPD_SORBI^	-2.33717	1.55E-11	-2.32008	2.78E-14
TRINITY_DN108893_c0_g1	MAX1_ARATH	-2.34081	6.34E-05	-3.38498	1.15E-08
TRINITY_DN117482_c1_g1	Y637_CHLL7^Y	-2.34448	1.21E-07	-4.03076	5.85E-19
TRINITY_DN64079_c2_g6	RBS1_WHEAT^	-2.34819	4.99E-09	-2.65443	2.89E-13
TRINITY_DN101496_c0_g2	C71E1_SORBI^	-2.35317	5.55E-05	-2.98932	1.95E-07
TRINITY_DN105956_c1_g3	Y4554_ARATH	-2.35611	1.07E-05	-1.95101	7.11E-05
TRINITY_DN99441_c0_g1	STEP1_ARATH	-2.35663	1.85E-05	-2.69086	5.97E-07
TRINITY_DN112554_c1_g3	DHLA_XANFL^	-2.35721	5.99E-05	-2.7272	1.55E-06
TRINITY_DN101264_c0_g2	#N/A	-2.35915	6.97E-06	-2.80285	1.16E-10
TRINITY_DN101120_c0_g2	ZIP1_ARATH^Z	-2.36357	6.17E-06	-2.42824	2.72E-07
TRINITY_DN96943_c0_g1	LHCA5_ARATH	-2.36372	3.62E-07	-1.85529	4.49E-06
TRINITY_DN111056_c5_g20	PGKH_WHEAT	-2.36646	1.61E-09	-2.71833	1.83E-13
TRINITY_DN112730_c0_g3	CRK29_ARATH	-2.36704	3.84E-06	-2.06985	1.87E-06
TRINITY_DN101040_c0_g1	CRK20_ARATH	-2.36727	2.71E-06	-2.41649	1.21E-07
TRINITY_DN118447_c0_g1	DJ1B_ARATH^I	-2.37092	3.14E-08	-2.75861	1.71E-12
TRINITY_DN20396_c0_g1	PSBM_AGRST^	-2.37284	0.000322	-2.28309	1.51E-05
TRINITY_DN113085_c9_g20	THI42_SORBI^	-2.37433	3.81E-06	-1.78562	7.11E-05
TRINITY_DN20589_c0_g1	OMT2_SORBI^	-2.38072	3.91E-05	-3.07538	1.33E-08

TRINITY_DN94756_c1_g1	PSAG_HORVU^	-2.38092	3.49E-07	-3.55003	2.89E-13
TRINITY_DN107032_c1_g2	CYP38_ARATH	-2.38125	0.000392	-2.38989	5.21E-05
TRINITY_DN105576_c0_g3	GLGL1_ARATH	-2.38277	4.76E-12	-1.31526	1.41E-06
TRINITY_DN69818_c0_g1	ORR1_ORYSJ^(	-2.38776	0.00017	-2.96995	8.32E-07
TRINITY_DN99616_c0_g1	#N/A	-2.3904	8.68E-08	-4.25917	2.12E-17
TRINITY_DN74135_c0_g1	PSBM_AGRST^	-2.39436	0.00022	-2.23251	2.67E-05
TRINITY_DN112000_c2_g1	NFD4_ARATH^	-2.39442	0.000164	-2.668	1.92E-05
TRINITY_DN90947_c0_g1	NDHM_ORYSJ^	-2.39557	0.000267	-3.02925	7.65E-06
TRINITY_DN114264_c0_g4	CPNB4_ARATH	-2.3981	1.94E-06	-2.65776	1E-08
TRINITY_DN109982_c1_g1	DAAA_ARATH^	-2.40045	0.000222	-4.09639	7.06E-09
TRINITY_DN117536_c0_g2	Y5738_ARATH	-2.40134	5.60E-08	-3.56106	8.27E-18
TRINITY_DN103740_c0_g5	ALFC_ORYSJ^A	-2.40411	1.00E-09	-2.72779	7.18E-22
TRINITY_DN90086_c0_g1	#N/A	-2.41473	2.69E-09	-2.01446	1.36E-12
TRINITY_DN97349_c2_g2	PSBQ1_MAIZE	-2.41505	8.04E-10	-3.85652	2.13E-21
TRINITY_DN113454_c0_g1	PHSL_IPOBA^F	-2.41672	1.71E-09	-1.12662	0.000925
TRINITY_DN117628_c13_g119	RBS2_ORYSJ^R	-2.41998	9.46E-07	-6.82059	1.62E-22
TRINITY_DN98688_c1_g2	D27_ORYSJ^D2	-2.42078	1.64E-07	-2.40261	6.39E-09
TRINITY_DN113519_c2_g6	KCS10_ARATH	-2.42337	0.000322	-2.07062	0.000301
TRINITY_DN101231_c0_g1	CML29_ORYSJ	-2.42559	1.47E-08	-3.35718	1.76E-20
TRINITY_DN104400_c1_g2	AGAL_ORYSJ^A	-2.43166	1.19E-09	-2.98	4.67E-12
TRINITY_DN107884_c1_g12	ALF1_PEA^ALF	-2.43347	1.69E-11	-3.74229	3.92E-26
TRINITY_DN92131_c0_g1	UBC5A_ORYSJ	-2.4368	4.65E-07	-2.54792	2.22E-09
TRINITY_DN118524_c0_g1	ATD1A_DANRE	-2.43718	4.15E-05	-4.29756	1.29E-10
TRINITY_DN117628_c13_g152	RBS3_WHEAT^	-2.44089	9.77E-08	-4.27703	5.96E-18
TRINITY_DN102902_c0_g4	CCDA1_ORYSJ	-2.44229	1.51E-09	-1.53878	1.13E-06
TRINITY_DN87023_c2_g3	#N/A	-2.44232	5.95E-10	-1.98295	3.83E-12
TRINITY_DN92998_c0_g3	NCS2_PAPSO^	-2.44531	2.59E-05	-3.69323	2.94E-09
TRINITY_DN109110_c0_g1	ATCA1_ARATH	-2.4469	0.000126	-3.41785	2.08E-07
TRINITY_DN102562_c2_g1	APR1_ORYSJ^A	-2.44704	3.39E-08	-2.05825	2.11E-10
TRINITY_DN100572_c0_g1	DYR_SCHPO^D	-2.44738	0.00025	-4.90819	3.81E-09
TRINITY_DN117628_c13_g287	RBS1_WHEAT^	-2.45052	0.000363	-6.69189	3.17E-09
TRINITY_DN99452_c4_g16	CAHC_HORVU	-2.45076	1.03E-08	-2.9163	3.23E-12
TRINITY_DN60280_c0_g1	ZSS1_ZINZE^ZS	-2.45774	6.30E-05	-7.35088	1.79E-22
TRINITY_DN109080_c1_g6	NDHK_AGRST^	-2.45975	0.000608	-3.0762	4.38E-06
TRINITY_DN82751_c0_g3	G3PB_ARATH^	-2.46605	0.00043	-2.27868	8.69E-05
TRINITY_DN105810_c1_g1	VDE_ARATH^V	-2.47459	2.36E-08	-2.75901	2.9E-12
TRINITY_DN104137_c0_g2	LSD1_ORYSJ^L	-2.47582	3.93E-06	-2.67912	9.74E-08
TRINITY_DN98681_c0_g4	SWT3A_ORYSJ	-2.47971	0.000544	-3.12132	2.51E-06
TRINITY_DN111056_c5_g21	PGKH_WHEAT	-2.48117	6.88E-06	-2.86931	1.47E-07
TRINITY_DN110475_c1_g8	S17P_WHEAT^	-2.48155	5.48E-08	-2.56936	2.96E-10
TRINITY_DN88310_c1_g1	GCSH_ORYSJ^(	-2.48664	3.54E-15	-3.19579	6.03E-37
TRINITY_DN99452_c4_g20	CAHC_HORVU	-2.48723	7.81E-11	-3.00897	5.52E-18
TRINITY_DN110467_c13_g22	G3PA2_ARATH	-2.49147	9.19E-09	-2.72683	1.24E-12
TRINITY_DN108610_c12_g17	SGAT_ARATH^	-2.49187	0.000168	-3.6078	8.32E-08
TRINITY_DN110692_c2_g6	S17P_WHEAT^	-2.4955	6.41E-13	-2.9388	4.04E-26
TRINITY_DN115044_c1_g10	IRT1_ORYSJ^IR	-2.49875	7.14E-07	-3.5621	1.24E-12
TRINITY_DN106542_c1_g1	PTA16_ARATH	-2.50006	2.26E-12	-4.91134	5.33E-32

TRINITY_DN105648_c0_g1	CYP37_ARATH	-2.50688	9.92E-06	-2.22559	8.48E-06
TRINITY_DN97468_c3_g1	CUT1B_ARATH	-2.51609	1.29E-11	-2.87352	1.06E-16
TRINITY_DN111937_c8_g25	KPPR_WHEAT^	-2.52296	3.21E-07	-3.77397	6.21E-13
TRINITY_DN110475_c1_g1	S17P_WHEAT^	-2.52389	2.24E-18	-2.6225	5.66E-18
TRINITY_DN107219_c0_g1	#N/A	-2.52556	4.23E-08	-2.41799	6.26E-10
TRINITY_DN100098_c1_g2	PPL1_ARATH^I	-2.52573	1.90E-06	-2.49922	4.54E-08
TRINITY_DN109357_c3_g16	CP41B_ARATH	-2.52593	0.000736	-2.41125	0.000296
TRINITY_DN101228_c1_g1	ARSB_DICDI^A	-2.53227	7.87E-09	-2.31771	1.8E-09
TRINITY_DN105048_c7_g2	PRSP1_SPIOL^	-2.53781	6.19E-06	-2.86765	1.9E-08
TRINITY_DN113765_c1_g5	ABHD3_HUMA	-2.54284	1.55E-05	-2.77061	9.76E-07
TRINITY_DN99627_c0_g1	Y2766_ARATH	-2.54481	4.67E-09	-2.24017	6.62E-11
TRINITY_DN114424_c1_g1	PHSH_WHEAT^	-2.55093	1.53E-10	-2.10163	9.85E-14
TRINITY_DN110909_c5_g3	PGKH_WHEAT	-2.55658	4.57E-11	-2.27753	5.22E-11
TRINITY_DN112517_c7_g7	G3PB_ARATH^	-2.56012	1.81E-05	-2.52698	2.57E-06
TRINITY_DN64079_c2_g10	RBS1_WHEAT^	-2.56039	3.11E-06	-4.94348	2.64E-13
TRINITY_DN95963_c0_g1	C7D55_HYOMI	-2.56705	7.87E-09	-1.92258	2E-06
TRINITY_DN98573_c0_g2	NU5C_AGRST^	-2.56964	2.29E-15	-2.68225	2.2E-17
TRINITY_DN94109_c0_g1	RPI3_ARATH^F	-2.57263	1.37E-11	-2.28654	3.42E-13
TRINITY_DN117628_c13_g130	RBS2_WHEAT^	-2.57858	4.39E-05	-3.09799	4.98E-07
TRINITY_DN102892_c3_g1	F16P2_SACHY^	-2.58069	8.18E-16	-3.54354	2.17E-32
TRINITY_DN89087_c0_g1	#N/A	-2.58192	2.93E-11	-3.6239	1.79E-23
TRINITY_DN110166_c1_g2	IPYR4_ARATH^	-2.58272	6.38E-06	-2.88076	5.49E-08
TRINITY_DN112554_c2_g13	DHLA_XANFL^	-2.58288	2.09E-05	-1.8023	0.000923
TRINITY_DN110467_c12_g1	G3PA_MAIZE^	-2.58512	4.92E-10	-2.91016	2.5E-20
TRINITY_DN101055_c1_g4	#N/A	-2.58621	5.30E-08	-1.49723	6.32E-05
TRINITY_DN107140_c0_g9	TRL31_ORYSJ^	-2.58797	0.000421	-3.2056	2.55E-05
TRINITY_DN25345_c0_g1	RBS1_WHEAT^	-2.592	2.80E-12	-5.34514	4.12E-41
TRINITY_DN117628_c13_g317	RBS_AEGTA^R	-2.59452	4.88E-07	-4.37932	1.47E-13
TRINITY_DN117628_c13_g32	RBS2_WHEAT^	-2.60005	4.33E-07	-5.83963	1.97E-16
TRINITY_DN106795_c0_g1	SSL10_ARATH^	-2.60218	0.00062	-6.46346	4.87E-08
TRINITY_DN95645_c0_g1	PPD3_ARATH^	-2.60447	4.56E-08	-1.6186	0.000117
TRINITY_DN113021_c1_g2	MPK8_ORYSJ^	-2.61075	1.43E-05	-2.3601	1.95E-05
TRINITY_DN107131_c2_g1	LPA3_ARATH^I	-2.61473	4.51E-06	-3.73588	4.55E-11
TRINITY_DN109357_c3_g27	CP41B_ARATH	-2.61914	0.000116	-3.17401	3.21E-05
TRINITY_DN104664_c1_g8	RUBR_SYNY3^	-2.62257	0.000167	-3.24203	3.44E-06
TRINITY_DN19878_c0_g1	RBS1_WHEAT^	-2.62405	1.38E-12	-5.92949	1.93E-40
TRINITY_DN92972_c0_g2	PETM_SPIOL^F	-2.62608	1.96E-09	-4.87048	4.38E-27
TRINITY_DN78699_c0_g2	ACP3_HORVU^	-2.62626	1.09E-05	-3.14498	1.67E-07
TRINITY_DN106598_c1_g3	FAD3C_RICCO^	-2.62677	8.69E-08	-1.52094	0.000333
TRINITY_DN108503_c5_g14	F16P1_WHEAT	-2.62746	6.86E-05	-2.8656	9.3E-06
TRINITY_DN141217_c0_g1	#N/A	-2.62832	1.52E-05	-2.69246	2.39E-06
TRINITY_DN109075_c3_g1	F16P1_WHEAT	-2.63793	4.78E-07	-4.64997	1.47E-18
TRINITY_DN117628_c13_g224	RBS3_WHEAT^	-2.64201	0.000122	-6.91175	2.31E-11
TRINITY_DN99492_c0_g1	HOL3_ARATH^	-2.64529	6.44E-05	-1.82295	0.000552
TRINITY_DN90069_c0_g1	#N/A	-2.64636	4.22E-09	-2.7606	2.87E-15
TRINITY_DN101607_c1_g3	CRK26_ARATH	-2.64724	0.000338	-3.60709	1.04E-06
TRINITY_DN101443_c0_g3	#N/A	-2.6504	0.000898	-6.26372	2.98E-07

TRINITY_DN115940_c1_g1	VIP1_CAEEL^V	-2.65325	1.48E-06	-2.14368	3.22E-06
TRINITY_DN109308_c3_g1	CP41B_ARATH	-2.65672	2.50E-05	-3.46455	6.79E-07
TRINITY_DN108732_c3_g2	DTC_ARATH^D	-2.6662	1.41E-10	-4.70116	2.28E-23
TRINITY_DN109308_c3_g20	CP41B_ARATH	-2.6718	0.000194	-2.54717	0.000145
TRINITY_DN107075_c1_g32	GPPL1_ARATH	-2.67287	1.04E-10	-2.60407	1.03E-11
TRINITY_DN109357_c3_g10	CP41B_ARATH	-2.67684	0.000357	-4.17594	1.28E-06
TRINITY_DN109308_c3_g32	CP41B_ARATH	-2.67924	0.000158	-3.01757	2.5E-05
TRINITY_DN91863_c0_g1	OMT3_SORBI^	-2.68606	2.29E-05	-2.30206	1.21E-05
TRINITY_DN112517_c7_g34	G3PB_ARATH^	-2.69744	4.30E-07	-3.26092	1.5E-10
TRINITY_DN117628_c13_g229	RBS3_WHEAT^	-2.69996	2.87E-10	-3.72345	4.33E-18
TRINITY_DN47112_c0_g2	OMT2_SORBI^	-2.70285	0.000341	-3.68655	2.72E-15
TRINITY_DN110467_c13_g11	G3PB_PEA^G3	-2.70325	1.08E-06	-2.67926	1.01E-09
TRINITY_DN94201_c0_g3	SPA_SOLLC^SP	-2.70467	1.07E-06	-3.42921	3.8E-10
TRINITY_DN25405_c0_g1	#N/A	-2.70582	0.000178	-3.35529	1.21E-05
TRINITY_DN97468_c2_g1	CUT1B_ARATH	-2.7101	3.60E-11	-2.78926	5.8E-15
TRINITY_DN112517_c7_g48	G3PA_MAIZE^	-2.71061	6.27E-14	-3.26337	1.18E-21
TRINITY_DN107943_c0_g4	PSBW_ORYSJ^	-2.71271	0.000303	-4.2126	1.1E-06
TRINITY_DN70531_c0_g1	NU4C_AGRST^	-2.71339	7.35E-16	-1.6644	1.81E-10
TRINITY_DN94571_c0_g1	NU5C_AGRST^	-2.71505	5.34E-20	-2.88541	2.57E-24
TRINITY_DN95493_c0_g2	PPD3_ARATH^	-2.71529	5.76E-11	-2.42605	3.1E-13
TRINITY_DN112274_c0_g3	NAATA_HORVU	-2.71602	0.000277	-3.55664	1.05E-06
TRINITY_DN92227_c4_g2	PRSP1_SPIOL^	-2.72527	4.07E-06	-2.73501	3.73E-07
TRINITY_DN117628_c13_g113	RBS2_WHEAT^	-2.72543	0.000528	-6.60178	2E-08
TRINITY_DN110467_c13_g12	G3PB_PEA^G3	-2.73282	9.78E-09	-2.35933	2.98E-07
TRINITY_DN109308_c3_g14	CP41B_ARATH	-2.73492	8.39E-10	-2.31321	1.96E-09
TRINITY_DN117628_c13_g15	RBS1_WHEAT^	-2.73756	4.77E-05	-3.07418	1.44E-06
TRINITY_DN108503_c5_g1	F16P1_WHEAT	-2.75011	3.89E-14	-3.92523	1.53E-32
TRINITY_DN88467_c3_g14	#N/A	-2.75863	0.000139	-5.03422	2.09E-08
TRINITY_DN87312_c0_g1	PRPX_HORVU^	-2.75913	4.19E-08	-3.86554	1.21E-15
TRINITY_DN117628_c13_g4	RBS2_WHEAT^	-2.76822	1.03E-06	-4.72007	2.46E-15
TRINITY_DN117628_c13_g182	RBS_HORVU^R	-2.76829	4.07E-05	-5.78436	2E-14
TRINITY_DN108728_c4_g8	G3PB_PEA^G3	-2.77673	3.75E-09	-2.95574	1.8E-12
TRINITY_DN110811_c0_g1	COL1_ARATH^	-2.77762	7.86E-08	-1.85713	1.12E-06
TRINITY_DN109357_c3_g21	CP41B_ARATH	-2.7783	3.78E-09	-2.23285	2.42E-07
TRINITY_DN117231_c0_g1	PP186_ARATH	-2.7788	4.56E-07	-2.79786	2.38E-08
TRINITY_DN114672_c0_g3	ATPD_SORBI^A	-2.77958	1.05E-05	-2.0617	0.000206
TRINITY_DN111443_c2_g1	PSAG_HORVU^	-2.7798	5.28E-07	-3.65789	5.47E-13
TRINITY_DN101298_c1_g1	F16P2_SACHY^	-2.78166	1.67E-17	-3.50782	1.2E-30
TRINITY_DN107404_c2_g4	ALF_ARATH^A	-2.78168	5.24E-06	-2.3426	1.41E-05
TRINITY_DN117628_c13_g183	RBS2_ORYSJ^R	-2.78431	1.28E-06	-4.7724	2.09E-11
TRINITY_DN107925_c14_g3	RCAA_HORVU^	-2.78614	5.19E-11	-1.89527	4.49E-08
TRINITY_DN112637_c0_g6	E1311_ARATH^	-2.78938	1.78E-07	-4.58275	7.98E-16
TRINITY_DN98507_c6_g24	CAHC_HORVU^	-2.7913	1.54E-14	-4.91635	1.35E-35
TRINITY_DN108728_c4_g10	G3PA_SINAL^G	-2.79401	1.26E-06	-2.67788	2.52E-07
TRINITY_DN109357_c3_g11	CP41B_ARATH	-2.80818	2.39E-10	-2.89459	1.71E-12
TRINITY_DN109357_c3_g14	CP41B_ARATH	-2.8149	8.46E-13	-3.06466	6.31E-17
TRINITY_DN109982_c0_g2	DAAA_ARATH^	-2.81853	5.35E-06	-3.63869	1.22E-08

TRINITY_DN100514_c0_g10	#N/A	-2.81908	0.000939	-2.71047	0.000469
TRINITY_DN97671_c0_g2	TI110_PEA^TI1	-2.82414	2.32E-16	-4.75545	7E-46
TRINITY_DN108183_c8_g7	RCAA_HORVU^	-2.82563	1.70E-13	-2.30326	5.52E-12
TRINITY_DN96428_c0_g6	#N/A	-2.82635	1.03E-06	-4.5129	1.13E-11
TRINITY_DN107840_c0_g1	#N/A	-2.82855	0.000322	-3.39766	8.97E-06
TRINITY_DN81751_c0_g8	#N/A	-2.82984	5.18E-07	-4.68556	8.75E-12
TRINITY_DN110467_c13_g27	G3PA2_ARATH	-2.83635	1.22E-12	-3.11059	1.35E-13
TRINITY_DN95645_c0_g2	PPD3_ARATH^	-2.83741	9.73E-14	-2.59393	2E-14
TRINITY_DN107140_c0_g4	TRL31_ORYSJ^	-2.84173	1.89E-05	-3.5149	2.02E-07
TRINITY_DN114509_c1_g1	SCL6_ARATH^S	-2.84923	8.67E-05	-2.35188	0.000117
TRINITY_DN117628_c13_g126	RBS2_WHEAT^	-2.8494	5.29E-07	-4.98136	4.09E-15
TRINITY_DN94812_c1_g5	LIRP1_ORYSJ^L	-2.84965	7.86E-07	-4.35015	4.97E-18
TRINITY_DN110166_c1_g19	IPYR4_ARATH^	-2.85285	3.71E-09	-2.87382	6.33E-11
TRINITY_DN102980_c0_g1	NEP1_NEPGR^	-2.85382	0.00025	-4.67782	8.84E-07
TRINITY_DN117628_c13_g1	RBS3_WHEAT^	-2.85722	4.98E-05	-3.35211	2.39E-06
TRINITY_DN110351_c0_g4	Y3028_ARATH	-2.86125	3.75E-07	-2.38395	9.39E-07
TRINITY_DN109357_c3_g9	CP41B_ARATH	-2.86556	1.44E-12	-2.51031	6.29E-20
TRINITY_DN110925_c1_g1	NU4C_AGRST^	-2.86911	2.78E-11	-1.58454	1.25E-06
TRINITY_DN110863_c0_g4	COL1_ARATH^	-2.86919	2.14E-08	-1.79769	4E-05
TRINITY_DN107925_c14_g1	RCAA_HORVU^	-2.87114	4.05E-18	-2.26828	1.76E-09
TRINITY_DN47260_c0_g1	ZSS1_ZINZE^ZS	-2.87339	4.10E-09	-8.98749	5.26E-29
TRINITY_DN109357_c3_g20	CP41B_ARATH	-2.87381	7.80E-06	-3.40098	3.85E-08
TRINITY_DN109199_c4_g1	BGL03_ORYSJ^	-2.87383	8.36E-08	-4.12349	6.66E-12
TRINITY_DN112517_c7_g64	G3PA_MAIZE^	-2.87676	1.80E-16	-3.35424	9.98E-22
TRINITY_DN119037_c0_g4	ATOX1_ARATH	-2.88029	0.000433	-6.20665	6.82E-07
TRINITY_DN117628_c13_g112	RBS2_ORYSJ^R	-2.88137	5.54E-20	-4.44825	6.65E-45
TRINITY_DN201021_c0_g1	FQR1_ARATH^	-2.88218	0.000669	-6.2068	9.09E-07
TRINITY_DN88467_c3_g3	WIR1A_WHEAT	-2.88233	0.000696	-6.20626	8.28E-07
TRINITY_DN106252_c1_g2	U91C1_ARATH	-2.89308	3.28E-07	-5.01304	2.17E-13
TRINITY_DN118778_c0_g2	PIN4_ORYSJ^P	-2.90249	3.65E-08	-2.97482	2.07E-10
TRINITY_DN112517_c7_g26	G3PB_ARATH^	-2.90304	1.90E-08	-2.59758	2.14E-09
TRINITY_DN111675_c1_g1	AB11G_ARATH	-2.90324	0.000506	-6.50671	1.34E-07
TRINITY_DN110467_c13_g13	G3PA_MAIZE^	-2.90348	3.88E-16	-3.20013	3.71E-15
TRINITY_DN109308_c3_g18	CP41B_ARATH	-2.91003	4.66E-13	-2.68262	1.52E-14
TRINITY_DN111951_c3_g7	RBS1_WHEAT^	-2.91263	3.20E-14	-3.99728	1.28E-39
TRINITY_DN110274_c1_g1	#N/A	-2.91514	1.49E-06	-2.61196	1.05E-06
TRINITY_DN111929_c8_g16	KPPR_WHEAT^	-2.92013	5.46E-09	-3.14568	2.14E-09
TRINITY_DN92971_c0_g3	RBS3_WHEAT^	-2.92155	1.46E-11	-3.9748	1.22E-28
TRINITY_DN108809_c2_g1	PUM7_ARATH	-2.92845	4.22E-09	-3.159	1.04E-16
TRINITY_DN107884_c1_g3	ALF_ARATH^A	-2.92866	1.84E-14	-3.04015	1.24E-17
TRINITY_DN99616_c0_g3	#N/A	-2.93285	2.05E-05	-3.07383	1.23E-06
TRINITY_DN101219_c1_g1	CML29_ORYSJ^	-2.93939	8.01E-10	-4.57007	1.28E-17
TRINITY_DN111951_c3_g11	RBS_HORVU^F	-2.94018	8.03E-19	-4.52925	1.02E-40
TRINITY_DN106109_c0_g1	Y3720_ARATH	-2.94143	2.52E-07	-4.44564	1.43E-15
TRINITY_DN110467_c13_g14	G3PA_SINAL^C	-2.94334	9.48E-07	-3.74752	3.36E-11
TRINITY_DN111951_c3_g12	RBS_HORVU^F	-2.94911	1.44E-06	-3.2838	6.8E-08
TRINITY_DN102137_c0_g4	CCDA1_ORYSJ^	-2.94958	4.40E-16	-1.61874	1.62E-09

TRINITY_DN107404_c2_g1	ALF_ARATH^A	-2.95214	4.58E-21	-2.71561	5.69E-22
TRINITY_DN110467_c13_g18	G3PA_SINAL^G	-2.95563	9.79E-10	-3.47177	4.9E-14
TRINITY_DN105167_c1_g1	DLDH1_ARATH	-2.96037	1.46E-11	-3.28465	1.01E-15
TRINITY_DN109966_c2_g5	EXG_BLUGR^E	-2.96792	0.000948	-6.30301	1.71E-06
TRINITY_DN112517_c7_g70	G3PB_PEA^G3	-2.98003	2.32E-16	-3.19123	1.04E-22
TRINITY_DN114022_c2_g2	CRK25_ARATH	-2.98463	4.54E-05	-3.1017	7.52E-06
TRINITY_DN98677_c1_g2	#N/A	-2.98677	1.66E-07	-3.12886	4.69E-09
TRINITY_DN106598_c1_g6	FAD3C_RICCO	-2.99408	1.86E-07	-2.21315	4.77E-06
TRINITY_DN110666_c1_g2	DTC_ARATH^D	-2.99442	4.65E-15	-4.84943	1.28E-39
TRINITY_DN106203_c2_g2	KCR1_ARATH^	-2.9974	4.92E-06	-3.34492	1.42E-09
TRINITY_DN114367_c0_g10	LRK41_ARATH	-2.99842	6.79E-06	-2.16599	0.000176
TRINITY_DN99452_c4_g37	CAHC_HORVU	-3.00302	1.05E-10	-4.51043	1.11E-29
TRINITY_DN117628_c13_g223	RBS1_WHEAT^	-3.00437	0.000137	-3.37068	2.71E-06
TRINITY_DN117628_c13_g79	RBS2_WHEAT^	-3.01149	2.61E-10	-5.14739	3.64E-31
TRINITY_DN107884_c1_g13	ALF1_PEA^ALF	-3.0128	1.19E-17	-2.65819	2.65E-18
TRINITY_DN103314_c0_g1	CYP37_ARATH	-3.02273	6.10E-09	-2.39476	3.87E-07
TRINITY_DN111929_c8_g10	KPPR_WHEAT^	-3.02603	1.97E-06	-5.30607	5.47E-10
TRINITY_DN99452_c4_g29	CAHC_HORVU	-3.02617	1.24E-13	-5.58484	3.73E-36
TRINITY_DN117628_c13_g158	RBS3_WHEAT^	-3.02705	1.31E-07	-4.21688	1.19E-11
TRINITY_DN95088_c0_g1	PETM_SPIOL^P	-3.03047	3.61E-13	-4.57433	5.02E-35
TRINITY_DN85628_c0_g2	PRPX_HORVU^	-3.03327	7.37E-11	-4.34868	2.71E-22
TRINITY_DN111929_c8_g4	KPPR_WHEAT^	-3.04196	0.000285	-3.30073	1.26E-05
TRINITY_DN83776_c0_g1	NCS2_PAPSO^	-3.04767	1.59E-07	-3.48253	5.2E-10
TRINITY_DN117628_c13_g304	RBS2_WHEAT^	-3.04837	1.94E-16	-4.21687	6.03E-52
TRINITY_DN98066_c4_g5	PP11_ARATH^	-3.05346	1.35E-09	-4.69761	2.11E-24
TRINITY_DN109308_c3_g27	CP41B_ARATH	-3.05347	1.14E-08	-2.20983	2.81E-07
TRINITY_DN98507_c6_g20	CAHC_HORVU	-3.05851	1.38E-16	-3.21892	2.16E-26
TRINITY_DN102587_c0_g2	BEBT_CLABR^B	-3.06662	1.04E-07	-4.75893	1.64E-11
TRINITY_DN107036_c1_g1	P2C43_ORYSJ^	-3.07117	0.000505	-6.02502	4.49E-06
TRINITY_DN102706_c2_g2	AGAL_ORYSJ^A	-3.0722	9.95E-12	-3.2699	7.54E-15
TRINITY_DN112554_c1_g2	DHMA2_MYCT	-3.08344	5.03E-07	-2.55126	3.47E-06
TRINITY_DN108728_c4_g45	G3PB_ARATH^	-3.09251	1.43E-10	-2.27531	1.75E-09
TRINITY_DN66961_c0_g2	MT3_MUSAC^	-3.09479	5.37E-05	-2.98103	1.31E-05
TRINITY_DN64079_c2_g8	RBS1_WHEAT^	-3.097	6.44E-15	-4.0084	4.62E-32
TRINITY_DN108183_c8_g8	RCA_ORYSJ^RC	-3.10469	2.40E-22	-2.38808	3.68E-14
TRINITY_DN111929_c8_g25	KPPR_WHEAT^	-3.11051	9.48E-10	-2.99226	2.53E-12
TRINITY_DN108610_c12_g20	SGAT_ARATH^	-3.11071	3.89E-07	-2.1412	1.01E-05
TRINITY_DN100949_c1_g5	COL16_ARATH	-3.11085	8.00E-05	-3.00819	1.87E-05
TRINITY_DN99433_c1_g1	P2C05_ORYSJ^	-3.11338	8.43E-12	-1.30157	0.000684
TRINITY_DN117628_c13_g178	RBS_AEGTA^R	-3.11785	4.74E-06	-5.39861	2.44E-10
TRINITY_DN104664_c1_g12	RUBR_SYNY3^	-3.12058	9.51E-08	-2.5021	4.75E-07
TRINITY_DN117628_c13_g29	RBS3_WHEAT^	-3.12254	3.38E-14	-4.10831	7.14E-40
TRINITY_DN98507_c6_g15	CAHC_HORVU	-3.12442	1.98E-15	-2.71992	2.37E-17
TRINITY_DN119016_c6_g4	CBBY_RHOCA^	-3.132	7.40E-09	-1.5124	0.00042
TRINITY_DN117628_c13_g129	RBS2_ORYSJ^R	-3.13523	3.71E-24	-4.35281	1.87E-22
TRINITY_DN103671_c0_g5	NEP1_NEPGR^	-3.13823	1.11E-05	-7.40685	2.65E-11
TRINITY_DN117628_c13_g246	RBS3_WHEAT^	-3.14162	6.15E-08	-6.05965	1.04E-17

TRINITY_DN113956_c1_g5	CPNB4_ARATH	-3.14211	1.16E-09	-2.47447	6.33E-08
TRINITY_DN109308_c3_g13	CP41B_ARATH	-3.14264	2.83E-14	-3.02634	4.26E-17
TRINITY_DN107884_c1_g2	ALF_ARATH^A	-3.15058	5.32E-10	-2.83284	3.79E-09
TRINITY_DN110475_c1_g12	S17P_WHEAT^	-3.15929	2.50E-12	-2.95968	9.52E-14
TRINITY_DN2384_c0_g1	RBS1_WHEAT^	-3.16797	1.28E-09	-4.2849	7.41E-15
TRINITY_DN88983_c0_g4	RBS3_WHEAT^	-3.16997	5.63E-05	-4.46614	4.9E-08
TRINITY_DN111951_c3_g6	RBS_HORVU^F	-3.18749	1.03E-06	-7.22678	1.47E-13
TRINITY_DN98507_c6_g37	CAHC_HORVU^	-3.18783	5.48E-11	-5.55516	3.76E-27
TRINITY_DN786_c0_g1	#N/A	-3.19453	2.20E-07	-2.0031	4.02E-06
TRINITY_DN108240_c2_g1	PSBY_SPIOL^P	-3.2037	0.000975	-3.86965	0.000103
TRINITY_DN92433_c0_g1	PTR12_ARATH	-3.20411	0.000829	-3.86956	0.000791
TRINITY_DN117628_c13_g77	RBS1_WHEAT^	-3.23155	1.97E-12	-4.14424	1.12E-17
TRINITY_DN102688_c0_g2	#N/A	-3.24685	1.76E-08	-3.57047	7.04E-12
TRINITY_DN117628_c13_g324	RBS3_WHEAT^	-3.24857	1.28E-22	-4.53488	3.73E-47
TRINITY_DN111929_c8_g3	KPPR_WHEAT^	-3.25303	5.54E-09	-3.2859	8.24E-10
TRINITY_DN113578_c0_g4	PTR50_ARATH	-3.26278	1.65E-06	-1.83242	0.000815
TRINITY_DN111325_c1_g1	ATCA1_ARATH	-3.26617	4.74E-09	-4.96131	1.31E-14
TRINITY_DN117007_c0_g2	ABHD3_HUMA	-3.27234	5.86E-09	-2.71549	6.61E-09
TRINITY_DN99452_c4_g35	CAHC_HORVU^	-3.2826	1.94E-16	-4.28784	6.83E-30
TRINITY_DN100884_c1_g5	KCR1_ARATH^	-3.31714	5.85E-11	-3.60048	4.92E-14
TRINITY_DN117628_c13_g288	RBS1_WHEAT^	-3.31918	5.85E-05	-7.41658	3.66E-11
TRINITY_DN159723_c0_g1	#N/A	-3.32273	3.49E-14	-1.5725	4.94E-06
TRINITY_DN108728_c4_g34	G3PB_PEA^G3	-3.32481	3.44E-09	-3.59922	5.73E-11
TRINITY_DN119206_c1_g6	Y1684_ARATH	-3.34182	1.46E-06	-2.76761	1.66E-06
TRINITY_DN86856_c0_g7	TRY_ARATH^T	-3.34297	8.27E-07	-3.93288	7.86E-11
TRINITY_DN95343_c0_g1	RBS3_WHEAT^	-3.34702	1.13E-13	-4.4816	2.08E-23
TRINITY_DN112168_c0_g2	C70B2_ARATH	-3.34861	1.20E-07	-5.64311	1.93E-14
TRINITY_DN89785_c0_g1	#N/A	-3.36036	3.94E-07	-1.81371	0.000525
TRINITY_DN112047_c2_g8	ZFP1_ARATH^	-3.36173	3.06E-05	-3.25075	8.31E-06
TRINITY_DN90235_c0_g1	CUT1A_ARATH	-3.37215	9.18E-06	-4.12656	3.63E-08
TRINITY_DN105250_c0_g1	DLDH1_ARATH	-3.3883	1.66E-15	-2.93987	1.22E-16
TRINITY_DN108728_c4_g49	G3PA_SINAL^G	-3.39354	4.31E-06	-4.93634	5.65E-10
TRINITY_DN93460_c4_g2	CYNS_SORBI^C	-3.39578	0.000197	-3.30597	0.000104
TRINITY_DN114733_c1_g3	GBA2_HUMAN	-3.39777	3.32E-08	-1.62736	0.000863
TRINITY_DN104345_c2_g2	UBC5A_ORYSJ	-3.39861	2.36E-07	-2.20635	8.18E-06
TRINITY_DN99452_c4_g12	CAHC_HORVU^	-3.42402	2.41E-07	-2.50266	7.12E-06
TRINITY_DN105731_c0_g10	IPYR4_ARATH^	-3.42805	3.85E-10	-2.7148	2.03E-09
TRINITY_DN111585_c10_g48	THI42_VITVI^T	-3.4421	9.39E-10	-2.21122	6.22E-07
TRINITY_DN102506_c1_g1	GSXL5_ARATH	-3.45434	9.79E-08	-2.98915	7.66E-11
TRINITY_DN95343_c0_g2	RBS3_WHEAT^	-3.45767	1.43E-14	-3.86384	4.71E-20
TRINITY_DN88989_c0_g1	GSXL5_ARATH	-3.45894	1.20E-06	-3.22443	1.9E-07
TRINITY_DN122940_c0_g1	RBS3_WHEAT^	-3.46006	1.52E-05	-6.41812	2.8E-08
TRINITY_DN87474_c0_g3	LRK91_ARATH	-3.46218	8.84E-08	-4.24881	2.02E-09
TRINITY_DN88718_c1_g2	#N/A	-3.48219	2.28E-05	-5.55042	5.16E-08
TRINITY_DN116835_c1_g22	LINS_ORYSJ^LI	-3.48977	0.000624	-4.15299	0.000358
TRINITY_DN110467_c13_g9	G3PA_SINAL^G	-3.49052	1.31E-07	-2.00391	0.000337
TRINITY_DN98507_c6_g26	CAHC_HORVU^	-3.49973	5.27E-18	-4.34312	2.77E-31

TRINITY_DN102902_c0_g5	CCDA1_ORYSJ^	-3.50687	2.19E-06	-2.41906	7.06E-05
TRINITY_DN112517_c7_g39	G3PB_PEA^G3	-3.50769	4.87E-09	-3.84407	7.49E-11
TRINITY_DN108426_c6_g2	SGAT_ARATH^	-3.5097	2.15E-07	-2.19158	5.83E-05
TRINITY_DN29857_c1_g1	RBS1_WHEAT^	-3.51259	4.65E-06	-6.83914	1.86E-09
TRINITY_DN117628_c13_g105	RBS2_WHEAT^	-3.51481	0.000573	-4.69582	7.67E-06
TRINITY_DN117628_c13_g245	RBS_AEGTA^R	-3.51522	1.44E-18	-5.41004	6.73E-40
TRINITY_DN112517_c7_g31	G3PB_PEA^G3	-3.53014	1.38E-09	-3.60571	3.47E-10
TRINITY_DN116395_c0_g2	AB11G_ARATH	-3.53275	2.85E-08	-3.02785	1.07E-07
TRINITY_DN117628_c13_g196	RBS3_WHEAT^	-3.53422	0.000455	-5.97072	2.07E-05
TRINITY_DN116835_c1_g17	LINS_ORYSJ^LI	-3.5581	4.22E-06	-2.14386	0.000577
TRINITY_DN110467_c13_g3	G3PA_MAIZE^	-3.56528	6.85E-06	-2.32746	0.000296
TRINITY_DN107884_c1_g8	ALF1_PEA^ALF	-3.56877	1.26E-11	-3.42389	7.14E-12
TRINITY_DN113887_c0_g1	C3H54_ORYSJ^	-3.5732	9.65E-06	-4.32277	4.87E-07
TRINITY_DN108183_c8_g1	RCAA_HORVU^	-3.5941	4.40E-15	-1.77809	5.49E-08
TRINITY_DN107404_c2_g5	ALF1_PEA^ALF	-3.62454	1.06E-08	-3.72891	4.03E-10
TRINITY_DN94449_c2_g3	#N/A	-3.62622	5.19E-11	-2.45479	2.68E-08
TRINITY_DN104142_c0_g3	APG2_ARATH^	-3.63294	4.30E-05	-4.2983	2.39E-06
TRINITY_DN117628_c13_g145	RBS1_WHEAT^	-3.64976	3.83E-05	-4.81855	3.25E-06
TRINITY_DN117628_c13_g263	RBS2_WHEAT^	-3.65066	3.94E-06	-4.81475	3.6E-09
TRINITY_DN112844_c10_g41	G3PB_ARATH^	-3.68079	0.000378	-3.07794	0.000145
TRINITY_DN117628_c13_g312	RBS3_WHEAT^	-3.68605	4.23E-08	-4.04422	8.62E-10
TRINITY_DN117628_c13_g212	RBS2_WHEAT^	-3.70178	1.94E-07	-5.53467	1.47E-10
TRINITY_DN115908_c1_g13	CCSA_AGRST^	-3.71446	8.08E-05	-3.09155	0.000111
TRINITY_DN108183_c8_g10	RCAA_HORVU^	-3.73043	3.25E-16	-2.28774	2.77E-08
TRINITY_DN98507_c6_g9	CAHC_HORVU^	-3.73544	0.000793	-6.17553	5.72E-05
TRINITY_DN104345_c2_g1	UBC5A_ORYSJ^	-3.75441	1.45E-08	-2.14674	8.14E-06
TRINITY_DN27492_c0_g2	#N/A	-3.75495	2.16E-05	-3.14107	2.56E-05
TRINITY_DN113511_c1_g1	PER2_MAIZE^I	-3.76404	1.02E-05	-4.1662	6.05E-07
TRINITY_DN99393_c2_g1	PER54_ARATH	-3.76463	3.78E-09	-5.15781	1.07E-39
TRINITY_DN115213_c3_g1	#N/A	-3.7654	0.000404	-3.66329	0.000883
TRINITY_DN103470_c9_g15	GLO1_ORYSJ^	-3.79492	4.59E-09	-2.38503	1.31E-06
TRINITY_DN98507_c6_g10	CAHC_HORVU^	-3.8055	3.54E-22	-5.34805	8.26E-41
TRINITY_DN105941_c0_g8	DR100_ARATH	-3.82387	2.87E-06	-3.42285	4.98E-06
TRINITY_DN29857_c0_g1	RBS1_WHEAT^	-3.85071	3.07E-12	-4.8529	9.04E-17
TRINITY_DN79042_c0_g1	#N/A	-3.86206	1.27E-10	-5.6898	2.42E-15
TRINITY_DN117628_c13_g248	RBS2_WHEAT^	-3.89553	6.45E-18	-4.72613	1.6E-27
TRINITY_DN99452_c4_g25	CAHC_HORVU^	-3.8978	2.27E-16	-5.62947	4.6E-27
TRINITY_DN95928_c0_g2	NAC22_ARATH	-3.91256	9.30E-06	-4.57551	2.6E-06
TRINITY_DN99452_c4_g14	CAHC_HORVU^	-3.9272	1.01E-15	-7.05307	3.72E-31
TRINITY_DN105439_c0_g1	LSD1_ORYSJ^L	-3.93131	1.44E-06	-3.81966	3.76E-07
TRINITY_DN161616_c0_g1	#N/A	-3.94671	7.44E-21	-1.83435	6.01E-10
TRINITY_DN108728_c4_g18	G3PB_ARATH^	-3.95686	2.66E-08	-3.47551	1.85E-08
TRINITY_DN117628_c13_g315	RBS_AEGTA^R	-3.95995	1.19E-33	-5.07853	1.87E-60
TRINITY_DN110467_c13_g19	G3PB_PEA^G3	-3.96002	7.23E-11	-3.8456	2.62E-12
TRINITY_DN111950_c0_g3	ASPG2_ARATH	-3.96129	5.42E-06	-3.85234	1.31E-06
TRINITY_DN27632_c0_g1	#N/A	-3.97927	8.89E-15	-2.4216	1.21E-09
TRINITY_DN110467_c13_g32	G3PB_SPIOL^	-4.00087	2.32E-05	-3.00215	0.000163

TRINITY_DN108183_c8_g9	RCAA_HORVU^	-4.01418	1.13E-19	-2.43363	6.11E-09
TRINITY_DN106982_c2_g2	WAK2_ARATH^	-4.01577	1.91E-06	-4.6777	2.1E-08
TRINITY_DN98542_c3_g1	PER54_ARATH	-4.01618	2.33E-19	-5.62192	6.49E-60
TRINITY_DN117628_c13_g297	RBS1_WHEAT^	-4.01961	1.83E-11	-4.18953	1.05E-11
TRINITY_DN82761_c0_g3	#N/A	-4.04996	0.00017	-3.94507	0.000732
TRINITY_DN117628_c13_g110	RBS2_WHEAT^	-4.06928	3.65E-13	-5.14333	2.21E-22
TRINITY_DN14123_c0_g1	PPD3_ARATH^	-4.08215	1.61E-10	-1.68176	0.000424
TRINITY_DN82335_c0_g2	RBS2_ORYSJ^R	-4.12429	7.71E-05	-4.78673	3.46E-06
TRINITY_DN105879_c1_g7	E13C_HORVU^	-4.18572	1.56E-07	-6.64505	1.13E-09
TRINITY_DN89986_c0_g1	#N/A	-4.20241	0.000174	-3.32525	0.000144
TRINITY_DN112844_c10_g30	G3PB_ARATH^	-4.22745	0.000148	-4.10803	0.000309
TRINITY_DN105142_c0_g1	KCS12_ARATH^	-4.26973	3.06E-05	-4.16843	1.18E-05
TRINITY_DN106744_c2_g2	BGL12_ORYSI^	-4.30169	5.48E-11	-5.06612	2.65E-13
TRINITY_DN117628_c13_g339	RBS2_ORYSJ^R	-4.30716	2.17E-13	-4.19875	2.57E-15
TRINITY_DN100180_c0_g2	HD1_ORYSJ^H	-4.32764	6.44E-11	-7.28044	6.95E-15
TRINITY_DN94812_c1_g6	PP11_ARATH^	-4.33147	2.90E-14	-5.87754	2.38E-22
TRINITY_DN110067_c0_g1	NEP1_NEPGR^	-4.35033	7.87E-09	-4.23949	1.34E-08
TRINITY_DN156195_c0_g1	RBS3_WHEAT^	-4.35057	7.84E-09	-6.80409	8.45E-11
TRINITY_DN107143_c0_g1	CHAT_ARATH^	-4.3845	7.40E-16	-5.68607	5.35E-22
TRINITY_DN109357_c3_g19	CP41B_ARATH	-4.38666	0.000202	-4.29355	9.94E-06
TRINITY_DN99452_c4_g15	CAHC_HORVU^	-4.40987	4.62E-06	-6.86594	1.3E-08
TRINITY_DN105368_c10_g10	12KD_FRAAN^	-4.43985	2.17E-15	-6.49554	2.07E-23
TRINITY_DN106744_c2_g5	BGL12_ORYSI^	-4.45362	1.13E-07	-6.91456	1.07E-09
TRINITY_DN94251_c0_g1	JI23_HORVU^J	-4.45942	0.00013	-6.14863	5.73E-06
TRINITY_DN98066_c4_g4	LIRP1_ORYSJ^L	-4.47335	5.70E-16	-5.07644	3.11E-20
TRINITY_DN136692_c0_g1	#N/A	-4.48571	1.93E-05	-3.58816	0.000247
TRINITY_DN104832_c1_g1	LIP1_PSYIM^LI	-4.51452	1.99E-10	-4.41444	1.33E-11
TRINITY_DN99452_c4_g8	CAHC_HORVU^	-4.51894	2.71E-08	-4.41153	5.61E-09
TRINITY_DN107884_c1_g6	ALF1_PEA^ALF	-4.53491	9.55E-16	-2.72285	8.71E-09
TRINITY_DN86325_c0_g2	CUT1A_ARATH	-4.56321	9.01E-06	-6.2489	3.32E-06
TRINITY_DN92840_c1_g5	XTH22_ARATH	-4.56948	6.12E-05	-3.19782	9.54E-05
TRINITY_DN117628_c13_g156	RBS3_WHEAT^	-4.58001	8.15E-09	-3.61667	9.67E-08
TRINITY_DN100865_c0_g1	GDL29_ARATH	-4.71205	1.17E-10	-7.67618	7.41E-14
TRINITY_DN117628_c13_g201	RBS2_WHEAT^	-4.71477	8.18E-11	-7.16857	1.27E-13
TRINITY_DN98601_c0_g1	E1313_ARATH^	-4.75071	1.52E-13	-4.95174	1.11E-16
TRINITY_DN117628_c13_g291	RBS2_WHEAT^	-4.78663	3.84E-10	-3.80285	2.02E-09
TRINITY_DN117628_c13_g132	RBS2_WHEAT^	-4.80938	2.78E-13	-4.03982	4.02E-13
TRINITY_DN114286_c5_g12	RADL3_ARATH	-4.81282	1.97E-11	-5.2004	1.88E-12
TRINITY_DN97835_c0_g2	GDL34_ARATH	-4.82421	9.06E-07	-6.51322	7.69E-09
TRINITY_DN96923_c1_g3	PER1_SORBI^P	-4.9094	6.36E-07	-4.03243	9.34E-07
TRINITY_DN110651_c0_g7	KCS12_ARATH^	-4.93038	5.68E-10	-3.95061	2.91E-09
TRINITY_DN115601_c5_g5	LIP1_PSYIM^LI	-4.95115	1.07E-08	-7.4206	1.75E-10
TRINITY_DN116835_c1_g15	LINS_ORYSJ^LI	-4.98358	8.71E-11	-2.75806	5.94E-07
TRINITY_DN98713_c0_g1	GDL29_ARATH	-4.99175	9.31E-08	-7.4621	1.65E-10
TRINITY_DN109349_c0_g4	ACMAT_VITLA^	-5.0851	7.38E-17	-5.62576	7.81E-19
TRINITY_DN112068_c1_g4	PPCE_BOVIN^P	-5.09726	7.44E-21	-5.88952	4E-27
TRINITY_DN117628_c13_g290	RBS_AEGTA^R	-5.22662	4.32E-10	-6.91334	3.79E-11

TRINITY_DN69663_c0_g3	RAF1A_WHEA⁻	-5.28895	3.40E-15	-5.17405	2.14E-17
TRINITY_DN117628_c13_g52	RBS_AEGTA^R	-5.31469	4.69E-22	-5.86078	2.85E-27
TRINITY_DN82335_c0_g11	RBS2_ORYSJ^R	-5.42261	2.13E-13	-4.14314	1.62E-12
TRINITY_DN97901_c0_g2	REXO4_XENLA	-5.6306	0.000737	-5.56944	0.00029
TRINITY_DN107429_c1_g6	DR100_ARATH	-5.64363	1.51E-07	-3.191	1.28E-05
TRINITY_DN77124_c0_g4	#N/A	-5.65242	0.000667	-5.58124	0.000369
TRINITY_DN113937_c4_g2	PER52_ARATH	-5.86516	0.000531	-5.80351	0.000313
TRINITY_DN75456_c0_g1	#N/A	-5.88061	1.23E-11	-7.56586	1.3E-12
TRINITY_DN107872_c3_g1	E1313_ARATH	-6.01064	1.33E-10	-4.60421	1.04E-08
TRINITY_DN59216_c0_g1	#N/A	-6.0114	3.38E-13	-7.69707	2.67E-14
TRINITY_DN117628_c13_g293	RBS2_WHEAT^	-6.08629	4.53E-05	-6.02243	1.95E-05
TRINITY_DN113937_c4_g1	PER52_ARATH	-6.13719	5.53E-18	-6.81409	4.38E-22
TRINITY_DN112068_c1_g3	#N/A	-6.2152	8.11E-05	-6.15039	3.6E-05
TRINITY_DN112904_c1_g1	PPCE_HUMAN	-6.24296	4.35E-23	-5.44756	3.49E-24
TRINITY_DN99452_c4_g31	CAHC_HORVU	-6.26947	8.32E-06	-6.20522	2.68E-06
TRINITY_DN117628_c13_g153	RBS2_WHEAT^	-6.29751	0.000107	-6.23729	4.63E-05
TRINITY_DN117628_c13_g206	RBS2_WHEAT^	-6.30937	3.83E-05	-6.24786	1.48E-05
TRINITY_DN113511_c1_g2	PER14_ARATH	-6.43003	9.21E-07	-6.3668	2.01E-07
TRINITY_DN109966_c2_g2	EXG_BLUGR^E	-6.63026	1.06E-06	-2.83035	0.000162
TRINITY_DN20589_c0_g2	OMT2_SORBI^	-6.64246	1.41E-06	-6.57115	4.4E-07
TRINITY_DN100813_c0_g3	PER4_VITVI^PE	-6.93369	4.72E-25	-8.4582	3.32E-30
TRINITY_DN113964_c0_g3	NATT3_THANI	-6.9361	3.32E-09	-2.1913	0.000311
TRINITY_DN117628_c13_g14	RBS1_WHEAT^	-7.11999	8.32E-10	-5.26575	6.81E-11
TRINITY_DN82249_c0_g1	#N/A	-7.53472	4.39E-12	-7.47176	2.89E-13
TRINITY_DN84265_c0_g1	#N/A	-7.58342	4.71E-11	-7.51983	3.76E-12
TRINITY_DN108629_c1_g9	BGL03_ORYSJ^	-7.87048	2.36E-10	-7.80943	3.69E-11
TRINITY_DN107429_c1_g5	PSKR2_ARATH	-8.077	1.41E-17	-1.99666	3.18E-06
TRINITY_DN40642_c0_g2	RAF1A_WHEA⁻	-8.12153	2.42E-17	-4.3241	1.79E-15
TRINITY_DN113597_c1_g1	BGL03_ORYSJ^	-8.21637	2.17E-11	-8.15384	2.71E-12
TRINITY_DN101060_c1_g6	PER4_VITVI^PE	-8.3006	1.05E-15	-8.23725	1.51E-17
TRINITY_DN101060_c1_g1	PER4_VITVI^PE	-9.13178	3.87E-20	-9.06884	1.28E-22

ress in Agrostis stolonifera
