# Supplementary material for: Comparative transcriptomic analysis reveals common molecular factors responsive to heat and drought stress in Agrostis stolonifera
Source: Sci Rep. 2018 Oct 12;8:15181. doi: 10.1038/s41598-018-33597-3 (PMC6185948; doi:10.1038/s41598-018-33597-3)
Supplement: Supplementary file 2 — supplemental table 2 [file 41598_2018_33597_MOESM2_ESM.pdf]

Comparative transcriptomic analysis reveals common molecular factors responsive to heat and drought

Yi Xu and Bingru Huang

GOID	Ontology	Term	Level	q	m	t	k	probes
GO:00164	molecular_	oxidoreduc	1	45	4899	51576	208	TRINITY_DI
GO:00442	biological_	cellular bio	3	69	11353	51576	208	TRINITY_DI
GO:19015	biological_	organic sub	2	68	11529	51576	208	TRINITY_DI
GO:00096	biological_	response to	1	31	3985	51576	208	TRINITY_DI
GO:00047	molecular_	phosphopr	1	10	525	51576	208	TRINITY_DI
GO:00047	molecular_	protein ser	1	10	268	51576	208	TRINITY_DI
GO:00096	biological_	response to	1	10	541	51576	208	TRINITY_DI
GO:00060	biological_	organic aci	5	32	3516	51576	208	TRINITY_DI
GO:00197	biological_	carboxylic a	5	27	3114	51576	208	TRINITY_DI
GO:00434	biological_	oxoacid me	5	30	3227	51576	208	TRINITY_DI
GO:00010	molecular_	nucleic acid	1	21	2011	51576	208	TRINITY_DI
GO:00037	molecular_	transcriptio	1	21	2005	51576	208	TRINITY_DI
GO:00098	biological_	ethylene-a	7	8	369	51576	208	TRINITY_DI
GO:00713	biological_	cellular res	5	8	383	51576	208	TRINITY_DI
GO:00102	biological_	response to	4	7	201	51576	208	TRINITY_DI
GO:00102	biological_	response to	3	9	344	51576	208	TRINITY_DI
GO:19016	biological_	response to	1	10	634	51576	208	TRINITY_DI
GO:00167	molecular_	oxidoreduc	1	10	177	51576	208	TRINITY_DI
GO:00167	molecular_	oxidoreduc	2	10	145	51576	208	TRINITY_DI
GO:00512	molecular_	dioxygenas	1	11	356	51576	208	TRINITY_DI
GO:00160	biological_	organic aci	6	21	1621	51576	208	TRINITY_DI
GO:00442	biological_	small mole	3	21	1954	51576	208	TRINITY_DI
GO:00463	biological_	carboxylic a	7	19	1393	51576	208	TRINITY_DI
GO:00314	biological_	oxylipin me	5	9	183	51576	208	TRINITY_DI
GO:00314	biological_	oxylipin bio	8	9	181	51576	208	TRINITY_DI
GO:00435	molecular_	sequence-s	2	12	899	51576	208	TRINITY_DI
GO:00065	biological_	proline me	6	5	51	51576	208	TRINITY_DI
GO:00065	biological_	proline bio	10	4	44	51576	208	TRINITY_DI
GO:00166	molecular_	oxidoreduc	1	6	105	51576	208	TRINITY_DI
GO:00166	molecular_	oxidoreduc	1	3	26	51576	208	TRINITY_DI
GO:00047	molecular_	saccharopi	1	2	7	51576	208	TRINITY_DI
GO:00471	molecular_	saccharopi	1	2	7	51576	208	TRINITY_DI
GO:00471	molecular_	saccharopi	1	2	7	51576	208	TRINITY_DI
GO:00400	biological_	regulation	2	4	80	51576	208	TRINITY_DI
GO:00485	biological_	regulation	3	4	68	51576	208	TRINITY_DI
GO:00485	biological_	regulation	3	4	68	51576	208	TRINITY_DI
GO:00043	molecular_	glutamate	2	3	17	51576	208	TRINITY_DI
GO:00043	molecular_	glutamate-	1	3	17	51576	208	TRINITY_DI
GO:00166	molecular_	oxidoreduc	1	7	298	51576	208	TRINITY_DI
GO:00169	molecular_	oxidoreduc	1	8	426	51576	208	TRINITY_DI
GO:00551	biological_	L-proline bi	10	3	32	51576	208	TRINITY_DI

: stress in Agrostis stolonifera

**annotation**	**log_odds_r**	**p**
// // // /,	1.187554	0.000255
// // // /,	0.591711	0.05446
// // // /,	0.548456	0.092916
// // // /,	0.947805	0.085044
// // // /,	2.239727	0.031003
// // // /,	3.209811	0.000279
// // // /,	2.196416	0.034523
// // // /,	1.174253	0.010583
// // // /,	1.104307	0.056854
// // // /,	1.204885	0.012692
// // // /,	1.372593	0.033246
// // // /,	1.376903	0.033246
// // // /,	2.426496	0.05446
// // // /,	2.372772	0.058821
// // // /,	3.110276	0.012958
// // // /,	2.697633	0.010484
// // // /,	1.967562	0.085044
// // // /,	3.808295	1.66E-05
// // // /,	4.095992	4.77E-06
// // // /,	2.937671	0.000379
// // // /,	1.683622	0.003383
// // // /,	1.414075	0.025651
// // // /,	1.757921	0.00426
// // // /,	3.608198	0.00017
// // // /,	3.624052	0.00017
// // // /,	1.726758	0.085044
// // // /,	4.603475	0.002603
// // //	4.494541	0.019486
// // // /,	3.82469	0.00426
// //	4.838495	0.056854
//	6.146618	0.085044
//	6.146618	0.085044
//	6.146618	0.085044
// // //	3.632044	0.085044
// // //	3.86651	0.057807
// // //	3.86651	0.057807
// //	5.451472	0.023758
// //	5.451472	0.023758
// // // /,	2.542159	0.07132
// // // /,	2.219263	0.090649
// //	4.538935	0.085044
