# Supplementary material for: Comparative transcriptomic analysis reveals common molecular factors responsive to heat and drought stress in Agrostis stolonifera
Source: Sci Rep. 2018 Oct 12;8:15181. doi: 10.1038/s41598-018-33597-3 (PMC6185948; doi:10.1038/s41598-018-33597-3)
Supplement: Supplementary file 3 — supplemental table 3 [file 41598_2018_33597_MOESM3_ESM.pdf]

Comparative transcriptomic analysis reveals common molecular factors responsive to heat and drought
Yi Xu and Bingru Huang

GOID	Ontology	Term	Level	q	m	t	k	probes
GO:001597	biological_	photosynth	2	75	733	51576	309	TRINITY_DI
GO:001925	biological_	reductive p	5	34	137	51576	309	TRINITY_DI
GO:001968	biological_	photosynth	4	34	137	51576	309	TRINITY_DI
GO:001597	biological_	carbon fixa	2	34	172	51576	309	TRINITY_DI
GO:000985	biological_	photorespi	3	31	235	51576	309	TRINITY_DI
GO:004309	biological_	cellular me	3	31	371	51576	309	TRINITY_DI
GO:001605	biological_	carbohydra	4	40	1068	51576	309	TRINITY_DI
GO:000597	biological_	carbohydra	2	67	3503	51576	309	TRINITY_DI
GO:004472	biological_	single-orga	3	51	2188	51576	309	TRINITY_DI
GO:004471	biological_	single-orga	3	72	5174	51576	309	TRINITY_DI
GO:000609	biological_	generation	2	28	991	51576	309	TRINITY_DI
GO:001968	biological_	photosynth	3	17	333	51576	309	TRINITY_DI
GO:004471	biological_	single-orga	2	108	9903	51576	309	TRINITY_DI
GO:000815	biological_	metabolic p	1	206	25173	51576	309	TRINITY_DI
GO:000762	biological_	circadian rh	1	14	263	51576	309	TRINITY_DI
GO:004851	biological_	rhythmic p	1	15	317	51576	309	TRINITY_DI
GO:004423	biological_	cellular me	2	181	22184	51576	309	TRINITY_DI
GO:000599	biological_	monosacch	3	14	370	51576	309	TRINITY_DI
GO:003254	biological_	plastid trar	9	5	21	51576	309	TRINITY_DI
GO:000976	biological_	photosynth	4	8	98	51576	309	TRINITY_DI
GO:002290	biological_	electron tra	4	10	198	51576	309	TRINITY_DI
GO:004428	biological_	small mole	2	59	5176	51576	309	TRINITY_DI
GO:000906	biological_	serine fami	5	10	224	51576	309	TRINITY_DI
GO:001010	biological_	regulation	3	7	102	51576	309	TRINITY_DI
GO:000654	biological_	glycine met	6	7	103	51576	309	TRINITY_DI
GO:001597	biological_	carbon utili	1	4	20	51576	309	TRINITY_DI
GO:000922	biological_	thiamine bi	9	5	43	51576	309	TRINITY_DI
GO:004272	biological_	thiamine-c	9	5	43	51576	309	TRINITY_DI
GO:001931	biological_	hexose me	3	10	286	51576	309	TRINITY_DI
GO:000677	biological_	thiamine m	7	5	48	51576	309	TRINITY_DI
GO:004272	biological_	thiamine-c	7	5	48	51576	309	TRINITY_DI
GO:000600	biological_	glucose me	3	8	176	51576	309	TRINITY_DI
GO:000977	biological_	photosynth	4	5	55	51576	309	TRINITY_DI
GO:000985	biological_	oxidative p	4	6	94	51576	309	TRINITY_DI
GO:005511	biological_	oxidation-r	2	20	1164	51576	309	TRINITY_DI
GO:003425	biological_	positive reg	7	5	61	51576	309	TRINITY_DI
GO:004572	biological_	positive reg	12	5	61	51576	309	TRINITY_DI
GO:000998	biological_	cellular pro	1	219	30883	51576	309	TRINITY_DI
GO:000905	biological_	biosyntheti	1	105	12404	51576	309	TRINITY_DI
GO:000598	biological_	starch meta	5	8	234	51576	309	TRINITY_DI
GO:190156	biological_	organonitro	2	48	4604	51576	309	TRINITY_DI
GO:001968	biological_	glyceraldeh	6	7	216	51576	309	TRINITY_DI
GO:000815	biological_	biological_	0	264	40045	51576	309	TRINITY_DI

GO:007252	biological_	pyridine-co	5	10	470	51576	309	TRINITY_DI
GO:000608	biological_	cellular ald	4	8	316	51576	309	TRINITY_DI
GO:001829	biological_	protein-chr	3	6	186	51576	309	TRINITY_DI
GO:000907	biological_	serine fami	9	5	128	51576	309	TRINITY_DI
GO:190160	biological_	alpha-amin	5	13	791	51576	309	TRINITY_DI
GO:000953	cellular_co	plastid	5	164	6248	51576	309	TRINITY_DI
GO:000950	cellular_co	chloroplast	5	162	6146	51576	309	TRINITY_DI
GO:004443	cellular_co	chloroplast	6	113	3299	51576	309	TRINITY_DI
GO:004443	cellular_co	plastid part	6	113	3311	51576	309	TRINITY_DI
GO:000953	cellular_co	chloroplast	8	71	1187	51576	309	TRINITY_DI
GO:003197	cellular_co	plastid thyl	7	71	1187	51576	309	TRINITY_DI
GO:000957	cellular_co	thylakoid	3	73	1293	51576	309	TRINITY_DI
GO:004443	cellular_co	thylakoid p	4	66	1104	51576	309	TRINITY_DI
GO:004265	cellular_co	thylakoid m	6	63	1004	51576	309	TRINITY_DI
GO:003435	cellular_co	photosynth	6	63	1006	51576	309	TRINITY_DI
GO:000953	cellular_co	chloroplast	10	62	976	51576	309	TRINITY_DI
GO:005503	cellular_co	plastid thyl	8	62	976	51576	309	TRINITY_DI
GO:003198	cellular_co	organelle s	5	71	1980	51576	309	TRINITY_DI
GO:004444	cellular_co	cytoplasmi	4	207	18759	51576	309	TRINITY_DI
GO:000953	cellular_co	plastid stro	6	54	1486	51576	309	TRINITY_DI
GO:000957	cellular_co	chloroplast	7	53	1470	51576	309	TRINITY_DI
GO:000573	cellular_co	cytoplasm	3	214	22666	51576	309	TRINITY_DI
GO:000952	cellular_co	photosyste	8	21	245	51576	309	TRINITY_DI
GO:000952	cellular_co	plastid env	7	45	1622	51576	309	TRINITY_DI
GO:000994	cellular_co	chloroplast	8	43	1587	51576	309	TRINITY_DI
GO:001031	cellular_co	stromule	8	12	66	51576	309	TRINITY_DI
GO:004322	cellular_co	membrane	1	211	25223	51576	309	TRINITY_DI
GO:004323	cellular_co	intracellula	4	211	25219	51576	309	TRINITY_DI
GO:004444	cellular_co	intracellula	4	125	12264	51576	309	TRINITY_DI
GO:004322	cellular_co	organelle	1	214	26430	51576	309	TRINITY_DI
GO:004322	cellular_co	intracellula	4	214	26425	51576	309	TRINITY_DI
GO:004442	cellular_co	organelle p	2	125	12350	51576	309	TRINITY_DI
GO:003196	cellular_co	organelle e	6	46	2640	51576	309	TRINITY_DI
GO:003197	cellular_co	envelope	2	46	2641	51576	309	TRINITY_DI
GO:000952	cellular_co	photosyste	8	13	197	51576	309	TRINITY_DI
GO:000952	cellular_co	photosyste	8	11	136	51576	309	TRINITY_DI
GO:004442	cellular_co	intracellula	3	227	30331	51576	309	TRINITY_DI
GO:000562	cellular_co	intracellula	2	229	30779	51576	309	TRINITY_DI
GO:009879	cellular_co	membrane	4	26	1275	51576	309	TRINITY_DI
GO:000557	cellular_co	extracellula	1	39	2600	51576	309	TRINITY_DI
GO:004804	cellular_co	apoplast	1	20	876	51576	309	TRINITY_DI
GO:000954	cellular_co	chloroplast	10	9	155	51576	309	TRINITY_DI
GO:003197	cellular_co	plastid thyl	9	9	155	51576	309	TRINITY_DI
GO:000965	cellular_co	photosyste	10	6	68	51576	309	TRINITY_DI
GO:000953	cellular_co	photosyste	10	4	18	51576	309	TRINITY_DI
GO:003197	cellular_co	thylakoid lu	4	9	200	51576	309	TRINITY_DI
GO:000577	cellular_co	peroxisome	5	15	630	51576	309	TRINITY_DI

GO:004257	cellular_co	microbody	5	15	630	51576	309	TRINITY_DI
GO:199020	cellular_co	oxidoreduc	4	9	226	51576	309	TRINITY_DI
GO:004446	cellular_co	cell part	2	255	37379	51576	309	TRINITY_DI
GO:000562	cellular_co	cell	1	255	37520	51576	309	TRINITY_DI
GO:001028	cellular_co	plastoglobu	8	8	204	51576	309	TRINITY_DI
GO:001698	molecular_	ribulose-bis	1	16	40	51576	309	TRINITY_DI
GO:001649	molecular_	oxidoreduc	1	70	4899	51576	309	TRINITY_DI
GO:001682	molecular_	lyase activi	1	31	1181	51576	309	TRINITY_DI
GO:001683	molecular_	carboxy-lya	1	16	314	51576	309	TRINITY_DI
GO:001683	molecular_	carbon-carl	1	17	444	51576	309	TRINITY_DI
GO:004803	molecular_	cofactor bi	1	32	1595	51576	309	TRINITY_DI
GO:000408	molecular_	carbonate (	1	6	33	51576	309	TRINITY_DI
GO:004710	molecular_	glyceraldeh	1	5	26	51576	309	TRINITY_DI
GO:000845	molecular_	alanine-gly	1	5	29	51576	309	TRINITY_DI
GO:001920	molecular_	carbohydra	1	8	124	51576	309	TRINITY_DI
GO:000449	molecular_	monooxyge	1	21	1024	51576	309	TRINITY_DI
GO:000848	molecular_	transamina	1	8	152	51576	309	TRINITY_DI
GO:001676	molecular_	transferase	1	8	152	51576	309	TRINITY_DI
GO:001029	molecular_	heteropoly	2	4	19	51576	309	TRINITY_DI
GO:005030	molecular_	sugar-phos	1	6	71	51576	309	TRINITY_DI
GO:000432	molecular_	ferredoxin-	1	4	23	51576	309	TRINITY_DI
GO:001673	molecular_	oxidoreduc	1	4	25	51576	309	TRINITY_DI
GO:004389	molecular_	glyceraldeh	1	5	52	51576	309	TRINITY_DI
GO:005028	molecular_	serine-glyo	1	3	9	51576	309	TRINITY_DI
GO:000476	molecular_	serine-pyru	1	3	9	51576	309	TRINITY_DI
GO:004213	molecular_	fructose 1,(	1	4	27	51576	309	TRINITY_DI
GO:000842	molecular_	beta-gluco:	1	10	310	51576	309	TRINITY_DI
GO:005066	molecular_	NADP bindi	2	9	262	51576	309	TRINITY_DI
GO:005066	molecular_	coenzyme I	1	18	1024	51576	309	TRINITY_DI
GO:000826	molecular_	poly(U) RN.	2	4	35	51576	309	TRINITY_DI
GO:000818	molecular_	poly-pyrim	2	4	39	51576	309	TRINITY_DI
GO:000460	molecular_	peroxidase	2	10	373	51576	309	TRINITY_DI
GO:001616	molecular_	chlorophyll	2	6	123	51576	309	TRINITY_DI
GO:001683	molecular_	carbon-oxy	1	11	493	51576	309	TRINITY_DI
GO:001668	molecular_	oxidoreduc	1	10	426	51576	309	TRINITY_DI
GO:001620	molecular_	antioxidant	1	10	430	51576	309	TRINITY_DI
GO:001683	molecular_	hydro-lyase	1	8	282	51576	309	TRINITY_DI
GO:001673	molecular_	oxidoreduc	1	4	55	51576	309	TRINITY_DI
GO:004690	molecular_	tetrapyrrol	2	19	1295	51576	309	TRINITY_DI
GO:000382	molecular_	catalytic ac	1	203	28978	51576	309	TRINITY_DI
GO:001870	molecular_	thiol S-met	2	2	7	51576	309	TRINITY_DI
GO:000480	molecular_	transketola	1	2	8	51576	309	TRINITY_DI
GO:005027	molecular_	sedoheptul	1	2	8	51576	309	TRINITY_DI
GO:004297	molecular_	glucan end	1	5	126	51576	309	TRINITY_DI

: stress in Agrostis stolonifera

annotation	log_odds_r	p
// // // /,	4.094099	4.40E-65
// // // /,	5.37238	4.50E-42
// // // /,	5.37238	4.50E-42
// // // /,	5.044147	1.57E-38
// // // /,	4.460629	2.96E-29
// // // /,	3.80187	3.81E-23
// // // /,	2.644181	1.84E-17
// // // /,	1.674663	7.45E-15
// // // /,	1.959978	4.15E-14
// // // /,	1.21581	2.78E-09
// // // /,	2.237563	5.61E-09
// // // /,	3.091034	8.52E-09
// // // /,	0.864187	1.49E-08
// // // /,	0.44986	3.98E-08
// // // /,	3.151385	2.17E-07
// // // /,	2.981501	2.68E-07
// // // /,	0.445563	5.48E-06
// // // /,	2.658923	1.40E-05
// // // /,	5.31256	2.76E-05
// // // /,	3.768239	2.89E-05
// // // /,	3.075521	7.25E-05
// // // /,	0.92797	0.000181
// // // /,	2.897522	0.000198
// // // /,	3.517879	0.000475
// // // /,	3.503804	0.0005
// // //	5.061021	0.000817
// // // /,	4.278613	0.000839
// // // /,	4.278613	0.000839
// // // /,	2.545006	0.001344
// // // /,	4.119915	0.001344
// // // /,	4.119915	0.001344
// // // /,	2.923518	0.001582
// // // /,	3.923518	0.002449
// // // /,	3.413323	0.002713
// // // /,	1.520002	0.003461
// // // /,	3.77414	0.003796
// // // /,	3.77414	0.003796
// // // /,	0.243211	0.003936
// // // /,	0.498677	0.005494
// // // /,	2.512585	0.009718
// // // /,	0.79924	0.012032
// // // /,	2.435417	0.033606
// // // /,	0.138009	0.035071

// // // /,	1.82836	0.056376
// // // /,	2.079168	0.064346
// // // /,	2.428753	0.086099
// // // /,	2.704877	0.095477
// // // /,	1.455855	0.096067
// // // /,	2.131323	1.38E-64
// // // /,	2.137367	5.90E-64
// // // /,	2.515315	1.39E-51
// // // /,	2.510077	1.61E-51
// // // /,	3.319592	7.08E-46
// // // /,	3.319592	7.08E-46
// // // /,	3.236267	1.25E-45
// // // /,	3.318819	2.08E-42
// // // /,	3.388686	1.12E-41
// // // /,	3.385815	1.16E-41
// // // /,	3.406408	2.28E-41
// // // /,	3.406408	2.28E-41
// // // /,	2.581412	1.00E-31
// // // /,	0.881141	4.16E-25
// // // /,	2.600618	1.16E-23
// // // /,	2.589269	4.69E-23
// // // /,	0.656174	8.94E-17
// // // /,	3.838629	1.76E-15
// // // /,	2.211244	6.23E-15
// // // /,	2.177128	7.32E-14
// // // /,	4.923518	2.42E-12
// // // /,	0.481596	1.37E-09
// // // /,	0.481825	1.37E-09
// // // /,	0.766592	1.64E-08
// // // /,	0.434527	2.43E-08
// // // /,	0.4348	2.43E-08
// // // /,	0.75651	2.43E-08
// // // /,	1.540189	2.43E-08
// // // /,	1.539643	2.43E-08
// // // /,	3.461337	6.59E-08
// // // /,	3.754918	1.80E-07
// // // /,	0.320992	1.06E-05
// // // /,	0.312494	1.40E-05
// // // /,	1.767107	1.52E-05
// // // /,	1.324056	2.92E-05
// // // /,	1.93009	8.15E-05
// // // /,	3.27675	8.15E-05
// // // /,	3.27675	8.15E-05
// // // /,	3.880449	0.000555
// // //	5.213024	0.000562
// // // /,	2.909018	0.000587
// // // /,	1.990632	0.001054

// // // /,	1.990632	0.001054
// // // /,	2.732695	0.001344
// // // /,	0.187362	0.002713
// // // /,	0.18193	0.003936
// // // /,	2.710524	0.003936
// // // /,	6.061021	5.28E-23
// // // /,	1.253961	1.82E-09
// // // /,	2.131352	2.90E-09
// // // /,	3.088328	2.79E-08
// // // /,	2.675996	5.07E-07
// // // /,	1.743609	7.23E-07
// // // /,	4.923518	9.14E-06
// // // /,	5.004438	8.15E-05
// // // /,	4.846896	0.000137
// // // /,	3.428753	0.000156
// // // /,	1.775267	0.000211
// // // /,	3.135022	0.000629
// // // /,	3.135022	0.000629
// // //	5.135022	0.000665
// // // /,	3.818165	0.000665
// // //	4.859387	0.001344
// // //	4.739093	0.001836
// // // /,	4.004438	0.001936
// //	5.797987	0.002141
// //	5.797987	0.002141
// // //	4.628062	0.00242
// // // /,	2.428753	0.002469
// // // /,	2.519451	0.003772
// // // /,	1.552874	0.006012
// // //	4.253666	0.006075
// // //	4.097547	0.009281
// // // /,	2.161846	0.010059
// // // /,	3.025397	0.010675
// // // /,	1.896937	0.021927
// // // /,	1.970168	0.028024
// // // /,	1.956684	0.029914
// // // /,	2.243398	0.03223
// // //	3.60159	0.032768
// // // /,	1.29214	0.033606
// // // /,	0.225615	0.037073
//	5.575594	0.06738
//	5.382949	0.08745
//	5.382949	0.08745
// // // /,	2.727597	0.089662
